# Proceedings 36th Symposium ESVN‐ECVN 12th‐14th September 2024

**DOI:** 10.1111/jvim.17225

**Published:** 2024-11-07

*The European College of Veterinary Neurology (ECVN) Symposium and the Journal of Veterinary Internal Medicine (JVIM) are not responsible for the content or dosage recommendations in the abstracts. The abstracts are not peer reviewed before publication. The opinions expressed in the abstracts are those of the author(s) and may not represent the views or position of the ECVN. The authors are solely responsible for the content of the abstracts*.

**RESIDENTS DAY PROGRAM**

**12 SEP 2024 | THURSDAY**

**Table:** 

**08.00 AM**	**Registration**
**09.00 AM**	**Welcome and Introduction**
**09.15 AM**	**Visual Pathways**
	Elsa Beltran
	Daniel Sanchez‐Masian
**10.00 AM**	**How to do a Useful Ophthalmological Examination**
	Claudia Hartley
	Daniel Sanchez‐Masian
**10.45 AM**	**Coffee Break**
**11.30 AM**	**Cranial Nerve Anatomy**
	Elsa Beltran
	Camilla Cooper
**12.15 PM**	**What Imaging Modality to do for Investigation of Blindness**
	Fraser McConnell
	Camilla Cooper
**1.00 PM**	**Lunch**
**2.30 PM**	**Diseases Affecting Vision**
	Elsa Beltran
	Stephanie Phillipps
**3.15 PM**	**Tips/Pitfalls for Advanced Imaging Interpretation**
	Fraser McConnell
	Stephanie Phillipps
**7.30 PM**	**Welcome Reception**
**9.00 PM**	

**ESVN‐ECVN 36th Symposium: Neuro‐Ophthalmology**

**PROGRAM**

**13 SEP 2024 | FRIDAY**

**Table:** 


**08.45 AM**	**Welcome**
**09.00 AM**	**Acute Blindness**
	Claudia Hartley
	Raquel Trevail
**09.40 AM**	**Optic Neuritis/NMO**
	João Lemos
	Raquel Trevail
**10.20 AM**	**Coffee Break and Exhibition**
**11.00 AM**	**Oral Presentations**
	Jacques Penderis
	COMPARATIVE STUDY OF INTRANASAL DIAZEPAM AND MIDAZOLAM AS RESCUE TREATMENT IN DOGS WITH EPILEPTIC SEIZURES
	**Jose Luis Caldera;** Jose Ignacio Cristóbal; Ángela Durán; Paloma Nicolás; Javier Duque; Alba Díaz; Irene Cantalejo; Inmaculada Sevidane
	ULTRA‐LONG‐TERM ELECTROENCEPHALOGRAPHIC MONITORING DEVICE IMPLANTATION IN DOGS
	**Casey Rogers;** Nina Meyerhoff; Sebastian Meller; Holger Volk
	SINGLE DOSE PHARMACOKINETICS OF BRIVARACETAM IN HEALTHY DOGS
	**Tom Jukier;** Kamoltip Thungrat; Robert Arnold; Chu Zhang; Amanda Gross
	REM SLEEP BEHAVIOR AND URINARY RETENTION DISORDER IN RUSSIAN BLUE CATS
	**Kimberley Stee;** Mario Van Poucke; Ariel Cohen‐Solal; Leen Van Brantegem; Kaatje Kromhout; Sofie Fm Bhatti; Luc Peelman; Ine Cornelis
	HYPERTHYROIDISM‐ASSOCIATED PAROXYSMAL DYSKINESIA IN THREE CATS: A NOVEL METABOLIC ENCEPHALOPATHY
	**Javier Espinosa;** Inés Martínez; Irene Espadas; Annette Wessmann; Juan José Minguez; Christoforos Posporis; Patricia Álvarez
**11.50 AM**	**Under Pressure**
	Fraser McConnell
	Jacques Penderis
**12.30 PM**	**Lunch Break, Exhibition and Poster Presentation**
**01.30 AM**	**Abnormal Eye Movements**
	João Lemos
	Sebastien Behr
**02.10 AM**	**Flash Poster Presentations**
	Sebastien Behr
	MASTICATORY MUSCLE CHANGES ON MAGNETIC RESONANCE IMAGING OF DOGS WITH NEOSPORA CANINUM COMPARED TO MENINGOENCEPHALITIS OF UNKNOWN ORIGIN
	**Jessica Zilli**; Kathryn Fleming; Chloe Fischer; Tim Sparks; Tom Harcourt‐Brown; Ed Ives
	MAJOR COMPLICATIONS ASSOCIATED WITH CEREBROSPINAL FLUID COLLECTION IN DOGS: CLINICAL PRESENTATION AND IMAGING CHARACTERISTICS IN 10 DOGS
	**Cecilia‐Gabriella Danciu;** Alana Mccarthy; Abbe Crawford
	PAROXYSMAL DYSKINESIA IN 41 DOGS: IS THE DIET CHANGE ENOUGH?
	**Marika Menchetti;** Evelina Burbaitè; Greta Galli
	EVALUATION OF CYCLOSPORINE AND PREDNISOLONE IN COMPARISON TO PREDNISOLONE ALONE FOR THE TREATMENT OF SUSPECTED MENINGOMYELITIS OF UNKNOWN ORIGIN IN 47 DOGS
	**Tania Al Kafaji;** Andrea Corda; Ilaria Tartari; Antonella Gallucci
	PRESUMPTIVE MENINGOENCEPHALOMYELITIS OF UNKNOWN ORIGIN IN GERIATRIC DOGS
	**Tamara Heredia;** Christian Maeso; Elisabet Domínguez; Emili Alcoverro
	CANINE MENINGOENCEPHALITIS OF UNKNOWN ORIGIN: OBJECTIVE EVALUATION BY MAGNETIC RESONANCE IMAGING USING THE CAVALIERI METHOD
	**Franziska Spohn;** Holger Volk; Adriano Wang‐Leandro; Filip Kajin; Jasmin Nessler
	PRESENCE OF ANTI‐TRIM46‐AUTOANTIBODIES IN SERUM AND CEREBROSPINAL FLUID FROM A DOG PRESENTING WITH MENINGOENCEPHALITIS OF UNKNOWN ORIGIN
	**Pernille Lindholm Heidemann;** Anna Christine Nilsson; Sandra Gaedt Schmidt; Morten Blaabjerg; Hanne Gredal
**02.40 PM**	**Oral Presentations**
	Sebastien Behr
	A COMPARISON OF THE PREVALENCE AND NATURE OF COMPLICATIONS IN FRENCH BULLDOGS AND DACHSHUNDS WITH THORACOLUMBAR OR LUMBAR INTERVERTEBRAL DISC EXTRUSION
	**Eleftheria Skovola;** Steven De Decker
	ASSOCIATION OF THE APPEARANCE OF CSF ON HASTE MRI WITH CLINICAL FACTORS AND SHORT‐TERM OUTCOME IN FRENCH BULLDOGS PRESENTING WITH THORACOLUMBAR INTERVERTEBRAL DISC EXTRUSION
	**Anastasia Morgan;** Nicholas Grapes; Steven De Decker; Robert Brash; Jessica Milne
	THE EFFECTS OF FORAMINOTOMY AND DISTRACTION‐STABILIZATION ON THE VOLUME OF THE LUMBOSACRAL NEUROFORAMEN THROUGHOUT SPINAL MOTION
	**Raphael Arz;** Brian Park; Sebastian Knell; Frank Steffen; Lucas Adam Smolders
	TREATMENT OF PRESUMED ACUTE THORACOLUMBAR INTERVERTEBRAL DISC HERNIATION WITH INTRADISCAL CHONDROITINASE ABC
	**Nick Jeffery;** Paul Freeman
	MINIMALLY INVASIVE BIPORTAL ENDOSCOPIC FORAMINOTOMY OF L7‐S1 IN DOGS: EX VIVO ASSESSMENT AND CLINICAL CASE REPORT
	**Dimitrios Bekiaridis;** Antonio Pozzi; Frank Steffen; Julien Guevar; Lucas Smolders
**03.30 PM**	**Coffee Break and Exhibition**
**04.00 PM**	**AGM**
**07.30 PM**	**Gala Dinner**
**14 SEP 2024 | FRIDAY**	
**08.45 AM**	**Welcome**
**09.00 AM**	**Oral Presentations**
	Sebastien Behr
	THE DIAGNOSTIC YIELD OF WHOLE‐GENOME SEQUENCING IN DOGS AND CATS WITH SUSPECTED NEUROGENETIC DISORDERS
	**Rodrigo Gutierrez Quintana;** Julien Guevar; Vidhya Jagannathan; Tosso Leeb
	ASSESSMENT OF HYPERCOAGULABILITY IN DOGS WITH ISCHAEMIC STROKE
	**Sophie Wyatt;** Stefano Cortellini
	NEURO‐OPHTHALMOLOGICAL FINDINGS OF HYPOVITAMINOSIS A IN BEEF CATTLE: A RETROSPECTIVE STUDY
	**Giulia Cagnotti;** Cristina Giordano; Giorgia Di Muro; Sara Ferrini; Chiara Giudice; Giuliano Borriello; Antonio D'angelo
**09.40 AM**	**Investigation of Acute Blindness**
	Claudia Hartley
	Abbe Crawford
**10.20 AM**	**Coffee Break and Exhibition**
**11.00 AM**	**Nystagmus Testing**
	João Lemos
	Gemma Walmsley
**11.40 AM**	**Oral Presentations**
	Gemma Walmsley
	TREMORS IN CATS: 105 CASES (2004‐2023)
	**Theofanis Liatis;** Sofie F.M. Bhatti; Steven De Decker
	MRI FINDINGS IN CATS WITH HISTOLOGICALLY CONFIRMED OR SEROLOGICALLY SUSPECTED CENTRAL NERVOUS SYSTEM TOXOPLASMOSIS
	**Emmeline Robinson;** Maria Ines De Freitas; Daniela Alder; Ezio Bianchi; Kendall E Day; Alice Dussaux; Antonio Filippi; Sergio A Gomes; Theofanis Liatis; Lauren Marini; Malte Schwartz; Josh D Warren; Sophie Elizabeth Wyatt; Lorenzo Mari
	CORRELATION BETWEEN CLINICAL IMPROVEMENT AND IMAGING FINDINGS POST‐TREATMENT IN CATS WITH NEUROLOGICAL FELINE INFECTIOUS PERITONITIS TREATED WITH ANTIVIRALS
	**Ines Martinez‐Garcia;** Rodrigo Gutierrez‐Quintana; Luciano Espino‐Lopez; Francisco Flores‐Ferrero; Anna Suñol‐Iniesta; Koen M. Santifort; Alex Kennedy; Daniel Sanchez‐Masian
	EFFECT OF A SINGLE DOSE OF INTRAMUSCULAR METHADONE ADMINISTRATION ON NEUROLOGICAL EVALUATION OF DOGS AFFECTED BY INTERVERTEBRAL DISC HERNIATION
	**Giorgia Stufano;** Massimiliano De Vittor; Delia Franchini; Gianvito Lanave; Barbara Betti; Michela Scuttari; Federica Tirrito
**12.20 PM**	**Presentation ESVN/ECVN Annual Meeting 2025**
**12.30 AM**	**Lunch Break, Exhibition and Poster Presentation**
**01.30 PM**	**Sustainability in Veterinary Practice**
	Ellie West
	Rita Gonçalves
**02.06 PM**	**Flash Poster Presentations**
	Rita Gonçalves
	BRAIN‐RELATED PRESENTATIONS ARE MORE PREVALENT IN BRACHYCEPHALIC DOGS WITH ‘BRACHYCEPHALIC OBSTRUCTIVE AIRWAY DISORDER’ (BOAS).
	**Susana Monforte Monteiro;** Lisa Alves
	NEUROFILAMENT LIGHT CHAIN (NF‐L) QUANTIFICATION IN CEREBROSPINAL FLUID AND SERUM AS A POSSIBLE BIOMARKER IN THE DIFFERENTIAL DIAGNOSIS OF BOVINE NEUROLOGICAL DISEASES
	**Giorgia Di Muro;** Carlotta Tessarolo; Giulia Cagnotti; Alessandra Favole; Sara Ferrini; Ugo Ala; Claudio Bellino; Giuliano Borriello; Claudia Palmitessa; Giulia Iamone; Barbara Iulini; Marzia Pezzolato; Cristiano Corona; Antonio D'angelo
	SEE‐SAW LIKE NYSTAGMUS IN AN MRI CONFIRMED ACHIASMATIC GOLDEN RETRIEVER DOG
	**Alexine Lajoie;** Jérôme Couturier; Eddy Cauvin
	NEURONAL CEROID LIPOFUSCINOSIS (NCL7) IN SMALL SWISS HOUNDS CAUSED BY AN INTRAGENIC MFSD8 DUPLICATION
	**Loderstdt Shenja;** Rietmann Stefan; Matiasek Kaspar; Kiefer Ingmar; Leeb Tosso
	CLINICAL APPLICATION OF 3D‐PRINTED GUIDES WITH SPECIFIC CRANIAL CONTOUR FOR BRAIN NEOPLASIA REMOVAL IN DOGS.
	**Sergio Moya;** Christian Maeso; Nuria Delgado; Juan Carlos Cartagena; Victoria Moya; Sergio Rodenas
	UNRELIABLE CLINICAL TEST RESULT OF CHROMATIC PUPILLARY LIGHT REFLEXES IN TWO DOGS WITH AMAUROSIS SECONDARY TO OPTIC PATHWAY DISORDERS—A CAVEAT FOR CLINICAL APPLICATION
	**Koen Santifort;** Marta Plonek; Christiane Görig; Ingrid Kraijer‐Huver
	NEAR INFRARED FLUORESCENT IMAGE‐GUIDED BIOPSY OF A SCIATIC NERVE TUMOR IN A DOG
	**Athina Karpozilou;** Filipa Lourinho; Beatriz Moreno Aguado; Diogo Miraldo
	DORSAL ATLANTOAXIAL STABILIZATION USING A PEDICLE SCREW FIXATION SYSTEM IN TWO DOGS
	**Guillaume Leblond;** Natalie West; Rodrigo Gutierrez‐Quintana
	A BEACON OF HOPE’ IN THE AI HYPE STORM: A DEEP LEARNING MODEL FOR TRIAGING CANINE BRAIN MRI ABNORMALITIES
	**Samira Abani; Holger Volk;** Merlin Laue; Peter Dickinson; Steven De Decker; Rodrigo Gutierrez‐ Quintana; Alexej Alexej Hänsch; Gloria Lesta; Aleksandr Subbotin; Kseniia Bocharova; Jörg Janisch; Laura Lemke; Franziska Spohn; Stefanie Winzer; Daniel Sánchez‐Masian; Ehren Mclarty; Adriano Wang‐Leandro; Jasmin Nessler
	LONG‐TERM FOLLOW‐UP OF SURGICAL MANAGEMENT IN PUG DOGS WITH THORACOLUMBAR MYELOPATHIES AND CONCURRENT ARTICULAR FACET DYSPLASIA.
	**Anna Tauro;** Colin Driver; Jeremy Rose; Ricardo Fernandes; Clare Rusbridge
	ROLE OF INTRAVENOUS INTRAOPERATIVE MAGNESIUM SULFATE AS ANTI‐INFLAMMATORY AND ANALGESIC DRUG IN DOGS UNDERGOING SPINAL DECOMPRESSION SURGERY FOR ACUTE THORACOLUMBAR DISC HERNIATION
	**Guillaume Mallet;** Luis Gaitero; Andrea Sanchez Lazaro; Fiona Mk James; Gabrielle Monteith; Geoffrey Wood; Francesca Samarani
**02.50 PM**	**Oral Presentations**
	Rita Gonçalves
	POLYMYOSITIS IN THE DUTCH KOOIKER DOG
	**Paul Mandigers;** Yvet Opmeer; Vanessa Alf; G. Diane Shelton; Frank Van Steenbeek; Claudia Rozendom; Peter Van Kooten; Victor Rutten; Guy Grinwis; Marjo Hytönen; Marco Rosati; Hille Fieten; Hannes Lohi; Peter Leegwater; Kaspar Matiasek
**02.50 PM**	**Oral Presentations**
	Rita Gonçalves
	ACCURACY OF CLINICAL DIAGNOSIS OF MENINGOENCEPHALITIS OF UNKNOWN ORIGIN IN DOGS: A RETROSPECTIVE STUDY
	**Jasmin N. Nessler;** Karen M. Vernau; Christine M. Toedebusch; Kevin D. Woolard; Andreas Beineke; Peter J. Dickinson
	CARE PATHWAY DECISION SUPPORT IMPROVES GP VET DECISION MAKING IN CANINE MYELOPATHY
	**Katie Fox;** Paul Freeman
	MIRNA EXPRESSION PROFILE IN THE CSF OF DOGS DIAGNOSED WITH DEGERATIVE MYELOPATHY
	**Dominik Faissler;** Elissa Williams; Dawn Meola; Vicky Yang
**03.30 PM**	**Coffee Break and Exhibition**
**04.00 PM**	**Flash Poster Presentations**
	Thomas Flegel
	CLINICAL PRESENTATION AND MRI FINDINGS OF CEREBELLAR DISEASE IN DOGS
	**Luis Villalonga Rodriguez;** Pablo Amengual‐Batle; Vicente Aige Gil; Rita Gonçalves; Albert Aguilera Padros; Kiterie Me Faller; Christian Maeso Ordas; Anna Suñol Iniesta
	LAMA‐2 RELATED CONGENITAL MUSCULAR DYSTROPHY IN A CAT: CLINICAL AND GENETIC FINDINGS
	**Christopher Skinner;** Cleo Schwarz; Rodrigo Gutierrez Quintana; G. Diane Shelton; Angelika Rupp; Tosso Leeb; Adriana Kaczmarska
	BILIRUBIN ENCEPHALOPATHY IN FOUR CATS FOLLOWING XENOTRANSFUSION
	**Abbe Crawford;** Cecilia Danciu; Simon Cook
	EVALUATION OF BLOOD C REACTIVE PROTEIN (CRP) AND NEUTROPHIL‐TO‐LYMPHOCYTE RATIO (NLR) AS A DIAGNOSTIC TOOL IN CANINE EPILEPSY
	**Andreea Despa;** Mihai Musteata; Gheorghe Solcan
	MRI CHARACTERISTICS OF THE INNER EAR ON POST‐CONTRAST FLUID ATTENUATED INVERSION RECOVERY IMAGES IN DOGS WITHOUT MIDDLE AND INNER EAR DISEASE
	**Ya‐Pei Chang;** Shi‐Yi Wang
	IDIOPATHIC FACIAL NEUROPATHY IN CATS: CLINICAL FINDINGS, MRI FINDINGS AND OUTCOME
	**Maria Stefaniuk;** Cecilia Gabriella Danciu; Laura Muñiz Moris; Cristoforo Ricco; Steven De Decker; Josep Brocal; Emili Alcoverro; Oliver Marsh; Frances Taylor‐Brown; Lorenzo Mari
	EX VIVO BIOMECHANICAL ASSESSMENT OF SURGICAL STABILIZATION TECHNIQUES OF THE LUMBOSACRAL JUNCTION IN DOGS
	**Lucas Smolders;** Raphael Arz; Antonio Pozzi; Brian Park
	OWNER COMPLIANCE, AND BARRIERS TO COMPLIANCE, WITH CAGE REST, PHYSIOTHERAPY AND HYDROTHERAPY, IN THE CONSERVATIVE MANAGEMENT OF DOGS DIAGNOSED WITH INTERVERTEBRAL DISC DISEASE
	**Brett Noden;** Samantha Bell; Anisa Ab Razak; Lisa Alves
	AN ‘ALTERNATING SMILE SIGN’ IN TWO DOGS THAT MAY RESEMBLE DYSTONIC FACIAL SEIZURES
	**Callum Atkins;** Joao Miguel De Frias; Theofanis Liatis; Catherine Stalin; Liam Wilson; Tessa Proctor; Eric Glass
**04.00 PM**	**Flash Poster Presentations**
	Thomas Flegel
	AN ‘ALTERNATING SMILE SIGN’ IN TWO DOGS THAT MAY RESEMBLE DYSTONIC FACIAL SEIZURES
	**Callum Atkins;** Joao Miguel De Frias; Theofanis Liatis; Catherine Stalin; Liam Wilson; Tessa Proctor; Eric Glass
	THE USE OF 3D‐CISS AND CINE MRI SEQUENCES IN A DOG WITH OBSTRUCTIVE HYDROCEPHALUS, AND THEIR ROLE IN SURGICAL PLANNING.
	**Anna Tauro;** John Macri; Peter Early; Christopher Mariani; Natasha Olby; Karen Munana; Melissa Lewis; Linda Dillenbeck
	MYELOPATHY IN THREE CATS WITH COBALAMIN AND FOLATE DEFICIENCY
	**Christopher Skinner;** Rodrigo Gutierrez Quintana; Anne R. Fraser
**04.50 PM**	**Awards and Closing Remarks**

*Consensus Statements of the European College of Veterinary Neurology (ECVN) provide the veterinary community with up‐to‐date information on the pathophysiology, diagnosis, and treatment of clinically important animal diseases. The ECVN Board oversees selection of relevant topics, identification of panel members for each topic with the expertise to draft the statements, and other aspects of assuring the integrity of the process. The statements are derived from evidence‐based medicine whenever possible and the panel offers interpretive comments when such evidence is inadequate or contradictory. A draft is prepared by the panel, followed by solicitation of input by the ECVN membership which may be incorporated into the statement. It is then submitted to the Journal of Veterinary Internal Medicine, where it is edited prior to publication. The authors are solely responsible for the content of the statements*.

## ACUTE BLINDNESS

## Claudia Hartley BVSc CertVOphthal DipECVO FHEA FRVCS

**Royal (Dick) School of Veterinary Medicine, University of Edinburgh, Easter Bush Campus, EH25 9RG**

This talk will focus on ocular and orbital causes of blindness (i.e., excluding central causes of acute blindness).

## Uveitis (Infectious/inflammatory)

This will need to be severe to cause blindness (as opposed to visual deficits). Infectious causes include: Prototheca, Cryptococcosis, Histoplasma, Blastomycosis, Coccidiomycosis, Aspergillosis, Leishmania, Rabies, Distemper, FeLV/FIV, Toxoplasma, Neospora, Ehrlichia, Rickettsia, Babesia, Bartonella. Inflammatory or immune mediated causes include: a septic focus (e.g., pyometra), hyphaema (e.g., traumatic, Angiostrongylus, systemic hypertension, anti‐coagulant poisoning), diabetes mellitus (e.g., acute cataract formation), Neoplasia—primary/secondary and Feline Infectious Peritonitis (FIP).

## Acute cataract formation (e.g., diabetes mellitus, persistent foetal vasculature)

Hyperglycaemia associated with diabetes, and as glucose is a small molecule it will pass into the lens (along with all other tissues). Here it overwhelms the normal hexokinase pathway and excess is shunted to aldose reductase pathway where the end product is sorbitol (a large molecule and therefore trapped within lens capsule) resulting in osmotic draw and tumescent cataract formation. This can be exceptionally rapid where glycaemic control is poor, and may even result in lens capsule rupture and phacoclastic uveitis (requiring emergency intervention to save the globe).

Persistent hyperplastic primary vitreous or persistence of foetal hyaloid system may result in intralenticular hemorrhage and can be associated with retinal detachment.

## Glaucoma

May be classified as primary (inherited, no antecedent ocular disease) or secondary (e.g., chronic uveitis or lens luxation). Tonometry is essential for diagnosis (see Investigation of Acute Blindness). In dogs the classic presentation is diffuse corneal oedema with dilated pupil and absent menace response. In contrast, with cats the presentation can be more subtle with merely a dilated pupil (anisocoria) and little corneal oedema. They may also retain vision for longer.

## Progressive Retinal Atrophy (PRA)

Vision loss in not usually acute, however, some owners may not notice until move house/furniture as dog/cat has adapted. Initial presenting sign is usually nyctalopia (most start with rod de.g.eneration followed by cones). Some cases will develop secondary cataract. Funduscopic signs are tapetal hyperreflectivity and vascular attenuation as the disease progresses. Initially may require ERG to diagnose (see Investigation of Acute Blindness).

## Sudden Acquired Retinal Degeneration Syndrome (SARDS)

Acute & permanent vision loss so clinical presentation is typically menace negative, mydriatic pupils with sluggish PLR (N.B. may have positive dazzle and PLRs [esp early]). Mean age at onset is 8.5–10 years (usually 7–‐10 years range). Typically older spayed females (60%–90% in some studies, others found no predisposition Heller et al 2017), and small breed dogs (42% Heller et al 2017). A seasonal onset has been reported (46% of cases in Dec & Jan). Retinal examination is usually normal in the acute phase with diffuse signs of atrophy as degeneration continues (6–8 weeks) similar to those seen with PRA.

Some (40%–60%) cases have a history of Cushingoid like signs (PU/PD/PP, wt gain, and skin/hair coat changes), but can be negative/equivocal on testing (only 12%–17% positive). These signs often precede vision loss, and these usually resolve (PP may increase over first year before recedes). Hypothyroidism and diabetes mellitus are reported co‐morbidities in some (Stuckey et al. 2013).

Some reports of cases with concurrent hearing loss, sense of smell reduction (Abrams et al. 2023), and/or change in behavior.

Blood biochemistry abnormalities include: increased serum cortisol in 9/11 cases, increased in one or more 1 sex hormones in 11/13 cases in one study, raised cholesterol and/or ALKP was reported in 68% of cases.

Histopathology has revealed the first lesion is a loss of photoreceptor (PhR) outer segments, followed by apoptosis of PhRs and finally full retinal thickness atrophy.

Aetiopathogenesis has not been fully elucidated but an immune‐mediated basis has been proposed. Anti‐retinal antibodies have been found in SARDS patients but there are contradictory studies with some reporting no higher incidence of anti‐retinal antibodies in SARDS vs healthy patients. Immunohistochemistry has revealed immunoglobulin producing plasma cells in affected retinas, and complement activity suggestive of antibody‐mediated neuronal damage has been proposed as a mechanism of the syndrome (Grozdanic et al 2008 Vet Clin N Am). In humans, autoimmune retinopathy (AIR) has been described, and a sub‐group of this population—‘paraneoplastic AIR’ or ‘Cancer‐associated retinopathy (CAR)’ however, there is no evidence to support this in canine SARDS patients.

A neuroendocrine etiology has also been proposed due to concurrent reports of loss of sense of smell, and a pituitary derived factor has been suggested.

The prognosis for vision is grave and treatments reported (including immunosuppression—mycophenolate mofetil—no benefit (Young et al 2018) after 6 weeks therapy, or combination immunosuppressive meds (lifelong)—no controlled prospective studies) are not currently supported by scientific evidence. Most dogs do adjust (Stuckey et al 2013) with good QoL; ‘Living with Blind Dogs’ is a good resource for owners.

A nice review paper summarizing the current knowledge of SARDS (Komaromy et al 2015 Vet. Ophth).

## Retinal toxicities

Drug/Chemical‐induced retinotoxicity has been reported with enrofloxacin in cats (especially elderly and/or renally impaired, and especially at doses >5 mg/kg), closantel (anthelmintic used predominantly in farm animals) overdose in farm species and dogs with accidental exposure, ivermectin (commonly dogs exposed via equine wormers [can be after eating horse stools], but can be for some mange treatments). Other drugs include: rafoxanide, quinine (RGC loss, optic atrophy), chlorquine (RGC damage), digoxin (reversible cone dysfunction), metal‐chelating agents (diphenylthiocarbazone, hydroxpyridinethione).

Phototoxicity (o2 free radicals) is also capable of causing retinal degeneration, as well as high oxygen tensions (human retinopathy of prematurity [ROP]). Radiation induced retinopathy (degenerative angiopathy, multifocal retinal hemorrhages, retinal degeneration usually starting in outer retina)—NB cataracts may obscure fundus examination.

## Retinal detachment

Causes of retinal detachment include: immune mediated (steroid responsive serous), rhegmatogenous, systemic hypertension, subretinal infection or neoplastic infiltration and traumatic (often rhegmatogenous).

## Optic neuritis

Intraocular optic neuritis or retrobulbar (orbital) optic neuritis. Funduscopic changes optic nerve head swelling, retinal vascular engorgement, and peripapillary retinal oedema and/or separation. Foci of chorioretinitis (especially peripapillary) are seen in some cases.

Optic neuropathy can result from trauma to optic nerve and shearing forces within the optic canal. These injuries may be associated with optic nerve avulsion (avulsed posteriorly), basal skull fractures and other cranial nerve deficits, and/or brain stem injuries.

Optic neuropathy can be encountered secondary to proptosis. The trauma required to proptose the brachycephalic globe can be relatively minor, whilst this is not the case in dolicocephalic breeds or cats where extensive trauma is usually required. Prognosis for vision is generally poor at roughly 20%, however the globe can often be salvaged for cosmetic purposes by early intervention and treatment. Multiple extraocular muscle avulsion and total hyphaema cases have a poorer prognosis. The medial rectus muscle is the shortest muscle—first to be avulsed. Pupil size is not a prognostic indicator for vision, although those eyes with a direct and/or indirect pupillary light reflex (PLR) and/or a positive menace response obviously have a better prognosis for vision.

Vision at last recheck was not correlated with breed, cause or duration of proptosis, or post‐operative medications (*P* > .05), but was correlated with presence of direct and indirect pupillary light reflexes (PLRs) on admission (*P* = .001 and .02, respectively), and with assessment and surgery performed by veterinary ophthalmologists rather than surgery or emergency personnel (*P* = .015).

Optic neuropathy can also be seen due to ischaemia associated with interrupted vascular supply (more common in humans)—massive blood loss or ligation of carotid arteries (esp. horses).

## Orbital compartment syndrome

Compression of the optic nerve due to retrobulbar disease (severe orbital cellulitis, neoplastic infiltration/mass effect) may result in blindness.

In humans this is a more common condition secondary to Graves' disease (hyperthyroidism)—relative proptosis, eyelids that are difficult to open with finger pressure, and the presence of an RAPD (Relative Afferent Pupillary Defect) in the traumatized eye are surgical decompression emergencies (BJ Oral & Maxfac Surg 2020). The coronal approach has been reported as the best for post op visual acuity in humans.

## INVESTIGATION OF ACUTE BLINDNESS

## Claudia Hartley BVSc CertVOphthal DipECVO FHEA FRVCS

**Royal (Dick) School of Veterinary Medicine, University of Edinburgh, Easter Bush Campus, EH25 9RG**

History, Signalment & Ophthalmic examination (see resident's day notes)

## Tonometry

It is essential when handling cases of suspected glaucoma. Normal in cats and dogs 10–25 mmHg, horses 15–30 mmHg. Tonometry is also useful when monitoring uveitis cases—these can develop glaucoma as a complication, and the intraocular pressure also reduces in many cases of uncomplicated uveitis.

Schiøtz tonometer This is an indentation tonometer and inexpensive. The patient's head is positioned vertically (nose to ceiling) and the Schiotz footplate is placed on the anesthetized cornea. Multiple readings are taken to obtain an average/fairly consistent reading. Different weights may need to be added to neck of the plunger if the IOP is elevated. The IOP is read from a conversion chart. There are drawbacks to the technique; it is not entirely accurate and should not be used after intraocular surgery or diseased corneas. It requires topical anesthesia and a compliant patient. It cannot be used on horses (getting the head vertical not possible). Corneal oedema also artificially lowers the IOP reading.

Applanation tonometry using for example, the “Tonopen” (Carleton Optical) tonometer is one of the methods of choice. The technique measures the force required to flatten a given, small area of the corneal surface. It is fairly accurate for use in small animals and the patient can be examined in a normal sitting position (head horizontal). A Tonopen cover must be used on the tip at all times as service/repair is often only just short of the purchase price. Topical anesthesia is required and it is important not to press on the globe through the eyelids (easy to do in horses and cattle when restraining) as an artificially increased reading will be obtained. Increased systolic blood pressure (fear, stress) or pressure on the jugular veins and carotid arteries (N.B. restraint of patient) may also result in an increased reading. General anesthesia may reduce IOP readings (usually by reducing systolic BP).

Rebound tonometry (e.g., “TonoVet”—iCARE) is a technique in which a small probe is fired from an instrument held close to the eye and perpendicular to the un‐anesthetized cornea (needs to be in a horizontal (or near horizontal) plane. The deceleration of the probe as it returns into the instrument is proportional to the intraocular pressure. The technique is accurate, and has been calibrated for the dog/cat and horse (separate settings). As the probe is small, it is ideal for use in small eyes and does not require topical anesthesia. Does not require topical anesthesia and less risk of an iatrogenic corneal ulcer (especially if repeated measurements).

## Ocular ultrasound

Ultrasonography is useful for:

- Investigating orbital/retrobulbar disease, for example, in cases of exophthalmos, enophthalmos, strabismus, optic nerve swelling etc.
- Assessing the status of intraocular structures (e.g., lens position, possible intraocular masses / foreign bodies, presence of vitreal abnormalities and retinal detachments) especially where opacities preclude direct visualization
- Measurement of globe size (e.g., in cases of hydrophthalmos (stretched globe) secondary to glaucoma or microphthalmia (congenitally small eye) as part of a multiple ocular defect syndrome). Various units are available. The main requirements are B‐mode (A‐mode may be useful for biometry), a sector (rather than linear) scanner and a probe of at least 7.5 MHz—ideally 10–20 MHz to enhance image quality. Larger eyes require lower frequency probes (e.g., horse) whereas smaller eyes require higher frequencies ± stand‐offs. Ultrasonography can be performed in most domesticated patients (dog, cat, rabbit, and horse) either fully conscious or using light sedation, but will require anesthesia in most zoo/wild species. A topical anesthetic is applied and viscous coupling gel is required to act as a small stand‐off, allowing imaging of the anterior segment structures. Do not press the probe onto the eye but simply contact it to the coupling gel to get the best images.
  High‐resolution ultrasound (HRU) (20–50 MHz) and ultra‐high resolution ultrasound biomicroscopy (UBM) (50–100 MHz) have been used with excellent results for imaging the eye. HRU probes are available for a number of ultrasound machines and can be used in a similar fashion to that described above. UBM provides resolution of structures as small as 50 um but only has a penetration of 4–5 mm. In larger eyes these will give high‐resolution images of the anterior segment only, but can allow distinction of corneal lesion depth (e.g., sequestrum) or information on structures that we cannot visualize (e.g., drainage apparatus beyond the pectinate ligament—trabecular and uveoscleral meshworks). With UBM, the probe tip is placed in a water bath of methylcellulose and saline filled eye‐ cup, and patient positioning is critical for obtaining a good quality image, so anesthesia or heavy sedation is required. The expense of an UBM machine limits the use of this modality.
  More recent studies using an intravenous contrast agent (composed of ‘microbubbles’) have been used with brilliant results to identify vascularised structures within the eye. Vascularised structures will be highlighted by the flow of these bubbles (seen as hyperechoic signals) immediately after injection of the contrast agent. With respect to the eye, this has been especially helpful in distinguishing between posterior vitreal detachment and retinal detachment, vascularisation of ocular tumors (prior to planned biopsy) and persistent foetal vasculature (e.g., hyaloid artery) prior to planned cataract surgery.

## Colorimetric PLRs

Melanopsin containing RGCs subpopulation which are photosensitive to short wavelength light (480 nm, blue) whereas red light (630 nm) reflects functional photoreceptors and their ganglion cells. SARDS cases would be expected to have a positive blue light reflex but negative red light reflex; whereas optic nerve disease would be expected to have negative blue light and red light reflexes. However, Terakado et al (2013) reported 75% SARDS cases responded as predicted, with 12.5% weak red light PLR, positive blue light and 12.5% weak blue light PLR negative red light PLR, so ERG remains the gold standard for diagnosis of SARDS.

## Electroretinography (ERG)

An ERG involves measurement of retinal generated electrical activity in response to light stimuli. The technique is usually performed on an anesthetized patient using standardized conditions (dark and light adaptation for set periods) and recording equipment when documenting retinal degenerations or functional defects—if requiring objective measurements (ISCEV standardized protocol: Robson et al 2022). More sophisticated measurements using light of varying intensity, wavelength and flashing flicker frequency are more often used in research environments.

Clinically however, it can be useful for crudely assessing retinal function in the presence of clouded ocular media (e.g., cataract) prior to surgery, to rule out retinal degeneration that would make the surgery pointless. Under these circumstances a response to a bright white light flash/flashes is measured and can be performed conscious in most amenable dogs and cats.

Patient preparation requires mydriasis, topical anesthesia, ± sedation/anesthesia, dark adaptation (20 min). It is important to avoid retinal photography/indirect ophthalmoscopy before (min 1 h wait) as bright lights will reduce ERG responses for a prolonged period. For equine ERG—auriculopalpebral nerve block for eyelid akinesia, detomidine sedation, eye lubricant.

A Ganzfeld or mini‐Ganzfeld stimulator is used to create light stimulation—these require calibration/validation (LEDs) and provide uniform retinal stimulation for reliable/reproducible results. The electrodes required are:

Retinal—JET*/DTL (positive)—topical anesthesia Reference—3 cm behind canthus (negative) Ground—nuchal crest/forehead (common).

Two channels will allow simultaneous bilateral measurements if you using a Ganzfeld or have two mini‐Ganzfeld for each eye. An impedance meter (<5 kΩ) is present with most system that informs on electrode placement and good contact.

There are ISCEV ERG experimental protocols—as differing anesthetic drugs may influence the amplitudes and implicit times. Anesthesia/sedation will generate reduce muscle movement ‘noise’. A Faraday cage—keep away from high‐powered electric motors (AC interference) can be useful but expensive and limited to certain facilities. Signal averaging increases signal to noise ratio, especially when using low‐intensity flashes and expected lower amplitude recordings.

## Multifocal ERGs

These can provide geographic mapping of retinal activity. Uses a light stimulus displayed as hexagons in different sizes over 20–30′ retinal surface. Each hexagon flickers—black‐white. This modality is generally used in experimental set ups—requiring advanced training, expensive equipment, and needs GA for patient.

## Pattern electroretinography (PERG)

The electrodes are placed as for fERG and there is no change in light intensity, but uses alternating high‐contrast checkerboard/gratings—with variation in alternate rate. Requires fixation (humans)—so necessitates anesthesia in our patients (conscious = unreliable). Classically used to evaluate glaucoma damage—RGCs and ON function.

## Visual evoked potentials (VEP)

VEPs represent the electrical activity generated in the visual cortex during light stimulation of the visual pathways. Reliability/reproducibility can be a problem with the technique and anesthesia is required. Largely this is reserved for experimental studies and investigations of visual deficits in humans.

Measures summed electrical potentials in visual cortex in response to visual stimulation. Recorded on scalp (midline)—recording electrode (nuchal crest), reference (forehead), ground electrodes (vertex) Very small amplitudes (~15 uV) expected so susceptible to signal to noise ratio errors. Used in experimental set ups and single channel most practical (multiple channel requires expensive advanced equipment and training but can assess chiasmal and retrochiasmal activity).

## Fluorescein angiography

IV fluorescein is administered and serial retinal photography (blue light illumination, specialist camera) undertaken. There are different phases of fluorescein flow through fundus—retinal vessels, choroidal vessels and optic nerve vessels. Very sensitive indicator of vascular permeability changes. May assist in investigation of optic neuritis and chorioretinitis.

## Optical coherence tomography (OCT)

OCT is a non‐invasive imaging technique that uses light to create high‐resolution cross‐sectional images of tissue. Within ophthalmology it can assist with investigations of retinal and optic disc disease, providing an in vivo and longitudinal high resolution of tissues (comparable to histopathology sections).

## Magnetic Resonance Imaging (MRI) ± CSF sampling

This has been covered elsewhere by (see ‘What Imaging Modality to do for Investigation of Blindness’ by Fraser McConnell in residents day program) but we will briefly consider its application to ophthalmic and retrobulbar disease causes of blindness. Recent advances in MRI (7 T) have afforded fantastic resolution of the ocular structures and optic nerve sheath, as well as orbital structures. Neuromuscular blockade is required to avoid eye saccades and artifacts so is largely reserved to experimental protocols currently.

## SUSTAINABILITY IN VETERINARY PRACTICE

## Ellie West

As of 2024, we have crossed 6 of the 9 planetary boundaries which define a safe operating space for humanity. Climate change is a global medical and veterinary healthcare crisis and threatens our ability to continue providing quality of care to our patients. But how can I change anything significant in my clinical practice? What should I focus on? This presentation will provide a brief overview of the state‐of‐ play in veterinary sustainability and focus on a few key areas in which veterinary professionals can make a significant difference in their daily clinical life. Be prepared to think global, and act local.

## UNDER PRESSURE

## Fraser McConnell

Pressure of fluid and flow are closely related and there is a close relationship between intracranial pressure and blood/CSF/lymph flow. The appearance of flowing liquids on MRI is complex and varies with pulse sequence, flow velocity and direction and pulse sequence options chosen. Recognition of flow artifacts or absence of artifacts can be helpful in detection of vascular pathology eg thrombi. Venous thrombi in particular are easily overlooked and possibly overlooked in veterinary medicine. Blood flow at the capillary level can be estimated using perfusion‐weighted imaging (PWI) which is simple to perform. Alteration in PWI can potentially give additional information in patients with abnormal CSF flow or intracranial pressure. Assessment of increased intracranial pressure is simple in late stages using MRI, for example, brain herniation but in early/mild cases may be challenging and requires GA. Transocular ultrasonography offers the possibility to measure optic nerve sheath diameter without need for sedation/GA and can act as a surrogate marker for intracranial pressure. In the last decade there has been many developments in the understanding of normal CSF flow which challenges the classical theory. The proof of presence of meningeal lymphatics and recognition of the complexity of normal CSF production, resorption and flow has been aided by MRI CSF flow assessment. MRI flow studies in veterinary medicine is at the early stages and may give insights into many challenging pathologies, for example, normal pressure hydrocephalus. MRI assessment of normal lymph flow is challenging whilst described is unlikely to be clinically applicable in most cases.

## OPTIC NEURITIS/NOM

## João Lemos

Inflammatory optic neuropthies in humans constitute a growing subclass of acquired optic neuropathies.

Over the last 2 decades, new antibodies associated with the presence of optic neuropathy have been discovered, including myelin oligodendrocyte glycoprotein (MOG) antibody and aquoporin 4 (AQ4) antibody.

Importantly, new and more efficient treatments have become available, both for multiple slcerosis‐ related optic neuritis and MOG‐ and AQ4‐related optic neuritis.

In this session we will present an update review on the diagnosis, management and prognosis of inflammatory optic neuropathies in humans.

## EYE MOVEMENTS

## João Lemos

The diagnosis of abnormal eye movements constitutes a challenging task for the clinician, as these reflect the dysfunction of several brain networks and their complex interaction.

Indeed, supranuclear, nuclear and/or internuclear disorders might occur simultaneously in the same individual.

In this session we will highlight the most common eye movement disorders encountered in the clinic, using a structured approach, by spanning ocular fixation, pursuit, saccades and vergence disorders.

The investigation and treatment of the above conditions will also be briefly discussed.

## NYSTAGMUS

## João Lemos

Nystagmus is characterized by rhythmic and involuntary eye movement oscillations, usually comprising a slow eye movement (“slow phase”), followed by a corrective fast movement (“fast phase”). This type of nystagmus is classicaly associated with vestibular disorders. Less often, oscillations might comprise only slow eye movements (“pendular oscillations”). Here, a congenital origin is usually the culprit.

Importantly, the direction of the fast phase of nystagmus, along with its accompaning features, including head impulse response, beahviour of eccentric nystagmus, and presence of vertical strabismus are critical steps in evaluating a patient with nystagmus and further differentiate between a peripheral and central vestibular disorder.

In this session we will review the most common phenotypes of nystagmus, both in central and peripheral vestibular disorders. Congenital nystagmus and rare form of nystagmus will also be addressed.

The investigation and treatment of nystagmus will also be briefly discussed.

## VISUAL PATHWAYS

## Elsa Beltran, Ldo Vet, PGDipVet Ed, FHEA, DipECVN, Associate Professor in Veterinary Neurology‐Neurosurgery, Royal Veterinary College, University of London

The visual system is an important part of the neural function in our patients, allowing a wider peripheral vision when compared to humans when both eyes are used. The structures that we will discuss through this lecture will include the pathways for visual perception including retina, the optic nerve, the optic chiasm ad the optic tract, the lateral geniculate nucleus, the optic radiation and the visual cortex. We will also discussed the pathways for visual reflexes (body and ocular reflex, pupillary constriction and pupillary dilation).

## Neurosensory retina and optic nerve

In cats and dogs, the retina is histologically divided into 10 layers (Parry, 1953). Nine layers form the neurosensory retina (embryonic derivative of the diencephalon, neuroectoderm) and the tenth and most external layer (on the scleral surface and closest to the choroid) is the supportive retinal pigmented epithelium. The central area of this tenth layer has no pigment to allow tapetum to show through; the other nine layers of the retina are transparent with the exception of the blood vessels.

The nine identifiable layers of the neurosensory retina, from the outer (scleral) surface to the inner (vitreal) surface, comprise: the photoreceptor layer; the external limiting membrane; the external nuclear layer; the external plexiform layer; the internal nuclear layer; the internal plexiform layer; the retinal ganglion cell layer; the nerve fiber layer (NFL); and the internal limiting membrane. The neurosensory retina layers contain seven types of major cells (six neuronal and one glial): the outer retinal photoreceptors (rods and cones), bipolar neurons, horizontal neurons, amacrine cells, retinal ganglion cells (RGCs) and the Müller cells (glial cells). These cells convert light into electrical impulses, which are sent, via the optic nerve, to the visual cortex (to be transformed into images) and to the brainstem to elicit reflex pathways that coordinate pupil size, head, neck, eyeball movements in response to visual stimuli and synchronize the animal's biological clock.

The ganglion cell layer contains the cell bodies of the RGCs. There is a new subgroup of RGCs identified called melanopsin‐containing RGCs (intrinsically photosensitive RGCs [ipRGCs]) that are also photosensitive with melanopsin as the photosensitive pigment. This subgroup of RGCs can respond to changes in light without the input of the outer photoreceptors (cones and rods) and contribute to the regulation of circadian behavior, seasonal reproductive rhythm and to the PLR.

The area in the retina with the highest number of photoreceptors and RGCs is called the area centralis (Mowat et al., 2008); this is specialized for high resolution with maximal visual acuity, comparable to the human macula.

The NFL is mainly formed by axons of the RGCs, which course on the vitreal surface of the retina to the optic disc (optic nerve head or optic papilla) and this point is the origin of the optic nerve (cranial nerve [CN] II). Myelination starts at different levels across the species, which account for the different shapes, colors and position between cats and dogs.

The optic nerve is a white matter tract formed by RGC axons and glial cells. Optic nerve is a misnomer, as a nerve involves a bundle of axons in the peripheral nervous system (PNS) and is myelinated by Schwann cells; however, the optic nerve is a tract of the central nervous system (CNS) and is myelinated by oligodendrocytes. After the lamina cribrosa, the optic nerve is surrounded by meninges (dura mater, arachnoid membrane and pia mater) with a cerebrospinal fluid (CSF) filled subarachnoid space. The RGC axons in the optic nerve are arranged in a retinotopic manner to maintain the spatial arrangement of the retina. The RGC axons course caudally and enter the skull through the optic canals, located in the presphenoid bone at the level of the rostral cranial fossa, to merge into the optic chiasm. The presphenoid bone in the cat but not in the dog contains a sinus (known as the presphenoid sinus). Lesions at the level of the presphenoid bone (e.g., severe presphenoid sinusitis in a cat) can damage the optic nerve and compromise vision (Beltran et al., 2010; Busse et al., 2009).

## Chiasm and retrochiasmal pathways to the visual cortex

The proportion of decussating axons varies between species and correlates with the degree of binocular vision. Species with more binocular fields of view have a smaller percentage of axons crossing at the level of the optic chiasm. Around 66% and 75% of the RGC axons decussate at the optic chiasm in cats and dogs respectively, and the rest remain ipsilateral (around 34% in cats, around 25% in dogs, around 80%–90% in horses and cows) (Boire et al., 1995; Jacqmot et al., 2020). The axons that decussate come from the RGCs in the medial aspect of the retina (which provides the lateral field of view), while the ipsilateral axons come from the RGCs in the lateral aspect of the retina (which provides the medial field of view). The optic chiasm is located intracranially on the floor of the rostral cranial fossa (presphenoid bone) and rostral to the pituitary gland. After the optic chiasm, the RGC axons continue as the optic tract and course caudal dorsolateral over the side of the thalamus. The majority of the optic tract axons synapse in the lateral geniculate nucleus (LGN), located caudal dorsolateral in the thalamus. Some of the optic tract axons (including melanopsin‐containing RGC axons) leave the optic tract before reaching the LGN to relay information to extracortical nuclei in the brainstem (pretectal nucleus, rostral colliculus, and suprachiasmatic nucleus). The axons from the neuronal cell bodies in the LGN project into the internal capsule and course caudally as the optic radiation to terminate in the visual cortex and produce the visual perception of images (conscious). A recent study identified the Meyer's and Baum's loops in the canine visual pathway (Jacqmot et al., 2020). These loops are axons from optic radiation and therefore contribute to the visual system. The axons of Meyer's loop pass near the temporal lobe to project themselves into the occipital cortex. The axons of Baum's loop make a caudomedial path to project at the level of the parietal cortex before reaching the occipital cortex. This is important neuroanatomy that might need to be considered to prevent damage to the visual system when neurosurgical or radiotherapeutic procedures are planned (Jacqmot et al., 2020).

## Autonomic innervation to the eye

The autonomic innervation to the eye has central and peripheral components, including higher centres in the hypothalamus and midbrain and axons and nuclei in the pons, medulla oblongata and spinal cord. The autonomic system has mainly two components: the general visceral afferent system and the general visceral efferent system with its parasympathetic and sympathetic divisions. The parasympathetic innervation to the eye regulates the iris muscle response (pupil size) to the amount of environmental light, while the sympathetic innervation to the eye regulates the iris muscle response (pupil size) to central factors such as emotion, pain and distress. The iris sphincter muscle is primarily under the control of the parasympathetic nervous system while the iris dilator muscle is primarily under the control of the sympathetic system. Iris muscle constriction (miosis) is produced by contraction of the iris sphincter muscle and relaxation of its antagonist muscle (iris dilator muscle). On the other hand, iris muscle dilatation (mydriasis) is produced by contraction of the iris dilator muscle and relaxation of its antagonist muscle (iris sphincter muscle). This is referred to as reciprocal innervation(Yoshitomi and Ito, 1986). Therefore, pupillary size (under even illumination conditions) is an indicator of the autonomic nervous system tone to the eye.

## Parasympathetic innervation to the eye

The afferent pathways that contribute to the parasympathetic innervation to the eye arise from the retina, where the impulses originate after light stimulation to the photoreceptors (rods, cones and intrinsically photosensitive RGCs [ipRGCs]). These impulses travel within the RGC axons (optic nerve) and reach the optic chiasm. The majority of the RGC axons decussate at the level of the optic chiasm (around 66% in cats and around 75% in dogs) and continue as part of the optic tract; the rest of the axons remain ipsilateral. Some of the optic tract RGC axons (around 20%) bypass the LGN and course caudally to synapse in the pretectal nucleus (PN) (de Lahunta et al., 2021).

The PN is located rostrally in the midbrain tectum and contributes to the PLR pathway. From the contralateral PN, the majority of the axons (around 66% in cats and around 75% in dogs) cross over again through the caudal commissure and reach the parasympathetic nucleus of the oculomotor nerve (ipsilateral side to the eye where the light stimulus is given). The parasympathetic oculomotor nucleus (preganglionic nucleus, known as Edinger Westphal nucleus in human neuroanatomy) is located in the rostral part of the midbrain and very close to the midline. The remaining axons from the PN (around 34% in cats and around 25% in dogs) remain ipsilateral to the PN, reaching the contralateral parasympathetic nucleus of the oculomotor nerve to the eye where the light stimulus is given.

The efferent parasympathetic axons (preganglionic fibers) from the parasympathetic oculomotor nucleus travel with the motor fibers of the oculomotor nerve, coursing ventrally and emerging on the medial side of the crus cerebri. The parasympathetic axons are located medially to the motor fibers of the oculomotor nerve on the floor of the middle cranial fossa and therefore they are the first to be affected when a structural lesion (such as a pituitary gland mass) arises and extends laterally from the midline.

The parasympathetic axons leave the cranial cavity through the orbital fissure and synapse in the ciliary ganglion (postganglionic neuron) caudal and lateral to the eyeball. These postganglionic parasympathetic axons (five to eight short ciliary nerves in dogs; two short ciliary nerves in cats, nasal (medial) and malar (lateral) nerves) innervate the ciliary body and the iridial sphincter pupillae muscle of the iris to control ocular accommodation and pupil constriction and also give reciprocal cholinergic inhibition to the iridal dilator muscle, causing iridal sphincter contraction and dilator muscle relaxation (pupillary constriction). The reflex in the illuminated eye is considered as the direct PLR, whereas the reflex in the contralateral eye is the indirect, or consensual, PLR.

## Sympathetic innervation to the eye

The sympathetic innervation to the eye is described as a three‐order neuron pathway. The cell bodies of the first order neurons are in the caudal nuclei of the hypothalamus, which are activated by emotional factors or noxious stimuli. These first order neurons project caudally and ipsilaterally via the lateral tectotegmental spinal tract (located in the brainstem and deep in the lateral funiculus of the spinal cord) to the preganglionic cell bodies (second order neurons), which are in the lateral gray column at the level of T1 to T3 spinal cord segments. The axons from the preganglionic neurons join the ventral roots of the segmental spinal nerves at the same level, emerge through the intervertebral foramina and leave the spinal nerves in the segmental ramus communicans to join the thoracic sympathetic trunk. The preganglionic axons continue cranially as part of the cervical vagosympathetic trunk. This sympathetic trunk is associated with the vagus nerve (CN X) and located in the carotid sheath. At the level of the cranial cervical area and caudomedial to the tympanic bulla, the preganglionic fibers terminate in the cranial cervical ganglion (CCG) where they synapse with the postganglionic neurons (third order neurons). The exact route of these postganglionic axons to reach the smooth muscles of the iris remains undefined. One of the recent reported possible routes describes that the postganglionic axons leave the CCG and course cranially through the tympano‐occipital fissure to enter the cranial cavity joining the ophthalmic branch of CN V, coursing on the floor of the middle cranial fossa and emerging through the orbital fissure. The postganglionic sympathetic fibers innervate the smooth muscles of the periorbita, superior and inferior (Müller's) tarsal muscles of the eyelid and the dilator muscles of the iris. The sympathetic input to the dilator muscle causes contraction of this muscle and therefore mydriasis. As previously described in the parasympathetic innervation (see above), the sympathetic innervation also causes a reciprocal inhibition of the other antagonist muscle (iris dilator muscle) and therefore further relaxation of the iris sphincter muscle.

## Vision

The pathways of visual perception previously described, and a lesion at any level can cause visual deficits. The clinical assessment of the visual system (in dim and bright light conditions) is mainly performed by observing the animal moving in an unfamiliar environment and negotiating an obstacle course (maze test), and by assessing the menace response. Unilateral visual deficits may be difficult to detect and requires blindfolding each eye in turn.

The menace response is elicited by making a threatening gesture to the eye involving the visual fields (medial and lateral) and observing closure of the eyelids. In cats, the most reliable examination mode for the menace response was achieved standing behind the cat (Quitt et al., 2019). It is important to avoid touching the eye/eyelashes or creating excessive air currents as this can trigger the palpebral and/or corneal reflex and therefore a false positive menace response. The menace response should also be undertaken in both the medial and the lateral visual fields, when possible, as depending on where the lesion is located, specific types of deficits affecting the visual fields might be present. The menace response requires an intact sensory pathway as previously described (optic nerve, optic chiasm, contralateral optic tract, contralateral LGN, contralateral optic radiation and contralateral visual cortex) and an intact motor pathway to elicit the expected response (closure of the eyelids).

From the visual cortex (mainly from the contralateral occipital cortex) the impulses are transmitted by association fibers to the primary motor cortex (frontal cortex), where the motor pathway of the menace response begins. This pathway has not yet been fully described. The axons from the motor cortex reach the pontine nucleus via projection fibers within the crus cerebri and the longitudinal fibers of the pons. The axons from the pontine nucleus decussate by the transverse fibers of the pons and enter the cerebellum via the middle cerebellar peduncle, reaching the cerebellar cortex, which is ipsilateral to the eye where the menace response is elicited.

The cerebellum then coordinates this response by efferent cerebellar pathways that activate the facial nuclei in the ventrolateral part of the rostral medulla oblongata. A recent study has demonstrated, by transsynaptic tracing in mice, that Purkinje cells in the cerebellar cortex project to the cerebellar interpositus nucleus (CIN), which sends projection fibers to the red nuclei in the midbrain (mainly to the red nucleus contralateral to the eye tested) and the red nuclei send projections to the facial nucleus (mainly ipsilateral to the eye tested) in the medulla oblongata. The axons emerge from the medulla oblongata and leave the cranial cavity via the internal acoustic meatus.

At the base of the internal acoustic meatus, the facial nerve continues laterally through the facial canal of the petrous temporal bone and then curves caudoventrally in the caudal wall of the tympanum to exit the skull through the stylomastoid foramen. The facial nerve (CN VII) innervates the orbicularis oculi muscle eliciting a blink (menace response) (de Lahunta et al., 2021). If the menace response is decreased or absent, the facial nerve needs to be evaluated with the palpebral reflex because facial nerve paresis/paralysis may result in a reduced/absent menace response without involving deficits of visual perception. Cerebellar lesions, particularly lesions affecting the interpositus and lateral cerebellar nuclei, can also result in a lack of ipsilateral menace response without involving a deficit of visual perception; however, other clinical signs of cerebellar dysfunction will also be present. It is important therefore, to understand the difference between vision and the menace response. A dog or a cat can be visual with an absent menace response if there is a dysfunction of the cerebellum and/or facial nerve; however, the menace response is also absent if the cat or dog has absent vision.

The menace response is a learned response and therefore is usually absent during the first 10–12 weeks of age in cats and dogs and the first 2 weeks in horses and cows. It is important to remember that the menace response is a cortically mediated response, which needs to be consciously perceived; therefore, animals that have a decreased level of consciousness, are stressed, lethargic or disorientated may have an abnormal menace response without necessarily having a lesion in the menace response pathway.

## Pupillary light reflex

The pathways of the PLR, and a lesion at any level of these pathways can cause PLR deficits. The PLR is a subcortical reflex that regulates the pupil size in response to the intensity of light that falls on the retina. This reflex assists in adaptation to various levels of darkness/brightness and is driven by the activation of the photoreceptor rods, cones and melanopsin‐containing RCGs, with different degrees of contribution. Qualitative PLR is evaluated by shining a bright light into the eye and assessing the ipsilateral (direct PLR) and the contralateral (indirect or consensual PLR) pupillary constriction. The PLR is present as soon as the eyes are open; however, the PLR may be sluggish until the normal retinal structure has developed (from 6 to 10 weeks old in cats and dogs). The assessment of the consensual PLR is not necessary if the menace response and the direct PLRs are present in both eyes. However, the consensual PLR can be of a great value when assessing the afferent pathways (optic nerve and optic chiasm) in an eye where the posterior segment cannot be visualized, resulting in a direct PLR that cannot then be assessed (such as with severe corneal oedema). A recording system and protocol has been developed in dogs to reliably quantify the PLR; however, further studies are needed to evaluate if quantitative PLR abnormalities can be associated with specific diseases(Whiting et al., 2013; Kim et al., 2015). Stressful environments and noxious stimulation can result in pupil dilatation (with little effect on the PLR) due to the influence of the locus coeruleus on pupillary control via sympathetic activation and parasympathetic inhibition. The locus coeruleus is a nucleus located at the level of the pons and is involved with physiological and psychological responses to stress and pain. Other factors that may cause the PLR to be reduced/absent include a low intensity light source, iris atrophy, posterior synechiae, and prior topical administration of a mydriatic agent. Severe retinal, optic nerve, optic chiasm or optic tract lesions are necessary to cause an absent PLR, therefore with only mild retina, optic nerve, optic chiasm or optic tract dysfunction, there is loss of vision, but the PLR could be normal.

## Chromatic/colourimetric pupillary light reflex

Retinal pathology detection may benefit from chromatic PLR (cPLR), which can distinguish between disorders affecting the outer (closer to the scleral surface) photoreceptors (rods and cones) (as seen in sudden acquired retinal degeneration syndrome, SARDS) and the melanopsin‐containing RCG (ipRCG) (Grozdanic et al., 2007; Grozdanic et al., 2013; Yeh et al., 2017; Grozdanic et al., 2021). This test is performed using a cPLR device (such as Melan‐100®; BioMed Vision Technologies, Ames IA, U.S.A.). This cPLR device is based on the principle that ipRCG can be stimulated with strong blue light (wavelength of 420–440 nm [nanometers]) and induce the PLR. A red light (wavelength of 630 nm) stimulates only the photoreceptors to induce the PLR. Optic nerve lesions may have a decreased to absent PLR regardless of the type of light stimulus used for testing. However, if the disease affects only the photoreceptors (such as in SARDS, immune‐mediated retinitis or retinal degeneration), the patient presents with an absent or decreased cPLR using red light and an intact cPLR using blue light. Therefore, cPLR may be a useful method for screening patients that present with loss of vision and PLR with normal mental status, to determine whether further diagnostic tests to evaluate the retina (such as electroretinography (ERG)) should be performed.

## Swinging light (flashing) test

The anatomical pathway of this test follows the same course as the PLR. This test is performed using a bright light into one eye and, after the direct PLR is achieved (pupillary constriction), the light source is quickly pointed to the other eye in which further pupillary constriction is expected. The test is then performed in a reversed and repeated manner (swinging movements). A normal reflex is characterized by both pupils constricting to an equal degree when receiving the light stimulus, with the illuminated eye causing further constriction. A mild pupillary escape can be seen when the illuminated eye dilates slightly after an initial contraction; this is a normal reflex, which represents an adaptation of a stimulated retina mainly when using weak light sources. A positive swinging flashing test is considered if the pupil significantly dilates when the light reaches the eye. This pathological pupillary escape (known in humans as Marcus‐Gunn sign/pupil) indicates an ipsilateral afferent optic pathway dysfunction (retina or optic nerve).

## Dazzle reflex (photic blink reflex)

**T**he dazzle reflex is another subcortical reflex induced by stimulating the eye with a very strong light, which causes an eyelid blink. This reflex is present from birth in puppies and kittens. The afferent arm of this reflex is similar to the PLR; however, the efferent arm is mediated via CN VII. Optic tract axons synapse in the rostral colliculus and then tectonuclear axons synapse in the facial nucleus at the level the medulla oblongata to elicit an eyelid blink. This reflex can be useful when the pupils cannot be visualized to evaluate PLR, such as in patients with severe corneal oedema. However, the exact anatomical pathways have not yet been fully elucidated and therefore this reflex cannot be used on its own to localize subcortical lesions.

## Fundic examination

Fundic examination is an important part of a neuro‐ophthalmic examination, and it should be routinely performed in patients with vision loss, anisocoria, or systemic disease (for instance in systemic hypertension, infectious diseases, storage disease or nutritional deficiencies). The fundus includes all the structures in the posterior portion of the eye globe that can be evaluated with the ophthalmoscope (directly or indirectly). Direct ophthalmoscopy provides an upright evaluation of the fundus; however, it provides a highly magnified small field of view, and it might be a difficult technique to use for general screening of the fundus when compared with the indirect ophthalmoscope. There is also a higher risk for the examiner given the proximity to the patient's head. Indirect ophthalmoscopy provides an inverted evaluation of the fundus with a larger field of view but less magnification. It can be performed using a magnifying lens (20—30D [diopter] lens; the less magnification the greater field of view) and a transilluminator without the necessity to use commercial indirect ophthalmoscopes that are expensive.

Veterinary surgeons should be aware of the variations that exist between species or from breed to breed (based on coat colors, age and eye color) that could contribute to the heterogeneity of the normal appearances of the fundus. For instance, the fundus in young dogs has a bluish color until the maturation of the retina occurs (usually within the first 3–4 months of life) (Brooks et al., 1999). The pupils should be dilated with a sympathomimetic (tropicamide) unless there is any contraindication for its use (such as patients with glaucoma) and the evaluation should take place in a dark/dimly lit room. The structures that routinely need to be evaluated during a neuro‐ophthalmic examination of the fundus include:

- Tapetal area—this contains reflective material (zinc/cysteine) and the non‐pigmented epithelial layer, it comprises around 33% of the fundus and it is seen as bright yellow, orange, reddish and green colored triangular area in the dorsal aspect. Small black or brown spots may be seen at the peripheral tapetum, which could be due to thinning of the tapetum and visualization of the choroidal pigment. This area should be evaluated mainly for changes in reflectivity and pigmentation. For instance, some inflammatory or degenerative diseases or nutritional deficiencies might show hyperpigmentation.
- Non‐tapetal area this comprises the largest area of the fundus peripherally and ventrally, with dark brown or gray color due to the retinal pigmented epithelial cells and underlying choroid
- Retinal blood vessels (venules and arterioles)—there are some variations in the pattern between species and breeds. It is important to evaluate the size of the vasculature and/or the presence of hemorrhages (i.e., hypertensive retinopathy, chorioretinitis).
- Optic nerve head (papilla)—this represents the axons of the RGC entering the scleral lamina cribrosa where myelination starts. In dogs, myelin extends rostral to the scleral lamina cribrosa and covers the surface of the optic nerve head beyond the scleral canal, giving a circular, triangular or even an irregular shape. In cats the optic nerve head is small, circular and it lacks myelin as the myelination starts at the level of the lamina cribrosa and continues caudally (Brooks et al., 1999). The optic nerve head can show some pathological changes including:
- Papillitis (inflammation of the optic nerve head)—this can be seen as an oedematous, raised optic nerve head with swollen hyperaemic margins, which can obscure overlying blood vessels with or without peripapillary hemorrhage.
- Papilloedema—this represents oedema of the optic nerve head due to raised intracranial pressure or optic neuropathy.
- Optic nerve head hypoplasia—this results in a small optic nerve head with absent/reduced vision likely due to a failure of RGC development.
- Optic nerve head atrophy—this appears as a flat or cupped optic nerve head with gray color and attenuation of the blood vessels, which represents an irreversible damage to the RGC, with different possible underlying causes, (e.g., inflammation, ischaemia, chronic compression [for example from chronic glaucoma]).

Retinal detachment can also be diagnosed with fundoscopy or, in severe cases, by shining a light into the patient's eye and observing a veil of tissue posterior to the lens. Retinal detachment is a clinical sign with several possible underlying causes, including systemic hypertension, neoplasia, inflammation, infectious disease or even congenital abnormalities.

## Electrophysiological evaluation of the visual system

Electrophysiological evaluation of the visual system largely comprises electroretinography and visual evoked potentials (VEPs, also called visual evoked responses). It still has a valued role in the era of advanced imaging (magnetic resonance imaging (MRI)/computed tomography (CT)) in both clinical and research neuro‐ophthalmology.

## Electroretinography

Electroretinography (ERG) evaluates retinal function and assesses the retinal cellular responses to a light stimulus. It is useful for the identification of vision loss due to retinal disease including SARDS, or progressive retinal atrophy (Pasmanter and Petersen‐Jones, 2020). ERG can be performed under general anesthesia, or under sedation in a cooperative patient, and it requires the time of dark pupil adaptation (patient placed in a dark room to allow retina to become maximally sensitive to light) as this can affect the ERG results (Lee et al., 2009). Conventional ERG recording uses a corneal contact lens electrode, a skin reference and a ground electrode to record retinal voltage changes that occur in response to a defined flash, or repeated flashes, of light. The response is expressed as a waveform, with a‐ and b‐waves being the most commonly recorded. The waveform and the amplitude and latency of the a‐ and b‐waves are measured. The amplitude of the a‐wave and the b‐wave increase with the strength of the light stimulus strength, which can be used to evaluate retinal sensitivity(Pasmanter and Petersen‐Jones, 2020). The a‐ and b‐waves are the primary ERG components used for assessing retinal function using conventional ERG. However, other waveforms are recognized and used to evaluate retinal function (Pasmanter and Petersen‐Jones, 2020).

## Visual evoked potentials

VEPs are recordings which arise in response to brief flashes of light. VEPs are recorded using electrodes attached to the scalp and signal averaging techniques. The resulting waveform can be used to assess the function of the central retinal region and post‐retinal structures, including the optic nerve, optic chiasm, optic tracts, lateral geniculate nucleus, optic radiation and visual cortex. Obtaining VEPs is largely a research procedure and its use in clinical neuro‐ophthalmology is limited, but in generalized CNS disorders the VEP may be used to infer white matter conduction velocity within the CNS by determining conduction velocity within the optic nerve (Maehara et al., 2018a; Maehara et al., 2018).

## Imaging

Cross‐sectional imaging can provide an excellent complementary diagnostic modality to investigate the dysfunction of the neuro‐ophthalmological structures in cats and dogs.

## Orbital ultrasonography

Optic nerve sheath diameter ultrasonography (ONSD‐US) is used in human critical care units to assess intracranial pressure (ICP) and to monitor patients during hospitalization that could develop raised ICP (Koziarz et al., 2019). Recent studies in dogs have shown the feasibility of performing this technique and that the measurement of the maximum ONSD‐US may provide a noninvasively monitoring tool for evaluation of ICP (Ilie et al., 2015; Smith et al., 2018). Clinical research is required to further evaluate this technique.

Other visual structures accessible by ultrasound include the extraocular muscles (Penninck et al., 2001), which could contribute to the diagnosis of extraocular myopathies in dogs (for instance, extraocular myositis) (Allgoewer et al., 2000; Williams, 2008).

## Computed tomography

CT allows detailed information of the bones of the skull, including the orbit, and sphenoid bones (presphenoid and basisphenoid) to be visualized. This is especially useful in cases with traumatic brain injury affecting the visual pathways.

## Magnetic resonance imaging

MRI allows investigation of the possible relationship between clinical signs and structural lesions along neuro‐ophthalmological pathways. However, prior to interpretation of MRI structural abnormalities, it is important that the MRI appearance of the normal anatomy of the visual pathways and the surrounding structures are well known.

## Cerebrospinal fluid

CSF analysis could be of diagnostic utility in cases of meningoencephalitis; however, it is unclear of its value in cases of isolated optic neuropathy. The CSF dynamics between the optic nerve subarachnoid space and the CSF is not fully understood. It is possible that there is a free flow of CSF in the subarachnoid space of the optic nerve, creating an optic nerve compartment syndrome, limiting the value of CSF analysis in these patients (Hao et al., 2020).

## OP1

### Tremors in cats: 105 cases (2004–2023)

#### ETheofanis Liatis^1^; Sofie F. M. Bhatti^2^; Steven De Decker^1^

**Keyword**: twitches, feline, FIP, PSS, cerebellar abiotrophy.

##### ^1^Department of Clinical Science and Services, Royal Veterinary College, University of London, Hatfield, UK; ^2^Small Animal Department, Small Animal Teaching Hospital, Faculty of Veterinary Medicine, Ghent University, Merelbeke, Belgium

**Introduction/purpose:** Although tremors are common neurological presentations, there is little known about their clinical features and underlying aetiologies in cats. The aim of this study is to evaluate the clinical features and underlying diagnoses in cats with tremors.

**Methods:** Retrospective, single‐center, study of cats with tremors between 2004‐2023. Inclusion criteria included complete medical records, presence or report of the tremor, and a final or presumptive diagnosis.

**Results:** One hundred five cats met the inclusion criteria. The most common diagnoses associated with tremors were degenerative encephalopathy (19/105; 18.1%), feline infectious peritonitis (FIP) (17/105; 16.2%), congenital portosystemic shunt (17/105; 16.2%), intoxication (16/105 15.2%) and polyneuropathy (8/105; 7.6%). Most common degenerative encephalopathies were suspected cerebellar cortical degeneration (7/19; 50%) and lysosomal storage diseases (7/19; 50%) and manifested intention head tremors. Intention head tremors were also seen in cats with FIP and thiamine deficiency encephalopathy. Portosystemic shunt tremors could be focal or generalized, intentional, nonintentional or both. The most common intoxication was permethrin ingestion and was most commonly associated with generalized tremors without accompanying neurological signs. The most common type of tremor in cats with a polyneuropathy was a generalized or limb tremor.

**Discussion/conclusion:** Different tremor phenotypes occur and are associated with specific underlying diagnoses in cats. This information can aid clinicians on the best way to approach cats with a variety of tremor phenotypes.

## OP2

### Polymyositis in the dutch kooiker dog

#### Paul Mandigers^1^; Yvet Opmeer^1^; Vanessa Alf^2^; G. Diane Shelton^3^; Frank Van Steenbeek^1^; Claudia Rozendom^1^; Peter Van Kooten^4^; Victor Rutten^4^; Guy Grinwis^5^; Marjo Hytönen^6^; Marco Rosati^2^; Hille Fieten^1^; Hannes Lohi^6^; Peter Leegwater^1^; Kaspar Matiasek^2^

**Keyword:** Myopathy, Genetic disease, Immunohistochemistry, Dysphagia.

##### ^1^Expertise Centre of Genetics, Department of Clinical Sciences, Faculty of Veterinary Medicine, Utrecht University, Yalelaan 108, 3584 CM Utrecht, The Netherlands; ^2^Section of Clinical and Comparative Neuropathology, Centre for Clinical Veterinary Medicine, Ludwig‐Maximilians‐Universität Munich, 80 539 Munich, Germany; ^3^Department of Pathology, School of Medicine, University of California, San Diego; ^4^Section Immunology, Div Infectious Diseases and Immunology, Department Biomolecular Health Sciences, Faculty of Veterinary Medicine, Utrecht University, Utrecht, The Netherlands; ^5^Department of Biomolecular Health Sciences, Faculty of Veterinary Medicine, Utrecht University, Utrecht, The Netherlands; ^6^Department of Veterinary Biosciences, Department of Medical and Clinical Genetics, University of Helsinki and Folkhälsan Research Center, Helsinki, Finland

**Introduction:** In the Dutch Kooiker dog, an inflammatory myopathy exists that presents itself with clinical signs of dysphagia, locomotion problems, or a combination of both. The outcome of this disease is fatal since most affected dogs died within 2 years after diagnosis.

**Method:** We identified over 170 affected Kooiker dogs, originating from various countries, of which the clinical signs, histopathology, immunohistochemistry were inventoried. To unravel the possible genetics behind this disease a genome wide association study (GWAS), next generation DNA sequencing (NGS) and an expression study was performed.

**Results:** Histopathological revealed an inflammatory, primarily lymphohistiocytic polymyositis. Immunohistochemistry showed that CD3+ T‐cells were the predominant inflammatory cell type, accompanied by CD8+ cytotoxic T‐cells, MHC‐I class positive staining on muscle sarcolemma, MHC‐II class molecules expression on myofibers. A GWAS of 33 cases and 106 controls that indicated the involvement of a region on chromosome 19 containing the cytokine gene pair IL21 and IL2 (praw = 2.1×10^−14^). A NGS revealed that 8 cases shared a 39 kb deletion, situated 10 kb upstream of IL21. Leukocytes of affected, untreated dogs that were homozygous for the deletion overexpress IL21 and IL2 after stimulation with mitogens.

**Discussion:** These findings suggest that elements located 10–49 kb upstream of the IL21/IL2 gene pair play an important role in the regulation of the canine genes and that deletion of these elements is a risk factor for polymyositis in Kooiker dogs and can be used to eradicate this disease.

## OP3

### Comparative study of intranasal diazepam and midazolam as rescue treatment in dogs with epileptic seizures

#### Jose Luis Caldera^1^; Jose Ignacio Cristóbal^1^; Ángela Durán^1^; Paloma Nicolás^1^; Javier Duque^1^; Alba Díaz^1^; Irene Cantalejo^1^; Inmaculada Sevidane^1^

**Keyword:** Epileptic seizures, midazolam, diazepam, intranasal.

##### ^1^Veterinary Teaching Hospital, Animal Medicine Department, University of Extremadura, 10 003 Cáceres, Spain

**Introduction/Purpose:** Epilepsy is a neurological condition in dogs characterized by seizures resulting from abnormal electrical activity in the central nervous system. During a seizure, it's important to use rescue medications, with diazepam and midazolam being commonly chosen. The objective of this study was to compare the efficacy of these drugs (diazepam and midazolam) administered intranasally in the treatment of patients experimenting seizures, based on cessation and recurrence times.

**Methods:** This study involved 14 patients who experienced epileptic seizures, divided into two groups: 7 dogs in the midazolam group (at a dose of 0.2 mg/kg) and 7 animals in the diazepam group (at a dose of 0.5 mg/kg). The patients received the drug intranasally, and cessation times were recorded, representing the interval between drug administration and the end of seizures. Subsequently, recurrence times were recorded, indicating the interval between the end of one seizure and the beginning of the next.

**Results:** The intranasal treatment effectively stopped seizures in all patients. The midazolam group exhibited an average cessation time of 82.57 s, while the diazepam group was 35 s. Relapse occurred in 85.7% of midazolam‐treated animals, with an average relapse time of 270.17 min. Only one animal treated with diazepam experienced relapse after 1080 min.

**Discussion/Conclusion:** In this study, intranasal rescue treatment has shown its effectiveness in patients with epileptic seizures. Animals treated with intranasal diazepam experienced a significant reduction in seizure cessation time compared to those treated with midazolam, along with a lower recurrence rate. Although both drugs were effective in stopping seizures.

## OP4

### Ultra‐long‐term electroencephalographic monitoring device implantation in dogs

#### Casey Rogers^1^; Nina Meyerhoff^1^; Sebastian Meller^1^; Holger Volk^1^

**Keyword:** EEG, long‐term epilepsy monitoring, continuous EEG, EEG implant.

##### ^1^University of Veterinary Medicine Hannover

**Background:** Implantable EEG recording devices have been used for ultra‐long‐term epilepsy monitoring both in clinical and home settings in people. Objective and accurate seizure detection and recording at home could be of great benefit in diagnosis, management and research in canine idiopathic epilepsy (IE). Continuous EEG monitoring would allow accurate detection of seizure patterns, seizure cycles, and seizure frequency. An EEG acquisition system usable in an “out of clinic” setting could improve owner and veterinary compliance for EEG diagnostics and seizure management. We aimed to investigate whether a subcutaneous ultra‐long term EEG monitoring device designed for humans could be implanted in dogs.

**Methods:** A cadaver study with 8 medium to large breed dogs was conducted. Comparatively using a subcutaneous and submuscular approach to implant the UNEEG SubQ‐Implant in each dog. Positioning was controlled via CT post implantation and cranial measurements were taken.

**Results:** In four of the eight dogs a submuscular implantation without any complications was possible. Complications were close contact to the optic nerve in the first approaches, before the implantation angle was changed and in the smallest dog contact of the implant with the orbital fat body. Cranial measurements of less than 95 mm length proved to be too small for reliable implantation via this approach. The subcutaneous approach showed severe limitations and the implant was prone to dislocation.

**Conclusion:** The UNEEQ SubQ‐Implant can be implanted in dogs, via submuscular approach. CT imaging and cranial measurements should be taken prior to implantation.

## OP5

### Treatment of presumed acute thoracolumbar intervertebral disc herniation with intradiscal chondroitinase ABC

#### Nick Jeffery^1^; Paul Freeman^2^

**Keyword:** Intervertebral; dog; disc; herniation; extrusion; recovery.

##### ^1^Texas A&M University; ^2^University of Cambridge

Decompressive surgery is a reliable therapy for acute thoracolumbar disc extrusion but is prohibitively costly for many owners. Non‐surgical therapy can also be successful but there is little prospectively acquired data to define its value. Here we describe the results of treatment of presumed thoracolumbar extrusion by intradiscal enzyme injection.

We recruited non‐ambulatory dogs <15 kg that presented within 48 h of onset for acute suspected thoracolumbar disc extrusion, and whose owners were unable to afford routine surgery. Lesion site was identified by neurologic examination, notably cutaneous trunci reflex and spinal palpation. Dogs were sedated with dexmedetomidine/ketamine/methadone and 22G spinal needles inserted into 4 consecutive discs around the presumed lesion site under fluoroscopic guidance. Chondroitinase ABC (1.25 U) was injected at each site. Dogs were discharged to the owners with instructions for moderate exercise limitation. Follow‐up was maintained by cell phone or email.

In all, 50 dogs were treated. Of 40 deep pain positive dogs, 38 (95%; 95% CI: 83%–100%) recovered to walk.

50 steps unaided by a median of 11 days. Of the 10 deep pain negative dogs, three developed progressive myelomalacia, three were lost to follow‐up before 3 months and two of the remaining dogs recovered (recovery rate of 2/7 = 29%; 95% CI: 8%–65%). No complications relating to the procedure were identified.

The results provide further evidence that decompressive surgery is not essential for recovery of ambulation in many dogs presenting with acute severe thoracolumbar disc extrusion. Greater precision in recovery proportions requires further data accumulation, especially for those presenting deep pain negative.

## OP6

### The diagnostic yield of whole‐genome sequencing in dogs and cats with suspected neurogenetic disorders

#### Rodrigo Gutierrez Quintana^1^; Julien Guevar^2^; Vidhya Jagannathan^3^; Tosso Leeb^3^

**Keyword:** Canine, Feline, Genetics, Precision Medicine.

##### ^1^University of Glasgow; ^2^Anicura Tierklinik Thun, Switzerland; ^3^Institute of Genetics, Vetsuisse Faculty, University of Bern, Switzerland

One third of known monogenetic conditions in humans cause diseases that affect the nervous system. Thus, it is very likely that canine and feline neurogenetic conditions are more common than previously assumed. Advances in bioinformatics and genomic technologies have enabled the development of whole genome sequencing (WGS) as an affordable diagnostic tool to identify novel pathogenic variants.

Retrospective study of dogs and cats investigated for suspected neurogenetic conditions using WGS. The phenotypes of eligible patients were classified into central nervous system (CNS) disorders, peripheral neuropathies, myopathies, and others. The genetic variants were classified according to the guidelines of the American College of Medical Genetics and Genomics as pathogenic, likely pathogenic and variant of unknown significance (VUS).

A total of 84 patients (71 dogs, 13 cats) underwent WGS. The median age at which WGS was performed was 0.7 years. A pathogenic (19/84) or likely pathogenic (4/84) variant was reported in 27% (23/84) of patients (25% of the dogs and 38% of the cats). Three of the cases were only solved due to additional linkage analysis of family members. A VUS was identified in 5 cases (6%). The highest diagnostic rate was achieved in myopathies (4/9, 44%) and peripheral neuropathies (3/8, 37%). The category “others” had a diagnostic rate of 28% (4/14) and CNS disorders 23% (12/53).

In our cohort, the diagnosis of more than a quarter of patients was reached through WGS. This will support the future integration of WGS for investigating suspected neurogenetic disorders in dogs and cats.

## OP7

### The diagnostic yield of whole‐genome sequencing in dogs and cats with suspected neurogenetic disorders

#### Sophie Wyatt^1^; Stefano Cortellini^1^

**Keyword:** Infarct, Cerebrovascular accident (CVA), Coagulation, Thrombus.

##### ^1^Queen Mother Hospital for Animals, Royal Veterinary College, UK

**Introduction/purpose:** Platelet function has not specifically been evaluated in canine ischaemic stroke and information on coagulation status in dogs with this condition is limited. This prospective study investigated platelet aggregation and viscoelastic monitoring in canine ischaemic stroke.

**Methods:** Dogs diagnosed with ischaemic stroke following advanced imaging of the brain were enrolled. Assessment of coagulation status was performed via multiple electrode impedance aggregometry (MEPA) expressed as area under the curve (AUC) of aggregation units, and viscoelastic coagulation monitor (VCM). Healthy dogs were recruited on 1:1 ratio as a control group. Long‐term (>6 months) follow‐up information was collected.

**Results:** Six affected and six control dogs were recruited. Affected dogs had significantly higher MEPA AUC compared to control dogs in response to collagen (P = 0.04) and ADP (P = 0.02) agonists. There was no significant difference in VCM values between affected and control dogs. At long‐term follow‐up, four dogs (67%) were alive. Two non‐surviving dogs euthanised because of their neurological disease (one 4‐ days post‐diagnosis, and one 115‐days post‐diagnosis following presumed stroke recurrence) had higher MEPA AUC compared to all affected dogs. Only one dog made a complete neurological recovery; this dog had the lowest MEPA AUC of all affected dogs.

**Discussion/conclusion:** Dogs diagnosed with ischaemic stroke had significant increases in MEPA AUC in response to collagen and ADP compared to controls, suggestive of increased platelet aggregation. This may be a risk factor for inappropriate thrombus formation and subsequent ischaemic stroke and could represent a potential key therapeutic target for improving outcome and preventing stroke recurrence.

## OP8

### Single dose pharmacokinetics of brivaracetam in healthy dogs

#### Tom Jukier^1^; Kamoltip Thungrat^2^; Robert Arnold^3^; Chu Zhang^3^; Amanda Gross^4^

**Keyword:** Seizure, Pharmacokinetics, Epilepsy, Dog.

##### ^1^Department of Clinical Sciences, College of Veterinary Medicine, Auburn University; ^2^Department of Anatomy, Physiology & Pharmacology, College of Veterinary Medicine, Auburn University; ^3^Drug Discovery and Development, Harrison College of Pharmacy, Auburn University; ^4^Scott‐Ritchey Research Center, College of Veterinary Medicine, Auburn University

**Introduction/Purpose:** Epilepsy is a common neurological disorder affecting dogs. With a fairly large proportion of patients remaining uncontrolled, utilizing newer medications may lead to improved control. Brivaracetam is a novel anti‐seizure medication and a more potent analog of levetiracetam, a popular antiseizure medication used in dogs. The purpose of this study was to investigate the single‐dose pharmacokinetics of brivaracetam in healthy dogs so that a therapeutic dosing regimen could be designed for clinical use.

**Methods:** Seven healthy dogs from a canine colony were selected for this crossover study. Health status was determined based on a physical examination, complete blood count, and serum biochemistry. A single dose of brivaracetam was administered orally and intravenously, separated by a 4‐week washout period. Plasma samples were collected over 36 h. Brivaracetam quantification was performed using high‐pressure liquid chromatography ‐ tandem with mass spectrometry. Pharmacokinetics were determined using non‐compartmental methods.

**Results:** Median (min‐max) for maximal concentration, time to maximal concentration, elimination half‐life, and area under time‐concentration curve/dose were 2.8 (2.4–3.6) μg/mL, 0.5 (0.5–1.0) h, 2.1 (0.9–2.9‐ 2.7) h, and 0.4 (0.4–0.61) μg*h/mL respectively for the oral route. For the intravenous route, elimination half‐life and are under the time‐concentration curve were 3.1 (1.6–12) h and 0.45 (0.3–0.57) μg*h/mL, respectively. Apparent volume of distribution was 10 L, and clearance was 2.2 L/hr. Oral bioavailability was 92%.

**Discussion/Conclusion:** Although brivaracetam shows near complete absorption, the calculated half‐life suggests this medication may be more suited for emergency situations in dogs.

## OP9

### The effects of foraminotomy and distraction‐stabilization on the volume of the lumbosacral neuroforamen throughout spinal motion

#### Raphael Arz^1^; Brian Park^1^; Sebastian Knell^1^; Frank Steffen^2^; Lucas Adam Smolders^1,3^

**Keyword:** DLSS, Distraction‐stabilization, Foraminotomy, Foraminal volume, Foraminal stenosis.

##### ^1^Tierspital Zürich, Department of Small Animal Surgery, University of Zürich, Zürich, Switzerland; ^2^Tierspital Zürich, Department for Neurology, University of Zürich, Zürich, Switzerland; ^3^ IVC Evidensia/Bessys Kleintierklinik, Regensdorf, Switzerland

**Introduction:** Degenerative lumbosacral stenosis (DLSS) in dogs can involve foraminal stenosis and L7 nerve root compression. Surgical options to expand the L7‐S1 neuroforamen (NF) include foraminotomy and distraction‐stabilization. However, the efficacy of these techniques to enlarge the L7‐S1 NF when subjected to biomechanical loading has not been explored. The objective was to investigate the effect of biomechanical loading on the volume of the L7‐S1 NF in the native spine and after foraminotomy and distraction‐stabilization.

**Methods:** Eight canine spinal specimens were tested in three conditions: 1) native, 2) after unilateral foraminotomy and 3) after distraction‐stabilization of L7‐S1. Spines were subjected to axial compression, flexion/extension, lateral bending and axial rotation. The volume of the L7‐S1 NF was measured using computer tomography for each condition and motion direction. Differences between motion extremes and conditions were analyzed using linear mixed models.

**Results:** Foraminotomy induced a significant increase in volume of the L7‐S1 NF in all loading conditions (+3‐46%; P < 0.01). Distraction‐stabilization resulted in a significant, but smaller increase in foraminal volume, only for the loading conditions axial compression (+15% ± 18; *P* = 0.01), extension (+75% ± 32; *P* < 0.01) and axial rotation (+11% ± 12; *P* = 0.01). The volume of the NF after foraminotomy significantly changed when subjected to flexion (+20% ± 25; *P* < 0.01) and extension (−36% ± 8; *P* < 0.01). In contrast, the NF volume after distraction‐stabilization was not affected by biomechanical loading.

**Discussion/Conclusions:**

Both foraminotomy and distraction‐stabilization induce a significant, but different enlargement L7‐S1 NF subjected to biomechanical loading. Therefore, both procedures may be effective methods to enlarge the L7‐S1 neuroforamen affected by foraminal stenosis.

## OP10

### Effect of a single dose of intramuscular methadone administration on neurological evaluation of dogs affected by intervertebral disc herniation

#### Giorgia Stufano^1^; Massimiliano De Vittor^1^; Delia Franchini^2^; Gianvito Lanave^2^; Barbara Betti^1^; Michela Scuttari^1^; Federica Tirrito^1,3^

**Keyword:** dogs, intervertebral disc herniation, methadone.

##### ^1^ AniCura Istituto Veterinario Novara, Granozzo con Monticello, Novara, Italy; ^2^Department of Veterinary Medicine, University of Bari, Valenzano, Italy; ^3^Studio Veterinario Associato Vet2Vet di Ferri e Porporato, Torino, Italy

Methadone is frequently used as analgesic medication in dogs with intervertebral disc herniation (IVDH).

This prospective, blinded, randomized, placebo‐controlled study aimed to evaluate the effects of methadone on neurological examination of dogs with IVDH.

Dogs with IVDH were included and randomized to either receive an intramuscular injection of methadone (0.2 mg/kg) or placebo (saline solution); the same neurologist, for each dog, performed a neurological examination and assigned a neurological score at admission and 20 min after the injection.

Information regarding signalment, neurolocalisation, type of IVDH and magnetic resonance imaging findings was also collected.

Forty‐three dogs were enrolled: 22 received methadone and 21 placebo.

At admission, the most prevalent neurological scores were 3 and 4 (12/43, 27.9% for each score); 38/43 (88.4%) dogs presented focal spinal pain.

After methadone injection, neurological score remained unchanged in 14 (63.6%) dogs, worsened in 6 (27.3%) and improved in 2 (9.1%). Nineteen (90.4%) dogs injected with placebo maintained the same neurological score, while it improved or worsened in 1 (4.8%) dog, respectively.

Focal spinal pain improved in 6 (27.3%) dogs after methadone injection, while it did not change in 16 (72.7%); in the placebo group, focal spinal pain improved in 2 (9.6%) dogs and remained unchanged in 19 (90.4%).

Factors associated with neurological score changes were methadone administration (*P* = 0.003), body condition score (*P* = 0.003), body weight (*P* = 0.006) and intramedullary hyperintensity (*P* < 0.001).

In conclusion, methadone administration, although rarely, could influence the neurological examination of patients with IVDH, especially in overweight dogs and in dogs with intramedullary hyperintensity.

## OP11

### A comparison of the prevalence and nature of complications in french bulldogs and dachshunds with thoracolumbar or lumbar intervertebral disc extrusion

#### Eleftheria Skovola^1^; Steven De Decker^1^

**Keyword:** hemilaminectomy, complications, hospitalization, cost, French bulldog, dachshund, intervertebral disc extrusion IVDE.

##### ^1^Royal Veterinary College

**Introduction/Purpose:** This study aims to compare the nature and prevalence of complications and their effect on postoperative management between French bulldogs (FBDs) and Dachshunds with thoracolumbar intervertebral disc extrusion (IVDE).

**Methods:** Retrospective, single‐centre study of FBDs and Dachshunds between 2019‐2023. Inclusion criteria: complete medical records and neurological examination consistent with acute, progressive, painful thoracolumbar or lumbar myelopathy. Dogs with a previous history of confirmed IVDE were excluded.

**Results:** Five hundred fifty dogs met the inclusion criteria (335 FBD and 215 Dachshunds). Median age was 45 months‐old for FBD and 64 months‐old for Dachshunds (*P* < 0.001). 211/335 (63%) FBD and 168/215 (78.1%) Dachshunds underwent hemilaminectomy and were included in further statistical analysis. Single‐ space hemilaminectomy was performed in 79/211 (37.4%) FBD and 124/168 (78.1%) Dachshunds; the rest underwent hemilaminectomy over more than one intervertebral disc space (*P* < 0.001). Complications occurred in 106/211 (50.2%) FBD and 22/168 (13.1%) Dachshunds (*P* < 0.001). Most common complications in FBD were gastrointestinal signs (diarrhea n = 57; regurgitation *n* = 37; vomit *n* = 5), brachycephalic obstructive airway syndrome (*n* = 27) and urinary tract infection (*n* = 22). Diarrhea (*n* = 15) was the most common complication in Dachshunds. Median length of hospitalization was 5 days for both breeds. The total cost was significantly higher in FBDs compared to Dachshunds (*P* < 0.001).

**Discussion/Conclusion:** Complications are more common in FBD undergoing hemilaminectomy for thoracolumbar IVDE compared to Dachshunds, with different complications encountered between the two breeds. A higher cost is documented for FBD; the length of hospitalization was similar between breeds. This information can aid clinicians' approach to these cases and client counseling.

## OP12

### miRNA expression profile in the csf of dogs diagnosed with degerative myelopathy

#### Dominik Faissler^1^; Elissa Williams^1^; Dawn Meola^1^; Vicky Yang^1^

**Keyword:** miRNA, degenerstive myelopathy, miR‐484, miR‐99a.

##### ^1^Cummings School of Veterinary Medicine at Tufts University

In mouse ALS models, miRNA has been established as biomarkers for diagnosis of this condition. There is, however, a paucity of information related to miRNA in dogs with degenerative myelopathy (DM). This study analyses the cerebral spinal fluid (CSF)‐associated non‐coding (nc)RNA profile of dogs diagnosed with DM. We hypothesize that DM dogs have a different ncRNA expression profile than dogs with other spine disorders. Immediately after CSF collection from the atlanto‐occipital junction samples were centrifuged. RNA was isolated with Qiagen miRNeasy Serum/Plasma kit followed by NGS libraries creation using QIAseq miRNA library Kit. RNA was sequenced on an Illumina HiSeq 2500 sequencer. Sequence read alignment was performed with Qiagen CLC Genomic Workbench, and bioinformatic analysis was performed using DESeq2.

Eight dogs with a clinical diagnosis of DM and 6 controls were enrolled. The most frequent breed was German Shepherd dogs. The median age at presentation was 11.45 years. Breed, age, weight, and sex were not statistically different between the two groups. Both miR‐484 (*P* = 0.0103,‐fold change = −1.93) and miR‐99a (*P* = 0.0127,‐fold change = −1.5) were decreased in dogs diagnosed with DM compared to control dogs. The downregulation of miR‐484 may promote neuronal apoptosis, while downregulation of miR‐99a has been reported in human patients with cerebral infarctions. Furthermore, upregulation of miR‐99a has been associated with reduced risk of apoptosis and volume of neuronal damage after stroke.

To our knowledge, this is the first report showing miRNA expression in the CSF of DM patients. Further validation is needed in a larger population.

## OP13

### Correlation between clinical improvement and imaging findings post‐ treatment in cats with neurological feline infectious peritonitis treated with antivirals

#### Ines Martinez‐Garcia^1^; Rodrigo Gutierrez‐Quintana^2^; Luciano Espino‐Lopez^6^; Francisco Flores‐Ferrero^5^; Anna Suñol‐Iniesta^4,5^; Koen M. Santifort^3,8^; Alex Kennedy^7^; Daniel Sanchez‐Masian^1^

##### ^1^Veterios Referral Hospital. Madrid, Spain; ^2^Small Animal Hospital, School of Biodiversity, One Health and Veterinary Medicine, University of Glasgow. United Kingdom; ^3^Evidensia Small Animal Hospital Arnhem, Arnhem, The Netherlands; ^4^Hospital Veterinaria del Mar. Barcelona, Spain; ^5^Hospital Clínico Veterinario, Universidad Europea. Madrid, Spain; ^6^Departamento de Anatomía, Producción Animal y Ciencias Clínicas Veterinarias. Universidad de Santiago de Compostela, Spain; ^7^Small Animal Medicine Department, Small Animal Specialist Hospital. NSW, Australia; ^8^Evidensia Small Animal Hospital Hart van Brabant, Waalwijk, The Netherlands

Feline infectious peritonitis (FIP) is the most common infectious disease affecting the central nervous system, caused by feline coronavirus. Magnetic resonance imaging (MRI) and computed tomography (CT) findings commonly include meningeal, ependymal and/or periventricular contrast enhancement; ventriculomegaly; parenchymal lesions, syringomyelia and foramen magnum herniation. Until recently, FIP carried a poor prognosis due to a lack of efficacious treatments; however, the use of antivirals has provided a better prognosis.

Our aim is to investigate the correlation between the clinical improvement and post‐treatment imaging findings in cats with neurological FIP treated with antivirals.

Records from seven referral hospitals were searched to identify cases with suspected neurological FIP and inclusion criteria included: 1) MRI/CT‐scan at presentation, 2) antiviral treatment, 3) MRI/CT‐scan after treatment.

Eight cats met the inclusion criteria. Lesion neurolocalisation included central vestibular (6/8) and multifocal (2/8). MRI at presentation was performed in 7/8 cats and CT in 1/8, and findings included ventriculomegaly (8/8), ependymal and/or periventricular contrast enhancement (8/8), cerebellar herniation (2/8) and caudal transtentorial herniation (1/8). 6/8 cats received antiviral treatment GS‐ 441524 only, 1/8 remdesivir only and 1/8 remdesivir followed by GS‐441524. 5/8 cats showed a complete recovery, 3/8 showed residual deficits (2/3 vestibular ataxia, 1/3 proprioceptive deficits). In none of the cats, post‐treatment MRI/CT showed complete remission of the lesions. 4/8 cats showed more severe ventriculomegaly without contrast enhancement.

Based on these results, there is no clear correlation between the clinical improvement and imaging findings in cats with neurological FIP; although future studies with a larger population are necessary.

## OP14

### Care pathway decision support improves GP vet decision making in canine myelopathy

#### Katie Fox^1^; Paul Freeman^1^

**Keyword:** artificial intelligence, decision support system, canine myelopathy, care pathway.

##### ^1^University of Cambridge

**Introduction:** GP vets are often required to perform clinical reasoning and make decisions in the absence of specialist knowledge and support. Care pathway clinical decision support (DS) uses knowledge engineering to convert expert knowledge into a computer‐interpretable format available at the point of care. Care pathways convert patient‐specific data into clinical decision recommendations presented alongside the clinical rationale, using rule‐based arguments coded into the system.

**Methods:** A care pathway was developed to prioritize differential diagnoses and provide general management recommendations for cases of canine myelopathies, using published literature and expert opinion. A crossover study was then performed comparing answers 35 non‐specialist vets each gave to 24 case‐ based questions with and without the use of the pathway. Cases used for the questions were based on dogs presenting historically to a university referral hospital with myelopathies resulting in paresis/plegia. Each participant answered half the questions without any DS and half the questions with care pathway DS, with the question order randomized each time. Participant answers were then scored against a pre‐agreed expert consensus.

**Results:** Results showed that using the care pathway improved differential diagnoses listing and prioritization by 42.4% (95% CI [37.5, 47.3]), whereas management decisions were improved by 6.9% (95% CI [−2.2, 16.0]).

**Discussion:** Care pathway clinical DS could be a useful tool for GP vets, particularly in supporting their ability to produce reasonable prioritized lists of differential diagnoses. More research is needed to establish whether these tools are safe and reliable to be used more widely in clinical practice.

## OP15

### REM sleep behavior and urinary retention disorder in russian blue cats

#### Kimberley Stee^1^; Mario Van Poucke^2^; Ariel Cohen‐Solal^3^; Leen Van Brantegem^4^; Kaatje Kromhout^5^; Sofie Fm Bhatti^1^; Luc Peelman^2^; Ine Cornelis^1^

**Keyword:** REM sleep behavior disorder, Urinary retention, Russian Blue, Neurodegenerative disease, Genetic variant, cat.

##### ^1^Dept. of Small Animals, Faculty of Veterinary Medicine, Ghent University, Belgium; ^2^Dept. of Veterinary and Biosciences, Faculty of Veterinary Medicine, Ghent University, Belgium; ^3^Anicura Centre d'Imagerie Médicale Vetimacs, Brussels, Belgium; ^4^ Dept. of Pathobiology, Pharmacology and Zoological Medicine, Faculty of Veterinary Medicine, Ghent University, Belgium; ^5^Dept. of Morphology, Imaging, Orthopedics, Rehabilitation and Nutrition, Ghent University, Belgium

REM sleep behavior disorder (RBD) is a disease characterized by the loss of lower motor neuron inhibition responsible for skeletal muscle atonia during REM sleep. It has been reported in humans, dogs and cats, and can be idiopathic or secondary to a neurodegenerative disease.

Four young adult Russian Blue cats from 2 unrelated families were presented for progressively worsening RBD episodes frequently associated with urinary loss. Two of these cats also suffered urinary retention with overflow incontinence between RBD episodes.

Clinical examination revealed a large bladder in 2 cats, and neurological examination a bilateral mydriasis with decreased pupillary light reflexes in 2 cats. Complete blood count and serum biochemistry in 4 cats, and MRI of the brain and CSF analysis in 2 cats were unremarkable. The RBD episodes were treated with clonazepam in 1 cat, which improved the RBD episodes but worsened the cat's urinary loss complains. This cat also developed fecal retention which was managed with lactulose. The urinary retention was treated in both affected cats with myocholine, terazosine and regular manual bladder expression, which improved the urinary retention in 1 cat. An autosomal recessive genetic variant associated with this disease was identified by whole genome sequencing. The protein encoded by this variant‐bearing gene has an important role in cell homeostasis.

RBD and urinary retention disorder in Russian Blue cats is a neurodegenerative disease, causing debilitating signs that are challenging to manage with conservative treatment. A genetic test is now available, which allows diagnosis and prevention of this disease.

## OP16

### Neuro‐ophthalmological findings of hypovitaminosis a in beef cattle: A retrospective study

#### Giulia Cagnotti^1^; Cristina Giordano^1^; Giorgia Di Muro^1^; Sara Ferrini^1^; Chiara Giudice^2^; Giuliano Borriello^1^; Antonio D'angelo^1^

**Keyword:** blindness, central nervous system, vitamin deficiency, nutritional disorder, bovine neurology.

##### ^1^Department of Veterinary Sciences—University of Turin (Italy); ^2^Faculty of Veterinary Medicine—University of Milan (Italy)

**Introduction/Purpose:** Hypovitaminosis A is a significant health concern in cattle herds globally. While the systemic effects of vitamin A deficiency on growth, reproduction, and immune function are well‐documented, its neuro‐ ophthalmological manifestations are equally noteworthy, yet often overlooked.

**Methods:** Retrospective case series study conducted by reviewing databases from January 2016 to January 2024 for cattle diagnosed with hypovitaminosis A.

**Results:** Fifteen Piedmontese cattle were included, with a median age of 5 months (IQR: 2.2–8), and 80% were male. The primary complaint was hypovision/acute blindness, with two animals additionally experiencing epileptic seizures. Clinical examination was unremarkable in all animals. Neuro‐ ophthalmological examinations consistently revealed proprioceptive deficits in all limbs, bilaterally fixed dilated pupils with absent pupillary light reflex, and menace response. Fundic examination in 7 cattle showed varying degrees of optic disc papilledema, vascular engorgement, peripapillary hemorrhages, and pigment disruption in the non‐tapetal region.

Comprehensive hematological and biochemical panels were performed on all patients. Cerebrospinal fluid was collected from the lumbar cistern in 10/15 animals, with 9/10 samples analyzed as normal and 1/10 sample deemed ineligible due to blood contamination. Serum vitamin A concentrations were below the normal range (130–380 mcg/L) in all animals, with a median value of 49 mcg/L (IQR: 49–85.5).

All patients received parenteral and oral vitamin A supplementation. However, only calves without optic disc papilledema regained vision.

**Discussion/Conclusion:** While the systemic effects of vitamin A deficiency may be reversible with appropriate supplementation, neuro‐ophthalmological damage incurred during prolonged deficiency may be irreversible, highlighting the importance of early detection and intervention.

## OP17

### Association of the appearance of CSF on haste MRI with clinical factors and short‐term outcome in french bulldogs presenting with thoracolumbar intervertebral disc extrusion

#### Anastasia Morgan^1^; Nicholas Grapes^1^; Steven De Decker^2^; Robert Brash^1^; Jessica Milne^1^

##### ^1^Davies Veterinary Specialists; ^2^Royal Veterinary College

Thoracolumbar intervertebral disc extrusion occurs commonly in French bulldogs. HASTE sequences, on MRI, allow visual approximation of subarachnoid space attenuation. The aim of this study is to investigate the association between subarachnoid CSF attenuation on HASTE sequences, clinical variables, and short‐term outcome in French bulldogs with TL‐IVDE, which may allow for more informed decision making.

Medical records of French bulldogs, that were presented to the authors' clinics, between September 2021 and December 2023, were collated. MRI studies, that included a HASTE sequence, were blindly reviewed. For each patient, a ratio of the mean length of subarachnoid space attenuation, dorsal and ventral to the spinal cord, relative to the length of the L2 vertebra, was calculated and associated with clinical presentation, Modified Frankel Scores, and outcomes.

A total of 119 French bulldogs met the inclusion criteria. Dogs were included whether they had surgical treatment or not. The median neurological grade at presentation and at 4‐weeks post discharge were 3 and 2, respectively, (range 1–5). The median ratio of CSF attenuation was 6.9 (range 0–18.9). French bulldogs with a more severe neurological grade at presentation demonstrated a significantly greater mean ratio of CSF attenuation (*p* ≤ 0.01). French bulldogs with a greater mean ratio of CSF attenuation were demonstrated to be statistically less likely to have an improved neurological grade 4‐weeks post discharge (*P* = 0.027).

In conclusion, greater CSF attenuation length was associated with a higher Modified Frankel Score on presentation and lower likelihood of improvement at 4‐weeks post discharge.

## OP18

### Hyperthyroidism‐associated paroxysmal dyskinesia in three cats: A novel metabolic encephalopathy

#### Javier Espinosa^1^; Inés Martínez^2^; Irene Espadas^2^; Annette Wessmann^1^; Juan José Minguez^1^; Christoforos Posporis^1^; Patricia Álvarez^1^

**Keyword:** Hyperthyroidism paroxysmal dyskinesia electroencephalography cats.

##### ^1^Neurology and Neurosurgery Service, Pride Veterinary Referrals, IVC Evidensia Group, Derby, United Kingdom; ^2^Neurology and Neurosurgery Service, Unavets Veterios Referral Hospital, Madrid, Spain

**Introduction:** Paroxysmal dyskinesia is a rare condition in cats, reported solely in the Sphynx breed as an idiopathic disorder of suspected genetic origin. Feline hyperthyroidism is a widely recognized disease, yet no direct association to paroxysmal dyskinesia has been documented. In contrast, hyperthyroidism‐ associated paroxysmal dyskinesia is well‐described in human medicine. The purpose of our study was to describe paroxysmal dyskinesia as a novel manifestation of hyperthyroidism in cats.

**Methods:** Cats presenting episodic signs consistent with the clinical phenotype of paroxysmal dyskinesia based on video recording and/or direct observation, and a concurrent diagnosis of hyperthyroidism were included in the study.

**Results:** Three cats presenting with recurrent episodes consistent with paroxysmal dyskinesia were recruited. No other clinical signs were noted in between episodes. Physical and neurological examination were normal in all cases. Brain MRI performed in 2 cats was normal. One of these cats also had a normal CSF analysis and a 2‐hour‐video‐assisted wireless electroencephalography not supportive of epileptic activity. Blood analysis was compatible with hyperthyroidism in all cats and appropriate treatment was initiated. Complete resolution of the episodes was achieved following normalization of the thyroid function on repeated blood analysis.

**Discussion/Conclusion:** To the best of our knowledge, this case series describes a novel metabolic encephalopathy in cats with similarities to a well‐known condition in human patients. Hyperthyroidism should be considered as a differential diagnosis in cats with paroxysmal dyskinesia despite the absence of other suggestive signs. Prognosis appears to be excellent when normal thyroid function is restored.

## OP19

### MRI findings in cats with histologically confirmed or serologically suspected central nervous system toxoplasmosis

#### Emmeline Robinson^1^; Maria Ines De Freitas^1^; Daniela Alder^2^; Ezio Bianchi^3^; Kendall E. Day^4^; Alice Dussaux^5^; Antonio Filippi^6^; Sergio A Gomes^7^; Theofanis Liatis^8^; Lauren Marini^9^; Malte Schwartz^10^; Josh D Warren^11^; Sophie Elizabeth Wyatt^8^; Lorenzo Mari^1^

**Keyword:** Toxoplasma gondii, Feline, Intra‐axial, Protozoan, Serology titres.

##### ^1^Neurology department, The Ralph Veterinary Referral Centre, Marlow, UK.; ^2^Marigin ‐ Centre for Veterinary Medicine, Feusisberg, Switzerland; ^3^Department of Veterinary Science, University of Parma, Parma, Italy; ^4^ BluePearl Specialty + Emergency Pet Hospital, Lewisville, TX, USA; ^5^Cordeliers Hospital Center, Neurology unit, Meaux, France; ^6^Concordia Veterinary Clinic, Portogruaro, Italy; ^7^Dovecote Veterinary Hospital, Castle Donington, UK; ^8^Queen Mother Hospital for Animals, Royal Veterinary College, Hatfield, UK; ^9^Ocean State Veterinary Specialists, East Greenwich, RI, USA; ^10^Summit Veterinary Referral Center, Tacoma, WA, USA; ^11^Triangle Veterinary Referral Hospital, Durham, NC, USA

Toxoplasma gondii is a protozoan with central nervous system (CNS) predilection in cats. Diagnosis is based on histological confirmation or serological suspicion and response to appropriate treatment (i.e., clindamycin/sulphonamides). Positive IgM or 4‐fold‐increasing IgG titre supports active infection; however, not all cats generate IgM response, and IgM/incremental‐IgG might be missed in chronic cases. Literature about MRI findings in cats with CNS toxoplasmosis is limited.

Multi‐centre data from cats diagnosed with CNS toxoplasmosis were retrospectively collected. Inclusion criteria were either: 1) histological confirmation or positive CSF PCR; 2) elevated IgM and response to treatment; 3) elevated IgG, MRI compatible with histologically confirmed cases and response to treatment.

Thirteen cats were included (10‐males, 3‐females; aged 2–11 years). Five histologically confirmed, 1 PCR confirmed, 7 serologically suspected. IgM was positive in 7/13 cases (titres 1:50–1:1600; 2/5 histologically confirmed cases) and IgG in 12/13 cases (titres 1:256–1:12800).

10/13 cats had lesions on MRI (7‐focal, 3‐multifocal). 8 brain lesions (4‐telencephalon, 2‐diencephalon, 1‐ medulla‐oblongata, 1‐cerebellum), and 10 spinal cord lesions (4‐cervical, 4‐thoracic, 2‐lumbar) were detected. All were intra‐axial, well‐defined, round/elongated. Signal compared to gray matter was T2W‐iso(11)‐to‐hyperintense(7), and T1W‐iso(16)‐to‐mildly‐hyperintense(2). All lesions presented marked homogeneous (17/18) or ring‐shaped (1/18) contrast enhancement. Moderate‐to‐severe perilesional oedema was present in all but one case. CSF was normal in 6/7 cases. CSF PCR was positive in 1/3 cases.

Toxoplasmosis should be suspected in any cases with intra‐axial, markedly contrast‐enhancing MRI lesions. Negative IgM or CSF PCR does not exclude the disease. A sub‐population of CNS toxoplasmosis cases with normal MRI may exist.

## OP20

### Polymyositis in the Dutch Kooiker dog

#### Paul Mandigers^1^; Yvet Opmeer^1^; Vanessa Alf^2^; G. Diane Shelton^3^; Frank Van Steenbeek^1^; Claudia Rozendom^1^; Peter Van Kooten^4^; Victor Rutten^4^; Guy Grinwis^5^; Marjo Hytönen^6^; Marco Rosati^2^; Hille Fieten^1^; Hannes Lohi^6^; Peter Leegwater^1^; Kaspar Matiasek^2^

**Keyword:** Myopathy, Genetic disease, Immunohistochemistry, Dysphagia.

##### ^1^Expertise Centre of Genetics, Department of Clinical Sciences, Faculty of Veterinary Medicine, Utrecht University, Yalelaan 108, 3584 CM Utrecht, The Netherlands; ^2^Section of Clinical and Comparative Neuropathology, Centre for Clinical Veterinary Medicine, Ludwig‐Maximilians‐Universität Munich, 80 539 Munich, Germany; ^3^Department of Pathology, School of Medicine, University of California, San Diego; ^4^Section Immunology, Div Infectious Diseases and Immunology, Department Biomolecular Health Sciences, Faculty of Veterinary Medicine, Utrecht University, Utrecht, The Netherlands; ^5^Department of Biomolecular Health Sciences, Faculty of Veterinary Medicine, Utrecht University, Utrecht, The Netherlands; ^6^Department of Veterinary Biosciences, Department of Medical and Clinical Genetics, University of Helsinki and Folkhälsan Research Center, Helsinki, Finland

**Introduction:** In the Dutch Kooiker dog, an inflammatory myopathy exists that presents itself with clinical signs of dysphagia, locomotion problems, or a combination of both. The outcome of this disease is fatal since most affected dogs died within 2 years after diagnosis.

**Method:** We identified over 170 affected Kooiker dogs, originating from various countries, of which the clinical signs, histopathology, immunohistochemistry were inventoried. To unravel the possible genetics behind this disease a genome wide association study (GWAS), next generation DNA sequencing (NGS) and an expression study was performed.

**Results:** Histopathological revealed an inflammatory, primarily lymphohistiocytic polymyositis. Immunohistochemistry showed that CD3+ T‐cells were the predominant inflammatory cell type, accompanied by CD8+ cytotoxic T‐cells, MHC‐I class positive staining on muscle sarcolemma, MHC‐II class molecules expression on myofibers. A GWAS of 33 cases and 106 controls that indicated the involvement of a region on chromosome 19 containing the cytokine gene pair IL21 and IL2 (praw = 2.1 × 10^−14^). A NGS revealed that 8 cases shared a 39 kb deletion, situated 10 kb upstream of IL21. Leukocytes of affected, untreated dogs that were homozygous for the deletion overexpress IL21 and IL2 after stimulation with mitogens.

**Discussion:** These findings suggest that elements located 10–49 kb upstream of the IL21/IL2 gene pair play an important role in the regulation of the canine genes and that deletion of these elements is a risk factor for polymyositis in Kooiker dogs and can be used to eradicate this disease.

## OP22

### Minimally invasive biportal endoscopic foraminotomy of L7‐S1 in dogs: Ex vivo assessment and clinical case report

#### Dimitrios Bekiaridis^1^; Antonio Pozzi^1^; Frank Steffen^2^; Julien Guevar^3^; Lucas Smolders^1^

**Keyword:** Neurosurgery, Foraminotomy, Degenerative lumbosacral stenosis, Biportal endoscopic foraminotomy, Minimally invasive spinal surgery.

##### ^1^Clinic for Small Animal Surgery, Vetsuisse Faculty, Winterthurerstrasse 270, 8075 Zurich, Switzerland; ^2^Clinic for Small Animal Neurology, Vetsuisse Faculty, University of Zurich, Switzerland; ^3^Clinic for Small Animal Surgery, Vetsuisse Faculty, University of Bern, Switzerland

**Introduction/Purpose:** Dorsal foraminotomy (DF) is an established surgical technique for treating lumbosacral foraminal stenosis in dogs. A surgical technique for biportal endoscopic foraminotomy (BEF) of L7‐S1 was developed. The aim was to compare effectiveness, visualization, and safety between BEF and DF and to report the first clinical case treated with BEF.

**Methods:** Ex vivo study: foraminotomy of L7‐S1 was performed bilaterally in 12 spinal specimens by way of 1) open dorsal foraminotomy (DF), 2) BEF with a 3.0 mm arthroscope (BEF‐A) or 3) BEF with a 1.9 mm NanoNeedle scope (BEF‐N; 8 neuroforamina/group). Visualization and iatrogenic damage of the L7 nerve root were assessed intra‐ and post‐operatively. Volumetric enlargement of the lumbosacral neuroforamen (NF) was calculated using CT. Linear mixed models and Chi‐Square tests were used for statistical analysis. Case Report: BEF‐A was applied to treat unilateral L7‐S1 foraminal stenosis in a 4‐ year‐old dog.

**Results:** DF and BEF resulted in a significant enlargement of the NF (+58 ± 33%, *P* < 0.01). BEF‐A resulted in a significantly higher enlargement compared to DF (+76 ± 33% and + 46 ± 23%, respectively, *P* = 0.03). BEF‐A provided improved visualization with significantly less nerve root manipulation compared to DF and BEF‐N (*P* < 0.05). BEF‐A of a client‐owned dog with L7‐S1 foraminal stenosis (Figures 1 and 2) resulted in an immediate resolution of clinical signs. The dog remained symptom‐free and CT/MRI imaging revealed an effective enlargement of the NF 8 months post‐operatively.

**Discussion/Conclusion:** BEF‐A provides superior NF enlargement and nerve root visualization compared to DF. We propose BEF‐A as a novel procedure for treating dogs with lumbosacral foraminal stenosis.

## OP23

### Minimally invasive biportal endoscopic foraminotomy of L7‐S1 in dogs: Ex vivo assessment and clinical case report

#### Dimitrios Bekiaridis^1^; Antonio Pozzi^1^; Frank Steffen^2^; Julien Guevar^3^; Lucas Smolders^1^

**Keyword:** Neurosurgery, Foraminotomy, Degenerative lumbosacral stenosis, Biportal endoscopic foraminotomy, Minimally invasive spinal surgery

##### ^1^Clinic for Small Animal Surgery, Vetsuisse Faculty, Winterthurerstrasse 270, 8075 Zurich, Switzerland; ^2^Clinic for Small Animal Neurology, Vetsuisse Faculty, University of Zurich, Switzerland; ^3^Clinic for Small Animal Surgery, Vetsuisse Faculty, University of Bern, Switzerland

**Introduction/Purpose:**

Dorsal foraminotomy (DF) is an established surgical technique for treating lumbosacral foraminal stenosis in dogs. A surgical technique for biportal endoscopic foraminotomy (BEF) of L7‐S1 was developed. The aim was to compare effectiveness, visualization, and safety between BEF and DF and to report the first clinical case treated with BEF.

**Methods:** Ex vivo study: foraminotomy of L7‐S1 was performed bilaterally in 12 spinal specimens by way of 1) open dorsal foraminotomy (DF), 2) BEF with a 3.0 mm arthroscope (BEF‐A) or 3) BEF with a 1.9 mm NanoNeedle scope (BEF‐N; 8 neuroforamina/group). Visualization and iatrogenic damage of the L7 nerve root were assessed intra‐ and post‐operatively. Volumetric enlargement of the lumbosacral neuroforamen (NF) was calculated using CT. Linear mixed models and Chi‐Square tests were used for statistical analysis. Case Report: BEF‐A was applied to treat unilateral L7‐S1 foraminal stenosis in a 4‐ year‐old dog.

**Results:** DF and BEF resulted in a significant enlargement of the NF (+58 ± 33%, *P* < 0.01). BEF‐A resulted in a significantly higher enlargement compared to DF (+76 ± 33% and +46 ± 23%, respectively, *P* = 0.03). BEF‐A provided improved visualization with significantly less nerve root manipulation compared to DF and BEF‐N (*P* < 0.05). BEF‐A of a client‐owned dog with L7‐S1 foraminal stenosis (Figures 1 and 2) resulted in an immediate resolution of clinical signs. The dog remained symptom‐free and CT/MRI imaging revealed an effective enlargement of the NF 8 months post‐operatively.

**Discussion/Conclusion:** BEF‐A provides superior NF enlargement and nerve root visualization compared to DF. We propose BEF‐A as a novel procedure for treating dogs with lumbosacral foraminal stenosis.

## PO1

### Unreliable clinical test result of chromatic pupillary light reflexes in two dogs with amaurosis secondary to optic pathway disorders—A caveat for clinical application

#### Koen Santifort^1,2^; Marta Plonek^1^; Christiane Görig^1^; Ingrid Kraijer‐Huver^1^

**Keyword:** Colorimetric, Optic pathway disease, Retinopathy.

##### ^1^ IVC Evidensia Small Animal Hospital Arnhem, Arnhem, The Netherlands; ^2^ IVC Evidensia Small Animal Hospital ‘Hart van Brabant’, Waalwijk, The Netherlands

**Introduction:** Chromatic (colorimetric) pupillary light reflexes (cPLR) are reportedly useful in canine ophthalmology to differentiate primary retinal disorders from optic pathway disease causing acute blindness. We report two canine cases with cPLR test results consistent with primary retinal disease that were subsequently diagnosed with optic pathway (CNS) disease.

**Methods:** A 7‐year‐old Dachshund and a 7‐year‐old Weimaraner presented for acute blindness. Both dogs failed obstacle tests, showed absent menace responses, bilateral mydriasis, and absent PLRs in both eyes. Ophthalmological examination was otherwise normal. The cPLRs were tested using a BPI‐50 Precision Illuminator (Retinographics Inc., USA).

**Results:** Both dogs exhibited no PLR with red light (660 nm), and brisk though incomplete pupillary constriction with blue light (465 nm) at 200 kcd/m^2^. Electroretinograms (ERG) of both eyes were interpreted as normal for both dogs. MRI of the brain revealed a left‐sided cystic extra‐axial mass (suspected meningioma) in the rostral cranial fossa in the Dachshund and a lesion affecting the rostral bones of the skull base and optic nerves in the Weimaraner (confirmed lymphoma).

**Discussion:** A positive predictive value of 100% has been reported for the cPLR in dogs for the diagnosis of retinal disease (i.e., all dogs with a cPLR to blue light had retinal disease). We report two dogs with optic pathway disease with present blue light‐cPLR. Reasons may include procedural errors in performing the cPLR and sparing of the fibers and pathways of the intrinsic photoreceptor (melanopsin containing) retinal ganglion cells by the optic pathway disorder in these dogs.

## PO2

### Masticatory muscle changes on magnetic resonance imaging of dogs with neospora caninum compared to meningoencephalitis of unknown origin

#### Jessica Zilli^1^; Kathryn Fleming^1^; Chloe Fischer^2^; Tim Sparks^3^; Tom Harcourt‐Brown^2^; Ed Ives^1^

**Keyword:** masticatory, myositis, MUO, neosporosis, canine, MRI, muscles.

##### ^1^Anderson Moores Veterinary Specialists, Winchester, Hampshire (UK); ^2^Langford Vets Small Animal Referral Hospital, Bristol (UK); ^3^Waltham Petcare Science Institute, Waltham on the Wolds, Leicestershire (UK)

Infectious meningoencephalitides represent an important differential diagnosis for meningoencephalitis of unknown origin (MUO) in dogs. Treatment of the latter requires immunosuppression but laboratory test results for infectious agents may take several days. This study investigated whether the presence of masticatory muscle changes on magnetic resonance imaging (MRI) can be used to distinguish dogs with neosporosis from those with MUO at the time of diagnosis.

Cases diagnosed with neosporosis or MUO at two UK referral centres were retrospectively collected. Clinical data were reviewed, and each MRI study was blindly assessed by a radiologist, neurologist and neurology resident for the presence of masticatory muscle changes by consensus opinion.

Twenty‐two neosporosis cases and 23 MUO cases were enrolled. In the neosporosis group, six dogs (27.2%) had masticatory muscle changes, compared to one dog (4.3%) in the MUO group (*P* = 0.047). All six neosporosis cases had bilateral, multifocal, T2W and FLAIR hyperintense, contrast enhancing muscular changes, with three having concurrent masticatory muscle atrophy. The only MUO case with muscle changes had a mild, focal, unilateral temporal muscle lesion which was only visible in the T1W post‐contrast images. Within the neosporosis group, dogs with masticatory muscle lesions had significantly higher cerebrospinal fluid WBC counts and protein concentrations compared to those without muscle changes.

In conclusion, characteristic bilateral, multifocal masticatory muscle changes should raise the index of suspicion for neosporosis in dogs with an imaging diagnosis of meningoencephalitis and starting early antimicrobial treatment is recommended. However, the absence of masticatory muscle abnormalities does not exclude protozoal infection.

## PO3

### Major complications associated with cerebrospinal fluid collection in dogs: Clinical presentation and imaging characteristics in 10 dogs

#### Cecilia‐Gabriella Danciu^1^; Alana Mccarthy1; Abbe Crawford^1^

**Keyword:** cst tap complications, mortality, canine.

##### ^1^Department of Veterinary Clinical Science and Services, Royal Veterinary College, University of London, Hatfield, UK

**Introduction:** Cerebrospinal fluid (CSF) collection and analysis is a commonly used diagnostic test in dogs with neurological disease, but little has been reported on the rate and nature of associated complications.

**Methods:** Retrospective evaluation of the incidence of major complications arising from CSF collection in a single referral hospital and of potential clinical indictors for such complications.

**Results:** From 6507 CSF collections performed between 1998 and 2023, 10 dogs (0.15%) experienced a major complication. Abnormal mental status was detected in 6 dogs on presentation (5 obtunded, 1 stuporous). The most common neuroanatomical localisation was multifocal brain (4/10). MRI characteristics included effacement of cerebral sulci (7/10), meningeal contrast enhancement (5/10), ventricular compression (4/10) and parenchymal enlargement (4/10). CSF analysis aided in the diagnosis of meningoencephalitis of unknown etiology (3/10), cryptococcosis (1/10) and lymphoma (1/10). One dog had haemorrhagic contamination, two had unremarkable CSF and CSF collection was unsuccessful in two.

All dogs showed a marked deterioration in cardiorespiratory and/or neurological status during or immediately following CSF collection. Eight dogs were euthanised; five due to failure to recover spontaneous ventilation, two due to severe neurological deficits and one following cardiopulmonary arrest. Two dogs survived to hospital discharge but with permanent neurological deficits (vestibular dysfunction or central blindness) unassociated with the presenting disease.

**Discussion/Conclusion:** The incidence of CSF collection‐related complications was low, but the mortality rate was high. Abnormal mental status and evidence of generalized cerebral cortical swelling on MRI were often present in dogs encountering a major complication from CSF collection.

## PO4

### Presence of anti‐TRIM46‐autoantibodies in serum and cerebrospinal fluid from a dog presenting with meningoencephalitis of unknown origin

#### Pernille Lindholm Heidemann^1^; Anna Christine Nilsson^2,3^; Sandra Gaedt Schmidt^3^; Morten Blaabjerg^4^; Hanne Gredal^1^

**Keyword:** Autoimmune encephalitis, TRIM46, meningoencephalitis of unknown origin, CNS inflammation, autoantibodies.

##### ^1^Department of Veterinary Clinical Sciences, Faculty of Health and Medical Sciences, University of Copenhagen, Denmark; ^2^Department of Clinical Research, University of Southern Denmark; ^3^Department of Clinical Immunology, Odense University Hospital, Odense, Denmark; ^4^Department of Neurology, Odense University Hospital, Odense, Denmark

**Introduction:** In humans, autoimmune encephalitides (AE) are a group of inflammatory conditions in which autoantibodies (AABs) targeting CNS neurons are present. AE can be divided into two subgroups based on antigen location; 1) antigens are located extracellularly, and AABs are pathogenic ‐ for this subgroup, prognosis is often good, or 2) antigens are located intracellularly, and cytotoxic T‐cells cause disease, with AABs being present, but not pathogenic. The latter subtype is often paraneoplastic and carries a poor prognosis. AABs with an intracellular neuronal target have not previously been reported in dogs.

**Methods:** A broad investigation for AABs targeting neuronal tissue was performed using CSF and serum from an 8‐year‐old Labrador Retriever (male, entire) diagnosed with meningoencephalitis of unknown origin based on exclusion of infectious diseases, and findings on MRI and CSF‐analysis. Cell‐based (EUROIMMUN, ‘Autoimmune Encephalitis Mosaic 6’ and ‘Anti‐Glutamic Acid Decarboxylase 65 kDa’) and tissue‐based (EUROIMMUN, ‘Mosaic: Cerebellum (Rat)/Hippocampus (Rat)’) indirect immunofluorescence (IIF) assays were used.

**Results:** Cell‐based IIF assays were negative. On tissue‐based IIF assays, fluorescence in the axon initial segment was detected in both serum and CSF, consistent with the pattern seen in humans suffering from anti‐ tripartite motif‐containing 46 (TRIM46)‐encephalitis ‐ an intracellular, often paraneoplastic, AE. A cell‐ based assay (EUROIMMUN, research use only, TRIM46 transfected cells) confirmed the presence of anti‐ TRIM46‐AABs.

**Discussion/Conclusion:** This is the first case identifying an intracellular neuronal target for AABs in dogs. Future studies investigating for AABs targeting CNS in dogs should include TRIM46 as a potential target.

## PO5

### Dorsal atlantoaxial stabilization using a pedicle screw fixation system in two dogs

#### Guillaume Leblond^1^; Natalie West^1^; Rodrigo Gutierrez‐Quintana^2^

**Keyword:** Atlantoaxial instability, Neurosurgery, Pedicle screw, Spinal stabilization, Canine

##### ^1^North Downs Specialist Referrals, Brewer St, Bletchingley, United Kingdom; ^2^Small Animal Hospital, School Biodiversity, One Health and Veterinary Medicine, University of Glasgow, Bearsden, United Kingdom

**Purpose:** To describe the clinical outcome and construct viability of a pedicle screw fixation system for the treatment of canine atlantoaxial instability via a dorsal surgical approach.

**Methods:** Dogs diagnosed with atlantoaxial instability were treated using the invetraTM pedicle screw fixation system. Implant positions were planned into the atlas lateral masses (two screws) and the craniolateral aspect of the axis vertebral body (two screws) using planning software (3D SlicerTM). Clinical history and neurological examinations were used to assess functional outcome, while CT images were obtained to evaluate screw positions and implant viability up to 7 months post‐surgery.

**Results:** Two dogs were treated surgically, with 2.0 mm and 3.5 mm pedicle screws respectively. Screws were connected to one another with 3 mm diameter titanium rods. Screw positions were graded as optimal in 6/8 and suboptimal in 2/8 implants on immediate postoperative CT images. Both dogs recovered uneventfully from surgery and either reached normal or improved neurological function at 7‐month follow‐up. No evidence of implant failure or any other complication was detected on CT images at long‐term follow‐up.

**Discussion:** This report suggests that the invetraTM pedicle screw fixation system may be a viable alternative to current atlantoaxial fixation methods. In the authors' opinion, the size of the implants used in this study would be too large to be used in smaller toy breeds often affected by the condition. However, there are smaller sized pedicle screw fixation systems available on the market which may be appropriate for toy breed dogs.

## PO6

### Near infrared fluorescent image‐guided biopsy of a sciatic nerve tumor in a dog

#### Athina Karpozilou^1^; Filipa Lourinho^1^; Beatriz Moreno Aguado^1^; Diogo Miraldo^1^

**Keyword:** NIRF, ICG, biopsy.

##### ^1^Southern Counties Veterinary Specialists

**Introduction/Purpose:**

Near‐infrared fluorescence (NIRF) imaging is a relatively novel technique that can aid surgeons during intraoperative tumor identification. Here we report the feasibility of NIRF using indocyanine green (ICG) to facilitate biopsy of a focal lesion, consistent with a sciatic nerve tumor in a dog.

**Methods:** A 6‐year‐old male neutered cross‐breed dog presented with a 7‐month history of progressive right pelvic limb lameness/paresis. Neurological examination was consistent with right sciatic nerve neuroanatomical localisation. Magnetic resonance imaging (MRI) revealed focal enlargement and strong contrast enhancement of the right sciatic nerve at the level of its exit from the pelvic canal. Ultrasound‐guided fine needle aspiration (FNA) of the lesion followed, and cytology was consistent with a lymphocytic and macrophagic inflammatory process. Based on the inherent limitations associated with FNA and cytology, near infrared fluorescent image (NIRF)‐guided biopsy of the nerve was performed. Three hours prior to surgery, intravenous ICG was administered at 2 mg/kg.

**Results:** Intraoperative evaluation showed a visible partial fluorescence pattern of the thickened nerve, guiding a successful biopsy of the fluorescent lesion. Following histopathological examination and immunohistochemistry, a diagnosis of undifferentiated low‐grade soft tissue sarcoma was made.

**Discussion/Conclusion:** In human medicine, NIRF with ICG is applicable in a large scope of clinical uses, including guided surgery for peripheral nerve sheath tumor resection. This case report suggests that NIRF is a promising technique for intraoperative nerve tumor detection, although detailed delineation may require modifications in ICG administration protocol.

## PO7

### An ‘alternating smile sign’ in two dogs that may resemble dystonic facial seizures

#### Callum Atkins^1^; Joao Miguel De Frias^1^; Theofanis Liatis^2^; Catherine Stalin^3^; Liam Wilson^1^; Tessa Proctor^1^; Eric Glass^4^

**Keyword:** dystonic seizures, hemifacial spasm, facial myoclonus.

##### ^1^University of Edinburgh; ^2^Royal Veterinary College; ^3^University of Glasgow; ^4^Red Bank Veterinary Hospital, New Jersey, USA

**Introduction:** We describe a novel involuntary movement of the muscles of facial expression exhibited as alternating, asynchronous, bilateral spasms consisting of a rapid clonic contraction followed by a protracted tonic phase, resembling a ‘smile’, in two dogs.

**Case presentations:** A 7‐year‐old, female‐neutered, Olde English Bulldogge was presented with behavioral changes, obtundation and anisocoria accompanied by paroxysmal episodes of involuntary bilateral facial movements that discontinued following intravenous levetiracetam administration. Neuroanatomical localisation implicated the forebrain. Magnetic resonance imaging (MRI) revealed a left‐sided intraaxial lesion which was confirmed as a high‐grade oligodendroglioma on post‐mortem examination.

A 2‐year‐old, male‐neutered, French bulldog was presented with pyrexia, cervical hyperaesthesia, left‐sided menace response deficit, left‐sided head tilt, nystagmus and continuous involuntary bilateral facial movements. Neuroanatomical localisation implicated the left peripheral vestibular system and left facial nerve. MRI identified left otitis‐media/interna and empyema. Cerebrospinal fluid (CSF) analysis revealed neutrophilic pleocytosis and increased protein concentration. Streptococcus pneumoniae was isolated from the CSF. Diagnosed with left‐sided otogenic bacterial meningoencephalitis, the dog was treated with amoxicillin clavulanic‐acid and meloxicam for 8 weeks. At 3‐week follow‐up, the clinical signs had resolved.

**Discussion:** These movements may resemble dystonic seizures similar to faciobrachio‐crural dystonic seizures in humans with LGI‐1 auto‐immune encephalitis. Furthermore, the presence of an intracranial mass in case one may be consistent with facial myoclonus or hemifacial spasms, a non‐epileptic sign previously reported with intracranial masses in dogs.

**Conclusion:** Further collaborative work is required to identify the pathophysiology of this ‘alternative smile sign’, including video recording and investigation of the primary cause.

## PO8

### LAMA‐2 related congenital muscular dystrophy in a cat: Clinical and genetic findings

#### Christopher Skinner^1^; Cleo Schwarz^2^; Rodrigo Gutierrez Quintana^1^; G. Diane Shelton^3^; Angelika Rupp^1^; Tosso Leeb^2^; Adriana Kaczmarska^1^

**Keyword:** congenital muscular dystrophy

##### ^1^University of Glasgow; ^2^University of Bern; ^3^University of California San Diego

Muscular dystrophies are a group of more than 30 genetic diseases that cause progressive weakness and degeneration of the skeletal muscles, with several forms reported in dogs and cats.

We examined a 6‐month‐old male neutered cat with chronic generalized weakness. Clinical examination revealed low head carriage with cervical ventroflexion, ambulatory tetraparesis, generalized muscle atrophy, and restricted range of motion in multiple joints, most notably on carpal flexion. Serum creatine kinase activity was increased at 4584 U/L (reference: < 150). Electromyography revealed abnormal spontaneous muscle activity in all limbs. The patient was euthanised due to disease progression.

Post‐mortem evaluation identified substantial fibrotic changes with robust regeneration in the biceps femoris, triceps, and cranial tibial muscles, and mild, acute degeneration. Additionally, intramuscular nerve branches exhibited mild fibrosis, and the sciatic nerve showed mild endoneurial and perineurial fibrosis and rare myelin ovoids, indicative of degeneration.

Autosomal recessive inheritance was suspected as both parents were unaffected. The whole genome was sequenced, and the data was compared to 96 control cat genomes. A private homozygous variant in the LAMA2 gene encoding the a‐2 subunit of laminin‐2 (XM_011282613.4:c.6876_6876 + 2delGGT) was identified. Immunohistochemistry of muscle cryosections showed an absence of staining for lamininα2 and normal staining for laminin γ1 and collagen VI, confirming a functional effect of the LAMA2variant.

This is the first report of a specific causal variant in LAMA2‐related congenital muscular dystrophy in a cat. Further, these findings expand the spectrum of known muscular dystrophy related genes in cats.

## PO9

### Myelopathy in three cats with cobalamin and folate deficiency

#### Christopher Skinner^1^; Rodrigo Gutierrez Quintana^1^; Anne R. Fraser^2^

**Keyword:** subacute combined degeneration, cobalamin deficiency, folate deficiency, myelopathy

##### ^1^University of Glasgow; ^2^Davies Veterinary Specialists

Subacute combined degeneration (SCD) is a well‐recognized metabolic disorder of the spinal cord in humans, caused by cobalamin and folate deficiency. It is characterized by demyelination, resulting in degeneration of the dorsal and lateral tracts of the spinal cord. Distinct MRI changes have been reported. Patients with SCD require aggressive and rapid treatment to prevent irreversible neurological deficits. Just two cases of cobalamin deficiency associated with neurological deficits have been reported in cats. One associated with encephalopathic signs that resolved with cobalamin supplementation and one with myelopathy that was euthanised.

Here we report the signalment, clinical and MRI findings, as well as the long‐term outcome of two cats with signs of C1‐T2 or T3‐L3 myelopathy and concurrent hypocobalaminaemia, and one cat with T3‐L3 myelopathy and folate deficiency.

Three male neutered cats (Savannah, Ragdoll, domestic shorthair) aged between 2 and 7 years old, presented with progressive ambulatory paresis and proprioceptive ataxia localized to the T3‐L3 spinal cord segments (SCS) in two cats, and C1‐T2 SCS in one cat. The physical examination was unremarkable. MRI changes showed diffuse spinal cord swelling in two cats and areas of intramedullary T2‐weighted hyperintensity in two cats. Hematology, biochemistry, CSF, and infectious disease investigations were unremarkable. All cats had cobalamin and folate supplementation. Long‐ term follow‐up ranging from 3 to 4 months showed residual neurological deficits with partial but incomplete improvement of the clinical signs in all cats.

This case series emphasizes the importance of measuring folate and cobalamin in cats with myelopathy.

## PO11

### Bilirubin encephalopathy in four cats following xenotransfusion

#### Abbe Crawford^1^; Cecilia Danciu^1^; Simon Cook^1^

##### ^1^Royal Veterinary College

**Keyword:** Bilirubin encephalopathy xenotransfusion cat

**Introduction:** Hyperbilirubinaemia can result in bilirubin accumulation in neurons, with subsequent neuronal compromise and encephalopathic clinical signs. To date, bilirubin encephalopathy secondary to hepatic compromise or immune‐mediated haemolytic anemia has been reported in cats and/or dogs in a handful of case reports. In this study we report 4 cats with bilirubin encephalopathy following xenotransfusion.

**Methods**: Retrospective case series from a single referral hospital.

**Results:** Four cats (3 DSH, 1 Bengal) with a median age of 11 years (range 8‐14) received a xenotransfusion to manage anemia of various causes. A median of 4 days (range 2‐6) post‐xenotransfusion a marked hyperbilirubinaemia (median 435 umol/l, range 380‐448, reference interval: 0.1‐5) was documented in all cats, as well as new onset severe obtundation and neurological deficits consistent with bilateral vestibulocerebellar dysfunction. Electroencephalography performed in 2 cats showed variable background activity (5–15 Hz, 1–50 uV) with no epileptiform discharges detected. The median duration of hospitalization was 5.5 days (range 5–9) during which all cats showed a progressive decrease in serum bilirubin concentration alongside improving neurological status. At hospital discharge persistent vestibulocerebellar ataxia was present in all 4 cats, and mild obtundation was noted in one. One cat was euthanised 5 days after hospital discharge due to a diagnosis of gastric lymphoma, the remaining three showed continued improvements. Re‐examination 4‐6 weeks later revealed full resolution of neurological deficits in 2 cats, and mild residual pelvic limb ataxia in one.

**Conclusion:** Hyperbilirubinaemia secondary to haemolysis of xenotransfused red blood cells can result in pronounced but transient vestibulocerebellar dysfunction.

## PO12

### Evaluation of blood c reactive protein (crp) and neutrophil‐to‐ lymphocyte ratio (NLR) as a diagnostic tool in canine epilepsy

#### Andreea Despa^1^; Mihai Musteata^1^; Gheorghe Solcan^1^

**Keyword:** C reactive protein, epilepsy, inflammation markers, canine, neutrophil‐to‐lymphocyte ratio.

##### ^1^Faculty of Veterinary Medicine, Ion Ionescu de la Brad Iași University of Life Sciences (IULS), 700 489 Iași, Romania

The involvement of neuroinflammation in epileptogenesis has only recently been investigated. To date, there is a limited number of reports regarding the diagnostic value of blood C reactive protein concentration (CRP) and neutrophil‐to‐lymphocyte ratio (NLR) in epileptic dogs.

We analyzed the CRP and NLR values from 59 epileptic dogs ‐ idiopathic epilepsy with isolated seizures (IEis, *n* = 23), idiopathic epilepsy with cluster seizures (IEcs, *n* = 6), structural epilepsy (SE, n = 20) and reactive epilepsy (RE, *n* = 10). A control group (*n* = 8) was used for NLR comparison.

CRP concentration was abnormal in 10.34% of IE, 50% of SE and 40% of RE. SE dogs had a higher CRP concentration (46.83 ± 13.56 mg/dl) comparing with the IE group (12.42 ± 0.89 mg/dl; *P* = 0.008) and RE one (28.17 ± 13.76 mg/dl; *P* = 0.307). In the IE group, CRP was found to be increased in IEcs (17.41 ± 3.30 mg/L) comparing with IEis, (11.11 ± 0.52 mg/L; *P* = 0.0022). By contrast, NLR values were abnormal in 70% of IEis (8.76 ± 0.66), 100% IEcs (16.02 ± 0.94), 95% of SE (8.19 ± 0.86) and 70% of RE (5.66 ± 1.27). When compared to the control group, both the IE and SE groups displayed increased NLR values (*P* = 0.001, respectively *P* = 0.012).

In conclusion, NLR had a superior accuracy to CRP in the epilepsy diagnostic process, especially in SE and IEcs dogs, and might reflect the neuroinflammation involved in epileptogenesis.

## PO13

### clinical application of 3D‐printed guides with specific cranial contour for brain neoplasia removal in dogs

#### Sergio Moya^1^; Christian Maeso^2^; Nuria Delgado^3^; Juan Carlos Cartagena^4^; Victoria Moya^5^; Sergio Rodenas^1^

**Keyword:** 3D printed skull guides, brain neoplasia.

##### ^1^Neurology Departament Bluecare Partners; ^2^Neurology Departament Ars Veterinaria; ^3^Neurología Departament Bluecare Partners; ^4^Second vets; ^5^Dr. Hospital Veterinary clinic

The application of 3D‐printed surgical guides in veterinary neurosurgery has brought a pioneering change in the last years. The main objectives of this case series were to determine the feasibility and accuracy of these guides in the intracranial neoplasia localisation and removal in dogs, as well as, to evaluate their potential to improve surgical outcomes and reduce surgical times.

Four dogs with brain neoplasia were included. The masses were extra‐axial (2/4) and intra‐axial (2/4), and were located in temporal cortex (2/4), frontal cortex (1/4) and piriform lobe (1/4). Cranial models were generated from CT scans of the patients. Customized guides were designed using 3D modeling software and printed using high‐resolution 3D technology. Simulated procedures were performed on the cranial models to assess the accuracy of the guidewires in localizing the intracranial lesions. Finally, rostrotentorial craniectomy (3/4) and transfrontal craniectomy (1/4) were performed using the 3D‐ printed guides.

Median surgical time was 75 min, which is lower compared to the other intracranial surgeries performed through conventional methods in our institution. Localisation and removal of the mass were considered excellent in all dogs. Moreover, no intraoperative complications were reported.

In conclusion, 3D‐printed patient‐specific cranial guides represent a useful and safe tool for intracranial neoplasia removal in dogs. This innovative approach has the potential to facilitate the localisation of the mass, reduce the risk of perioperative complications, and improve outcomes for patients.

## PO14

### Neuronal ceroid lipofuscinosis (NCL7) in small swiss hounds caused by an intragenic MFSD8 duplication

#### Loderstdt Shenja^1^; Rietmann Stefan^2^; Matiasek Kaspar^3^; Kiefer Ingmar^1^; Leeb Tosso2

##### ^1^Department for Small Animals, Leipzig University, Leipzig, Germany; ^2^Institute of Genetics, Vetsuisse Faculty, University of Bern, Bern, Switzerland; ^3^Section of Clinical & Comparative Neuropathology, Centre for Clinical Veterinary Medicine, Ludwig‐Maximilians‐Universität München, Munich, Germany

**Introduction/Purpose:** Neuronal ceroid lipofuscinosis (NCL) is a heterogenous group of neurodegenerative, lysosomal storage diseases. Here we describe clinical features and genetic analysis of juvenile NCL in an unreported breed.

**Methods:** A Small Swiss Hound with progressive cognitive and motor decline underwent neurological examination, MRI and post mortem examination of the brain. Whole genome sequencing was performed to identify the presumed causative genetic variant.

**Results:** Mentation changes, absent menace response, generalized ataxia and hopping deficits localized multifocal with cerebral and cerebellar involvement. MRI revealed thinned cortical gray matter, prominent sulci cerebri and widened ventricular system. The patient was euthanised and pathological examination showed cerebellar/pallial atrophy and neuronal degeneration with cytoplasmic autofluorescent material consistent with degenerative polioencephalopathy most likely representing a form of NCL. A littermate of the index case reportedly displayed a similar clinical course. Genetic analysis revealed a homozygous duplication of 18 819 base pairs disrupting the reading frame of the MFSD8 gene in both affected dogs, presumably leading to complete deficiency of functional MFSD8 protein.

**Discussion/Conclusion:** The genetic analysis allowed to classify the disease as NCL7. Another variant in the MFSD8 gene has been described in Chihuahuas and Chinese crested dogs with NCL7. We now report the presumably second pathogenic variant in the canine MFSD8 gene in Small Swiss Hounds. Identification of the molecular genetic cause will allow DNA testing to confirm the diagnosis in affected patients of this breed and enable the detection of healthy carriers to avoid unintentional carrier x carrier matings and the breeding of affected puppies.

## PO15

### Neuronal ceroid lipofuscinosis (NCL7) in small swiss hounds caused by an intragenic mfsd8 duplication

#### Giorgia Di Muro^1^; Carlotta Tessarolo^2^; Giulia Cagnotti^1^; Alessandra Favole^2^; Sara Ferrini^1^; Ugo Ala^1^; Claudio Bellino^1^; Giuliano Borriello^1^; Claudia Palmitessa^2^; Giulia Iamone^1^; Barbara Iulini^2^; Marzia Pezzolato^2^; Cristiano Corona^2^; Antonio D'angelo^1^

**Keyword:** Biomarker, Neurofilament light chain, Cerebrospinal fluid, Bovine neurology, Central nervous system.

##### ^1^Department of Veterinary Sciences ‐ University of Turin (Italy); ^2^Istituto Zooprofilattico Sperimentale del Piemonte, Liguria e Valle d'Aosta (Italy)

**Introduction/purpose:** Neurofilament light chain (Nf‐L) is used in human neurology as a biomarker for axonal damage, but it remains unstudied in cattle. This study aims to describe cerebrospinal fluid (CSF) Nf‐L levels in healthy cattle and evaluate CSF Nf‐L's diagnostic potential in neurological cattle.

**Methods:** CSF Nf‐L levels were measured in a group of healthy cattle (*n* = 49) and in a group of cattle with nervous system disorders (*n* = 75). Subgroups of neurological animals were formed based on the definitive diagnosis. Data were statically analyzed to highlight differences between diagnostic subgroups.

**Results:** Physiological median CSF Nf‐L levels were 414 pg/mL in calves and 828 pg/mL in adult cattle. Significantly higher CSF Nf‐L levels were observed in neurodegenerative (median Nf‐L = 49 971 pg/mL, p.

= 0.004) and central nervous system (CNS) infectious disorders (age < 2 months: median Nf‐L = 8863 pg/mL, *P* = 0.04; age 2–12 months: median Nf‐L = 17 474 pg/mL, *P* = 0.0001; age 1–6 years: median Nf‐L = 3546 pg/mL, *P* = 0.001) when compared to healthy controls. CSF Nf‐L levels were significantly higher in neurodegenerative (*P* = 0.005) and CNS infectious conditions (*P* = 0.006) compared to CNS anomalies, and in CNS infections (*P* = 0.0001) compared to metabolic/toxic disorders.

**Discussion/conclusion:** Physiological levels of bovine CSF Nf‐L were described for the first time. Elevated CSF Nf‐L levels in affected cattle suggested the presence of neurodegenerative disorders or CNS infections. These findings could enhance the accuracy of ante‐mortem routine diagnostic procedures for neurological conditions in cattle.

## PO16

### Presumptive meningoencephalomyelitis of unknown origin in geriatric dogs

#### Tamara Heredia^1^; Christian Maeso^1^; Elisabet Domínguez^1^; Emili Alcoverro^1^

**Keyword:** Meningoencepgalomyelitis, Geriatric

##### ^1^ AniCura Ars Veterinaria

Meningoencephalomyelitis of unknown origin (MUO) is a group of non‐infectious inflammatory disease of the CNS of dogs, with a high prevalence in young adult dogs. The current increase in the number of geriatric dogs means that MUO may be seen at older ages, so it should be included as a differential diagnosis. In this case series, we present 5 geriatric dogs with a presumptive MUO.

Median age at diagnosis was 13 years. Included cases were classified as geriatric following the classification proposed in latest studies. All dogs were small breeds and 2/5 were Yorkshire Terriers. Three out of 5 dogs presented multifocal neurolocalisation (2/5 intracranial and T3‐L3 spinal cord, and 1/5 intracranial and cervical spinal cord), 1/5 case with L4‐S3 myelopathy signs and in 1/5 with intracranial localisation (thalamus and brainstem).

MRI showed multifocal, T2W hyperintense, T1W isointense and mildly contrast‐enhancing intra‐axial brain lesions in 4/5 cases; and intramedullary lesions in 4/5 cases. CSF revealed lymphocytic pleocytosis in all cases. CSF‐PCR for endemic infectious agents in the geographical area were tested in 4/5 cases, being negative. Comprehensive blood‐work and thoracic and abdominal imaging revealed no alterations. Treatment with glucocorticoids at immunosuppressive doses and cytosine arabinoside was initiated in all dogs. The outcome was favorable in all dogs with no neurological signs, with a median follow‐up of 11 months after diagnosis.

To the authors' knowledge, this is the largest case series of geriatric dogs with presumptive MUO, with a similarly good outcome compared to previous reports.

## PO17

### Evaluation of cyclosporine and prednisolone in comparison to prednisolone alone for the treatment of suspected meningomyelitis of unknown origin in 47 dogs

#### Tania Al Kafaji^1^; Andrea Corda^2^; Ilaria Tartari^1^; Antonella Gallucci^1^

**Keyword:** cyclosporine, meningomyelitis, dogs, prednisolone.

##### ^1^Veterinary Neurological Center “La Fenice”, Selargius, Italy; ^2^Department of Veterinary Medicine, University of Sassari, Italy

The use of cyclosporine for the treatment of meningitis/meningomyelitis of unknown origin (MUO) with only spinal cord (SC) involvement has never been described in dogs. The objective of this retrospective study was to evaluate the potential efficacy of cyclosporine in combination with prednisolone (Group A) in dogs with suspected spinal MUO in comparison to prednisolone alone (Group B). A positive outcome was defined as a resolution of clinical signs for at least 1 year from the time of diagnosis. A negative outcome was defined as lack of improvement or relapse within 1 year. Forty‐seven dogs were included in the study. Twenty‐one dogs were included in Group A. Sixteen dogs (76%) recovered but clinical relapses were observed in three of them (14%). One dog died due to hematological complications before the one‐year follow up. Twelve dogs (57%) showed a positive outcome. Twenty‐six patients were enrolled in Group B. Initial recovery was observed in all of them, but 16 cases (62%) showed clinical relapses. One dog died due to pulmonary complications before the one‐year follow up. Nine dogs (35%) had a positive outcome. Side effects were mostly mild (48% in group A; 31% in group B).

The outcome was not significantly different between the two groups. Group B showed significantly higher recovery rate and frequency of relapses in comparison to Group A.

Cyclosporine could be considered a valid additional treatment in dogs with spinal MUO, potentially leading to a lower recurrence rate than prednisolone alone.

## PO18

### Clinical presentation and MRI findings of cerebellar disease in dogs

#### Luis Villalonga Rodriguez^1,2^; Pablo Amengual‐Batle^1^; Vicente Aige Gil^3^; Rita Gonçalves^4^; Albert Aguilera Padros^5^; Kiterie Me Faller^5^; Christian Maeso Ordas^6^; Anna Suñol Iniesta^7,8^

##### ^1^ ChesterGates Veterinary Specialists; ^2^Hospital Veterinario Puchol; ^3^Facultat de Veterinària, Universitat Autònoma de Barcelona; ^4^Small Animal Teaching Hospital, University of Liverpool; ^5^The Royal (Dick) School of veterinary Studies, University of Edinburgh; ^6^Anicura Ars Veterinaria; ^7^Hospital Veterinària del Mar; ^8^Hospital Clínico Veterinario, Universidad Europea

The aim of this study was to evaluate the clinical presentation and MRI findings in dogs with cerebellar disease.

Retrospective, multicentric, descriptive study. Clinical data and MRI findings in dogs with cerebellar neurolocalisation and/or cerebellar lesions on were reviewed.

103 patients were included: 59.2% males; 40.8% females; with a median age of 87 months. Crossbreed was the most common breed (17.5%), followed by American Staffordshire terrier (6.8%).

Abnormal mentation and behavior were reported in 9.7% and 27.2% of cases respectively. Ataxia was observed in 75% of patients, mostly cerebellar (36.4%), vestibular (26%) or vestibulo‐cerebellar (22.1%). Other vestibular signs were also frequent: nystagmus (36.9%), strabismus (34%) and head tilt (57.3%). Menace response deficits were noted in 45.6%. Paresis (29.1%) and postural reaction deficits (64.1%) were also observed. As well as Head turn (19.4%) and pleurosthotonus (13.6%).

The most affected regions in non‐diffuse lesions were the right rostral paravermis and the right rostral vermis (44.8% each). Within the vermis, the lobulus centralis and the rostral culmen were the most affected locations (35.5% each).

Vascular disease was the most frequent presumptive diagnosis (45.6%), followed by neoplasia (23.3%), immune‐mediated (14.6%), degenerative (9.7%), congenital (3.9%) and infectious (1.9%).

This study provides a descriptive overview of signalment and localisation of cerebellar lesions in a large dog population and shows a significant incidence of proprioceptive deficits, paresis, head turn, pleurosthotonus and paroxysmal events, that should be considered when localizing in the cerebellum. Most frequently affected areas were the right rostral paravermis and rostral vermis.

## PO19

### Owner compliance, and barriers to compliance, with cage rest, physiotherapy and hydrotherapy, in the conservative management of dogs diagnosed with intervertebral disc disease

#### Brett Noden^2^; Samantha Bell^1^; Anisa Ab Razak^1^; Lisa Alves^1^

**Keyword:** IVDD, Conservative management, Cage Rest, Compliance.

##### ^1^Queen's Veterinary School Hospital, University of Cambridge; ^2^Ontario Veterinary College, University of Guelph

**Introduction:** Intervertebral disc disease (IVDD) is one of the most common neurological conditions reported in dogs, with a lifetime prevalence of 2%–3.5%. Strict cage rest, coupled with adjunctive physiotherapy and hydrotherapy, form the cornerstones of conservative management. It is not known what percentage of owners comply with management recommendations, presenting challenges for assessing the efficacy of conservative approaches. This retrospective questionnaire‐based study aims to evaluate the level of compliance of owners who were recommended conservative therapies, and assess barriers to compliance.

**Methods:** We utilized a standardized, anonymous and objective questionnaire. This was sent to owners of dogs diagnosed with IVDD who were managed conservatively at The Queen's Veterinary School Hospital, Cambridge, UK, between 2018 and 2020.

**Results:** Responses were received from 59 of 145 owners. Of those, 16 were not fully compliant with cage rest recommendations. Common barriers to compliance included beliefs that dogs did not cope well with confined rest (6/16), perception that cage rest was not necessary for recovery (3/16), and preference for less stringent exercise restriction (2/16). Additionally, only 9 owners utilized physiotherapy, and 15 utilized hydrotherapy.

**Discussion/Conclusion:** Overall, 27% of respondents were not fully compliant with cage rest recommendations, often citing concerns for their animal's wellbeing. Furthermore, a lack of veterinary recommendation was the most cited barrier to seeking physiotherapy and hydrotherapy. Overall, this highlights a need for owner education on the importance of compliance with conservative management recommendations and the benefits of adjunctive therapies.

## PO20

### Ex vivo biomechanical assessment of surgical stabilization techniques of the lumbosacral junction in dogs

#### Lucas Smolders^1^; Raphael Arz^1^; Antonio Pozzi^1^; Brian Park^1^

**Keyword:** Degenerative Lumbosacral Stenosis, Biomechanics, Cyclic loading, Pedicle screw‐rod fixation, Screws and polymethylmetacrylate, Neurosurgery, Transarticular screw fixation.

##### ^1^Clinic for Small Animal Surgery, Vetsuisse Faculty, Winterthurerstrasse 260, 8057, Zurich, Switzerland

**Introduction:** Techniques for lumbosacral stabilization include transarticular screw fixation (TSF), pedicle screw‐rod fixation (PSRF), and screws and polymethylmetacrylate (S‐PMMA). It is unclear how these techniques compare with respect to immediate and long‐term biomechanical stability. The study aim was to compare TSF, PSRF and S‐PMMA for non‐destructive, cyclic, and failure loading.

**Methods:** Twenty‐one L7‐S1 canine spine specimens were subjected to non‐destructive biomechanical loading (‐2 Nm/2 Nm) in flexion/extension (FE), lateral bending, and axial rotation. Subsequently, the L7‐S1 joint was stabilized by 1) TSF, 2) PSRF, or 3) S‐PMMA (n = 7 specimens/group). Stabilized specimens were subjected to non‐destructive biomechanical loading, cyclic loading (200 k cycles at 2 Nm, FE), ramped cyclic loading (2.0–5.0 Nm; 50 cycles/moment, FE), and load to failure (flexion). Lumbosacral range of motion (ROM) was recorded for each experimental step and linear mixed models were used to statistically compare test groups.

**Results:** All stabilization methods resulted in a significant, equal reduction in ROM in FE (33.4 ± 10.2° to 2.9 ± 1.2°; *P* < 0.01) and lateral bending (8.1 ± 3.0° to 2.4 ± 0.6°; *P* < 0.01). ROM remained constant throughout continuous cyclic loading for all testing groups (2.3 ± 0.8°). PSRF (7.8 ± 1.6°) and S‐PMMA (7.1 ± 1.6°) showed a significantly lower ROM compared to TSF (11.0 ± 3.6°; *P* < 0.04) after ramped cyclic loading, with progressive screw loosening in 29.0%, 0%, and 85.7%, respectively. PSRF (24.5 ± 1.2 Nm) and S‐PMMA (23.4 ± 3.3 Nm) had a significantly higher load to failure compared to TSF (14.8 ± 8.8 Nm; *P* < 0.04).

**Discussion/Conclusion:** TSF, PSRF and S‐PMMA provide immediate biomechanical stability to the lumbosacral junction. PSRF and S‐PMMA are superior to TSF when subjected to cyclic loading and load to failure.

## PO21

### Idiopathic facial neuropathy in cats: Clinical findings, MRI findings and outcome

#### Maria Stefaniuk^1^; Cecilia Gabriella Danciu^2^; Laura Muñiz Moris^1^; Cristoforo Ricco^1^; Steven De Decker^2^; Josep Brocal^3^; Emili Alcoverro^4^; Oliver Marsh^5^; Frances Taylor‐Brown^6^; Lorenzo Mari^1^

**Keyword:** Facial paralysis, Facial paresis, Facial nerve, Vestibular, Digastricus.

##### ^1^The Ralph Veterinary Referral Centre, Marlow, UK; ^2^Queen Mother Hospital for Animals, Royal Veterinary College, London, UK; ^3^Anderson Moores Veterinary Specialists, The Granary, Bunstead Barns, Hursley, Winchester, UK. and Veterinary Specialty Hospital of Hong Kong, Wan Chai, Hong Kong; ^4^ ChesterGates Veterinary Specialists, Telford Court, Chester, UK. and AniCura Ars Veterinària Hospital Veterinari, Barcelona, Catalonia, Spain; ^5^ DWR Veterinary Specialists, Six Mile Bottom, UK; ^6^Cave Veterinary Specialists, Georges Farm, West Buckland, Wellington

Idiopathic facial neuropathy (IFN) is commonly described in dogs, while information in cats is lacking. Diagnosis is based on exclusion of structural/metabolic neuropathies.

Clinical data of cats diagnosed with IFN were retrospectively multicentrically collected. Thirteen cats were identified, 7‐females, 6‐males, median age 30 months (6–142). Median time‐onset‐to‐presentation was 6 days (1–150).

Facial paresis/paralysis was unilateral (10/13) or bilateral (3/13) (total 16 sides affected). Clinical signs included: absent (11/16) or reduced (2/16) palpebral reflex; lip ptosis (5/16); absent/reduced ear movements (3/6). One cat had corneal ulceration.

8/13 cats had concurrent vestibular signs (5‐unilateral, 3‐bilateral), including head‐tilt (6/13), vestibular ataxia (6/13), head‐swaying (2/13), horizontal nystagmus (3/13).

MRI were assessed by a single author blinded to clinical signs. Imaging findings included thickened (4/16) and/or contrast‐enhancing (7/16) facial nerve and/or smaller caudal belly of the digastricus muscle (10/16). MRI findings matched clinical assessment for 23/26 facial nerves. CSF was normal in 10/11 cats. Main treatments included eye lubrication (11/13) and tapering anti‐inflammatory prednisolone (3/13).

Follow‐up was available for 9/13 cats (median 13 months, 2–91). Median time‐to‐maximal‐recovery was 20 weeks (2–24). Recovery of palpebral function was complete in 7/12 eyes, partial in 2/12 and absent in 3/12. 3/9 cats had persistent lips/face/ears abnormalities. No case developed contralateral facial paralysis after discharge. Ocular discharge developed in one cat. All vestibular signs resolved but one cat was euthanized due to a reported relapse.

IFN affects mostly young cats, often unilaterally and with frequent vestibular signs. Spontaneous improvement is likely, but persistent facial nerve deficits are possible.

## PO22

### ‘A beacon of hope’ in the AI hype storm: A deep learning model for triaging canine brain MRI abnormalities

#### Samira Abani^1^; Holger Volk^1^; Merlin Laue^2^; Peter Dickinson^3^; Steven De Decker^5^; Rodrigo Gutierrez‐ Quintana^6^; Alexej Alexej Hänsch^2^; Gloria Lesta^2^; Aleksandr Subbotin^1^; Kseniia Bocharova^1^; Jörg Janisch^2^; Laura Lemke^1^; Franziska Spohn^1^; Stefanie Winzer^2^; Daniel Sánchez‐Masian^4^; Ehren Mclarty^3^; Adriano Wang‐Leandro^1^; Jasmin Nessler^1^

**Keyword**: Magnetic Resonance Imaging (MRI); Brain; Convolutional Neural Network (CNN); Histopathology; Deep Learning Algorithm, Machine Learning Algorithm.

##### ^1^Department of Small Animal Medicine and Surgery, University of Veterinary Medicine Hannover, Hannover, Germany; ^2^ DOS Software‐Systems, Wolfsburg, Germany; ^3^Department of Surgical and Radiological Sciences, School of Veterinary Medicine, Davis, California, USA; ^4^Hospital Veterinario UCV, Universidad Católica de Valencia San Vicente Mártir, Valencia, Spain; ^55^Department of Clinical Science and Services, Royal Veterinary College, London, United Kingdom; ^6^Small Animal Hospital, School of Biodiversity, One Health and Veterinary Medicine, University of Glasgow, Glasgow, United Kingdom

Artificial intelligence (AI) models for image classification are increasingly being recognized as a potential diagnostic tool. This pilot study utilized a Convolutional Neural Network (CNN) model to classify abnormal versus normal canine brain magnetic resonance images (MRIs), aiming to identify those containing lesions to improve efficiencies in reporting.

The model was trained on a heterogeneous dataset collected from four universities. The dataset consisted of T1W sequences pre‐ and post‐ contrast in transverse, sagittal, and dorsal planes, including.

213 normal (e.g., extracranial etiologies, idiopathic epilepsy, and paroxysmal dyskinesia), and 282 abnormal MRI scans (e.g., neoplasms, inflammatory lesions, and others). Dogs were included if they had a complete clinical diagnosis confirmed either by histopathology or current clinical scientific consensus.

The data was divided into a training set (*n* = 495), a validation set (*n* = 23), and a test set (*n* = 23). The training set comprised 40 744 normal and 14 448 abnormal slices. MRIs were labeled slice‐by‐slice as either normal or abnormal, and subsequently reviewed by a second investigator.

The model correctly classified 495/591 slices as normal and 1259/1878 as abnormal, using in‐person observation as the gold standard. Furthermore, it demonstrated the highest performance in distinguishing between normal and abnormal slices in T1W post‐contrast transverse sequences (True Positive: 158/179, True Negative: 304/458) and the lowest performance in T1W pre‐contrast sequences (True Positive: 118/149, True Negative: 310/528).

The accuracy level in identifying lesions was subpar for the individual image, but improved when sequential images were considered. Further research with a larger dataset is needed to improve the CNN's performance.

## PO23

### Canine meningoencephalitis of unknown origin: objective evaluation by magnetic resonance imaging using the cavalieri method

#### Franziska Spohn^1^; Holger Volk^1^; Adriano Wang‐Leandro^1^; Filip Kajin^2^; Jasmin Nessler^1^

**Keyword:** MUO, MRI, Cavalieri Method

##### ^1^Department of Small Animal Medicine and Surgery, University of Veterinary Medicine Hannover; ^2^Clinic for Internal Diseases, Faculty of Veterinary Medicine, University of Zagreb

Meningoencephalitis of unknown origin (MUO) is a common inflammatory disease of the central nervous system in dogs. Magnetic resonance imaging (MRI) is one of the key diagnostic techniques for a MUO diagnosis. Data on the progression of MRI signs under immunomodulatory therapy are, however, sparse.

This retrospective, single‐center study included 35 dogs with clinically suspected MUO, which received at least one follow‐up MRI. Total lesion volume, number of lesions, clinical signs, and therapy were recorded. Total lesion volume was measured in transversal T2‐ weighted (T2W), fluid attenuating inversion recovery (FLAIR), and volume of contrast up‐ taking lesion measured in T1‐ post contrast sequences using the Cavalieri Method in Image J software (Java‐based Open‐Source‐Software from National Institute of health).

In total 51 follow‐up MRIs were included. MUO was treated with glucocorticosteroids alone or an additional immunosuppressive drug like cyclosporine, cytosine arabinoside. Mean time between initial MRI and follow up MRIs was 342 days (65–‐1943 days). Compared to the previous MRI, lesion(s) visible on worsened in 21 cases, and 30 improved, of which two were normal.

MRIs were performed due to relapse of clinical signs in five dogs, while in 46 cases MRIs were performed as part of routine checkup examination after improvement of clinical signs. Of the latter, 14 dogs experienced a relapse of clinical signs after reduction of immunomodulatory medication after the follow up MRI, of which two dogs had normal MRI befor relapse.

Normal follow‐up MRI might not be a reliable biomarker to predict relapse in dogs with MRI.

## PO24

### Brain‐related presentations are more prevalent in brachycephalic dogs with ‘brachycephalic obstructive airway disorder’ (BOAS)

#### Susana Monforte Monteiro^1^; Lisa Alves^1^

**Keyword:** BOAS, Brain, Hypoxia

##### ^1^Department of Veterinary Medicine, School of Biological Sciences, University of Cambridge

**Introduction/Purpose:** The prevalence of brain‐related disorders in a large population of dogs with brachycephalic airway obstructive disorder (BOAS) has not been described. A previous pilot study on BOAS dogs revealed a high prevalence of seizures. People with obstructive sleep apnoea (OSA), the human equivalent to BOAS, have higher prevalence of epilepsy, brain tumors, and Alzheimer's compared to the general population. This retrospective and prospective study describes the prevalence of brain‐related presentations in brachycephalic dogs with BOAS.

**Methods:** Baseline characteristics, history of brain‐related conditions and BOAS grading data were retrieved from a population of brachycephalic dogs for which BOAS grading is validated (Pugs, English bulldogs, and French bulldogs). All dogs presented for BOAS evaluation between 2008 and 2023 and underwent clinical assessment and respiratory functional grading. The prospective cohort had a neurological examination by a board‐certified neurologist. Dogs were divided into grades, and into clinical BOAS (grades II‐III) and non‐clinical BOAS (grades 0–I).

**Results:** Seven‐hundred and forty BOAS‐affected animals were included as cases. Fifty‐three dogs were graded 0 and included as controls. Eighty‐seven BOAS‐affected dogs showed brain‐related presentations (12%), the majority (89%) of which were seizures/suspected seizures. BOAS‐affected dogs had 6.93 times (CI 95% 0.946‐50.749) the adjusted odds of showing brain‐related presentations compared to BOAS‐free dogs. Dogs with clinical BOAS had 2.48 times (CI 95% 1.37–4.48) the adjusted odds of showing brain‐related presentations compared to non‐clinical BOAS dogs.

**Discussion/Conclusion:** Brachycephalic dogs with BOAS have a high prevalence of brain‐related disorders, and this seems to be associated with BOAS severity.

## PO25

### Role of intravenous intraoperative magnesium sulfate as anti‐inflammatory and analgesic drug in dogs undergoing spinal decompression surgery for acute thoracolumbar disc herniation

#### Guillaume Mallet^1^; Luis Gaitero^1^; Andrea Sanchez Lazaro^1^; Fiona Mk James^1^; Gabrielle Monteith^1^; Geoffrey Wood^2^; Francesca Samarani^1^

**Keyword:** Magnesium sulfate, Analgesia, Anti‐inflammatory, Intervertebral disc herniation, Dogs

##### ^1^Department of Clinical Studies, Ontario Veterinary College; ^2^Department of Pathobiology, Ontario Veterinary College

Magnesium sulfate (MgSO4) reduces the intra‐ and postoperative opioid requirements and reduces levels of IL‐6 and TNF‐a in several procedures in humans including lumbar laminectomy. The analgesic and anti‐inflammatory effects of MgSO4 after spinal surgery have not been studied in dogs. This study investigated the effects of intra‐operative intravenous (IV) MgSO4 continuous rate infusion on postoperative pain and IL‐6 and TNF‐a levels in dogs undergoing thoracolumbar hemilaminectomy for intervertebral disc extrusion (IVDE). A prospective, randomized, blinded, controlled clinical trial recruited 30 client‐owned dogs. Dogs were anesthetized with a standardized protocol. Pain scores were assessed on presentation, 0‐, 4‐, 8‐, 12‐ and 24‐h post‐anesthesia via the modified Glasgow Composite Measure Pain Scale (CMPS‐SF). IL‐6 and TNF‐a levels were assessed on presentation, and 1‐ hour postoperatively. Magnesium group had a lower pain score at 24‐h post‐operatively but did not reach significance (*P* = .08). Magnesium suggested an interaction with age on IL‐6 levels (*P* = .07). TNF‐ a levels were not detectable in the majority of patients and not were not analyzed statistically. Placebo group did not significantly require additional pain medication (*P* = .74). These results suggest that the addition of intra‐operative IV MgSO4 administration may decrease CMPS‐SF score at 24‐h post‐ anesthesia. As results did not reach statistical significance, a larger population may be needed. These results were not correlated to any IL‐6 reduction.

## PO26

### Paroxysmal dyskinesia in 41 dogs: Is the diet change enough?

#### Marika Menchetti^1^; Evelina Burbaitè^1^; Greta Galli^1^

**Keyword:** dyiskinesia, dog, movement disorder

##### ^1^Neurology and Neurosurgery Division, San Marco Veterinary Clinic, Veggiano, Italy

Paroxysmal dyskinesia (PD) describes a group of movement disorders. The diagnosis remains challenging and to date, no specific test can define an etiology. The purpose of this study is to analyze and present the features and the outcome of PD in dogs.

A retrospective case collection was performed. Dogs were included if they had more than one single episode, if a video footage was available, and if they had a complete neurological evaluation, measurement of serum antibodies anti‐transglutaminase‐2 (AGT2), gliadin (AG), and a follow‐up of at least 6 months. Forty‐one dogs have met the inclusion criteria.

The most presented breeds were: mongrel (9/41), Pomeranian (7/41), and Maltese (7/41). The median number of episodes a year was 24 (IQR 12–730). Gastrointestinal issues were observed in 80.5% of the dogs. Both AG and AGT were positive in only 12.2% (5/41). After a diet change to grain‐free, the episodes have disappeared or were less frequent in 92.7% (38/41). However, in 14.6% (6/41) of dogs a relapse within a mean period of 6 months was observed after an initial period of improvement. Half of relapsed dogs had lower episode frequency, in others, episodes became more frequent and severe.

Gastrointestinal issues are a common finding in affected dogs, indicating a possible food intolerance. In our study, 92.7% of dogs responded to diet change while only 51.2% were considered intolerant or partially intolerant to gluten. However, 14.6% of dogs have relapsed after the initial improvement.

## PO27

### See‐saw like nystagmus in NA MRI confirmed achiasmatic golden retriever dog

#### Alexine Lajoie^1^; Jérôme Couturier^1^; Eddy Cauvin^1^

**Keyword:** See saw nystagmus; achiasma; MRI.

##### ^1^Azurvet

**Introduction:** See saw nystagmus is a rare form of nystagmus described as an intorsion and elevation of one eye and simultaneous extorsion and depression of the other eye. In man, the acquired form has been described with masses in the sellar region, infarction of the brain stem and head injury. The congenital form has been associated in humans and in a family of Belgian shephard dogs with achiasma and hemichiasma, referring to complete or partial absence of the optic chiasma.

Case Description.

A 2‐years‐old, entire male Golden Retriever was presented for a nystagmus present since the dog was a puppy. Gait, postural reactions and spinal reflexes were normal on neurological examination. Cranial nerve evaluation revealed a subtle head swaying, inconstant, rapid rotational see‐saw nystagmus and a present but intermittently incomplete menace response in both eyes. Neurological examination was indicative of a lesion affecting the optic chiasma or the vestibular system bilaterally. Complete hematology and biochemistry tests were unremarkable. 1.5 T MRI examination of the brain showed normal optic nerves but an absent optic chiasma replaced by an accumulation of cerebrospinal fluid, consistent with a congenital achiasma.

**Discussion/Conclusion:** See saw nystagmus is a very rare form of nystagmus that has only been previously described in a family of mutant Belgian Shephard dogs. The MRI appearance of an achiasma had not been previously described in dogs.

## PO28

### The use of 3D‐CISS and cine MRI sequences in a dog with obstructive hydrocephalus, and their role in surgical planning

#### Anna Tauro^1^; John Macri^1^; Peter Early^1^; Christopher Mariani^1^; Natasha Olby^1^; Karen Munana^1^; Melissa Lewis^1^; Linda Dillenbeck^2^

**Keyword:** Obstructive hydrocephalus, syringomyelia, tetraparesis, dog, canine, computed tomography, MRI flow study, CINE MRI, 3D‐CISS, VP shunting, obstructive hydrocephalus, lateral aperture.

##### ^1^North Carolina State University; ^2^Colorado State University

**Introduction/Purpose:** This report describes a dog with severe obstructive hydrocephalus and syringomyelia and outlines the use of three‐dimensional constructive interference in steady state (3D‐CISS) and CINE magnetic resonance imaging (MRI) sequences to identify the cause of severe hydrocephalus. Surgical intervention and patient outcome are also described.

**Methods:** A 4‐month‐old male Rhodesian Ridgeback was presented with acute, progressive multifocal signs involving the forebrain, brainstem and cervical spine. Brain MRI was performed, including a sequence for improved anatomical detail (3D‐CISS) and a CINE sequence for evaluating cerebrospinal fluid (CSF) flow (2D FLASH GRE).

**Results:** MRI revealed severe, diffuse ventriculomegaly and syringomyelia. The additional sequences allowed visualization of the patency of CSF flow and the absence of arachnoid septa within the fourth ventricle, ruling out the presence of a fourth ventricle arachnoid diverticulum. Imaging findings supported the diagnosis of hydrocephalus secondary to suspected lateral aperture occlusion. Based on this, ventriculoperitoneal shunting was performed, diverting CSF from the left lateral ventricle into the peritoneum. Clinical signs fully resolved; however, 5 months later, the dog abruptly deteriorated. Computed tomography revealed a breakage of the peritoneal catheter at the level of insertion into the abdomen secondary to marked patient growth. Revision surgery replaced the distal catheter which led to rapid resolution of signs. The patient remains neurologically normal for 11 months post‐ initial surgery.

**Discussion/Conclusion:** Specialized MRI sequences that depict CSF flow dynamics and enhance anatomical detail aided in identifying the presence and location of obstruction of the ventricular system and in planning an individualized surgical approach.

## PO29

### MRI characteristics of the inner ear on post‐contrast fluid attenuated inversion recovery images in dogs without middle and inner ear disease

#### Ya‐Pei Chang^1^; Shi‐Yi Wang^2^

**Keyword:** MRI, FLAIR, post‐contrast, dog, inner ear.

##### ^1^Institute of Veterinary Clinical Science, School of Veterinary Medicine, National Taiwan University; ^2^National Taiwan University Veterinary Hospital, National Taiwan University

The MRI protocol commonly included the FLAIR sequence when examining the brain. Previous human and veterinary studies have demonstrated that post‐contrast FLAIR improves the detection of paraventricular, leptomeningeal, and superficial parenchymal enhancement. However, its value in evaluating the inner ear in dogs has not been investigated. This retrospective study aimed to describe the imaging characteristics of the inner ear on delayed FLAIR images in dogs without middle and inner disease and compare them with post‐contrast T1W images. The imaging database was searched for dogs with head MRI examinations using a 1.5 T unit, including T1W, T2W, FLAIR, post‐contrast T1W, and post‐contrast FLAIR sequences. The post‐contrast FLAIR images, obtained after the post‐contrast T1W images, were referred to as delayed post‐contrast FLAIR (dFLAIR). Ears with structural disease on MRI and dogs with vestibular dysfunction were excluded. A single examiner evaluated all images subjectively. A scoring system of 0‐4 points was designed to summarize the contrast enhancement intensity, pattern, and location for statistical analysis. Sixty‐nine dogs and 131 ears were included. A significantly higher percentage of the inner ear showed enhancement on dFLAIR compared to T1W images (92% vs. 22%). Age and delayed scanning time influenced the presence of contrast enhancement on dFLAIR. However, among dogs with enhanced inner ears on dFLAIR, the overall appearance (low vs. high score) was not affected by either age or scanning time. Our results provide a basis for future investigations into the value of post‐contrast FLAIR images in canine inner ear diseases.

## PO30

### Long‐term follow‐up of surgical management in pug dogs with thoracolumbar myelopathies and concurrent articular facet dysplasia

#### Anna Tauro^1^; Colin Driver^4^; Jeremy Rose^4^; Ricardo Fernandes^3^; Clare Rusbridge^2^

**Keyword:** Pug myelopathy, vertebral stabilization, hemilaminectomy, intervertebral disc protrusion, spinal arachnoid diverticulum, constrictive myelopathy, articular facet dysplasia, paresis, paralysis, plegia, urinary incontinence, fecal incontinence.

##### ^1^North Carolina State University; ^2^Surrey University; ^3^The Ralph Veterinary Referral Centre; ^4^Lumbry Park Veterinary Specialists

**Introduction:** The aim of this follow‐up study is to evaluate the long‐term outcomes of Pug dogs affected by thoracolumbar myelopathy and concurrent articular facet dysplasia, 7 years after surgery. This study is based on earlier research published on dogs that received vertebral stabilization either in isolation or in combination with spinal cord decompression.

**Case Description:** Dogs' caregivers were contacted and requested to complete a questionnaire and provide consent for obtaining clinical history. The questionnaire gathered information on mobility grading after surgery, recurrence of neurological signs, presence of urinary and/or fecal incontinence, cause of death (if applicable), and overall post‐surgery outcome.

**Results:** Out of the 12 dogs' caregivers contacted, 6 participated in the study. Of the 6 Pugs, 5 (83%) regained functional ambulation, while 1 did not and was placed in a dog cart. Among the 5 dogs that improved: Four (80%) experienced recurrence of neurological signs with a median recurrence time of 1.5 years (range 1–4). These dogs died at a median of 2.5 years (range 1–5 years) following vertebral stabilization. Of these, only one was euthanized from causes directly related to the spinal problem. The other three died from unrelated conditions, such as gastrointestinal, respiratory, or organ failure. One dog remained neurologically stable and is still alive (age: 8.9 years), as is the dog using a cart (age: 16.1 years). Additionally, four of 6 dogs had urinary and/or fecal incontinence, and three had recurrent antibiotic‐ resistant urinary tract infections.

**Conclusions:** Vertebral stabilization improved neurological signs in 83% of the dogs; however, this improvement was temporary. Median recurrence of neurological signs was 1.5y post‐STAB. Only one dog died from causes directly related to the spinal problem. Incontinence and recurrent urinary tract infection were the main comorbidities.

## PO31

### Stabilization of atlas and axis in a cat with craniocervical malformation and atlantoaxial instability

#### Julia Hauer^1^; Rodja Jährig^1^; Michael Schmohl^1^

**Keyword:** craniocervical malformation, atlantoaxial instability, stabilization.

##### ^1^Tierklinik Hofheim

Craniocervical malformation and atlantoaxial instability are rare conditions in cats. This case report describes the clinical findings, imaging findings, surgical technique and outcome in an 18‐month‐old cat with a combination of these two conditions. The cat was presented due to progressive tetraparesis and spinal ataxia. At the time of presentation, the cat was just ambulatory for a few steps with intermittent falling to both sides. Neuroanatomical localization was the cranial cervical spine. After clinical and neurological examinations and complete blood work, MRI and CT examinations of the head and neck area revealed a deviation of atlas and axis of approximately 90° ventrally and a following dorsal angulation and cranial dislocation of the dens axis. The myelon was markedly flattened and additional suspected secondary Syringomyelia in this area was identified. Surgery was performed with a ventral approach to atlas and axis, a distraction of both bones was attained and a fixation with two Locking Compression Plates of 1,5 mm size was performed. Post‐surgery x‐rays and CT examination revealed appropriate axial fixation. The cat recovered well from anesthesia and was already ambulatory with marked spinal ataxia the day after surgery. Follow up neurological examinations and x‐rays 10 days, 6 weeks and 12 weeks after surgery revealed appropriate fixation and no signs of complications. The cat was ambulatory with mild spinal ataxia and showed a good quality of life.

## PO32

### Pupillary hippus as a component of post‐attenuation neurological signs in a dog

#### Max Foreman^1^; Rachel Hattersley^2^

**Keyword:** Hepatic Encephalopathy, Neuro‐ophthalmology, Pupillary athetosis, PANS.

##### ^1^Granta Veterinary Specialists; ^2^Dick White Referrals

**Introduction:** Pupillary hippus (pupillary athetosis) refers to the continuous oscillation of pupillary diameter in the absence of light flux variations or other external stimuli. The underlying mechanisms and significance of this phenomenon remains unknown in human medicine, and there are only very rare reports of its occurrence in veterinary medicine.

**Key Findings:** We report the occurrence of pupillary hippus in a four‐year‐old Pug following surgical management of an extrahepatic portosystemic shunt using an ameroid constrictor. Clinical signs consistent with post‐ attenuation neurological signs (PANS) began from 48‐h following surgery, and continuous pupillary oscillations were identified during neurological examination. Treatment consisted of anti‐ seizure medication and supportive care. The dog went on to make a full recovery, with resolution of the pupillary abnormalities.

**Significance:** This is a rare report of pupillary hippus occurring in a veterinary patient and suggests it may be considered as a component of PANS in dogs. Clinicians should assess for the presence in pupillary hippus in cases with subtle signs suggestive of PANS.

## PO33

### Case report: French bulldog with brain biopsy‐confirmed anaplastic oligodendroglioma AND ATYPICAL PLEXUS PAPILLOMA

#### Sarah Gutmann^1^; Kaspar Matiasek^2^; Thomas Flegel^1^

**Keyword:** brain biopsy, neurosurgery, neuronavigation, canine brain tumors.

##### ^1^Department for Small Animals, Leipzig University, Leipzig, Germany; ^2^Section of Clinical and Comparative Neuropathology, Ludwig‐Maximilians‐Universität, Munich, Germany

A 5‐year‐old French bulldog was presented due to acute onset of focal and generalized tonic–clonic epileptic cluster seizures. After initial stabilization with anticonvulsive treatment with diazepam, and phenobarbital intravenously, the dog showed on neurological examination only a mild generalized ataxia and intermitting circling to the left.

Magnetic resonance imaging of the head was showed a T2‐weighted and FLAIR hyperintense region with mild mass effect within the right piriform lobe. After contrast application, a sharply demarcated, contrast‐enhancing lesion became visible within this region (approximately 10 × 9 × 8 mm). Cerebrospinal fluid analysis revealed normal cell count and protein level, but on cytological examination signs of unspecific reaction with few segmented neutrophils, multiple foamy macrophages as well as some rafts of plexus cells were identified. After diagnostic investigation a neoplastic lesion or granulomatous meningoencephalitis were considered as differential diagnoses.

For histopathologic diagnosis two optic‐navigated brain biopsies were taken from the affected region. The histopathologic examination revealed an anaplastic oligodendroglioma with a high rate of mitoses for sample 1 and an atypical plexus papilloma for sample 2. Immediately after brain biopsy, the dog showed a hemi neglect syndrome on the left side and increased circling to the left.

Because of the cautious prognosis the owners declined further treatment using radiation or chemotherapy. That is why the dog was treated just symptomatically with phenobarbital, levetiracteam and low dose prednisolone.

This report shows a rare combination of two brain tumors, which were identified by brain biopsy.

## PO34

### Distribution of neurologic disorders in French bulldogs in a German hospital population—Retrospective study of 285 cases (2020–2022)

Sarah Gutmann^1^; Michelle Menzel^1^; Theresa Mörig^1^; Thomas Flegel^1^

**Keyword:** canine, epidemiology, neurology, neurological diseases

#### ^1^Department for Small Animals, Leipzig University, Leipzig, Germany

The French bulldog (FBD) is one of the most popular breeds, although this breed is associated with several diseases ‐ including variety of neurological problems.

Aim of the retrospective study was to evaluate the distribution of neurologic diseases of purebred French bulldogs presented to the Neuroservice of the Department for Small Animals at Leipzig University between 2020 and 2022 for diagnostic work up.

Of 285 included FBDs, 83.2% showed myelopathy. Intervertebral disc disease (*n* = 163) was the most common neurological disease (57.2% of all recorded FBDs). L2/3 was the most frequently affected intervertebral disc (27.0%), directly followed by L3/4 (26.4%). Other spinal diseases were vertebral malformation (*n* = 123, 43.2% of all FBDs), syringohydromyelia (*n* = 32; 11.2%), lumbosacral stenosis (*n* = 30, 10.5%), spondylosis (*n* = 27, 9.5%), neoplasia (*n* = 12, 4.2%), spinal arachnoid diverticulum (*n* = 12, 4.2%), myelitis (*n* = 7, 2.5%), myelomalacia (*n* = 5, 1.8%) and discospondylitis (n = 4, 1.4%).

Encephalopathies occurred in 103 (36.1%) of all recorded FBDs, of which 31.1% (*n* = 32) had intracranial neoplasms. Dogs with brain neoplasms showed epileptic seizures as cardinal symptom (78.1%), followed by abnormal gait (12.5%), tetraparesis (9.4%) and pain (9.4%). Of 49 FBDs with epilepsy, 55.1% were structural, 28.6% idiopathic, 14.3% unclear and 2.0% metabolic in origin. 9/103 FBD (8.7%) had meningoencephalitis of unknown origin, which was predominantly associated with vestibular signs and central blindness. 7/103 FBD (6.7%) had bacterial meningitis/meningoencephalitis due to ascending otitis media/ interna.

To the authors knowledge, there is just one other study which evaluated neurologic disease in a large cohort of FBDs. The results suggested regional differences.

## PO35

### Clinical presentation, diagnostic imaging findings and outcome of 80 dachshunds with cervical intervertebral disc extrusion

#### Francesca Violini^1^; Federica Tirrito^2,3^; Francesca Cozzi^4^; Barbara Contiero^5^; Simone Anesi^1^; Eric Zini^2,5,6^; Cristina Toni^1^

**Keyword:** intervertebral disc extrusion, dachshund

##### ^1^Willows Veterinary Centre and Referral Service; ^2^ AniCura Istituto Veterinario Novara; ^3^Studio Veterinario Associato Vet2Vet Ferri e Porporato; ^4^Clinica Neurologica Veterinaria ‐ NVA; ^5^Dipartimento di Medicina Animale, Produzioni e Salute—Università degli Studi di Padova; ^6^Clinic for Small Animal Internal Medicine, Vetsuisse Faculty, University of Zurich, Switzerland

Information regarding cervical intervertebral disc extrusion in Dachshunds is scarce. This retrospective multicentric study aims to describe the clinical features, MRI findings and outcomes of Dachshunds diagnosed with cervical IVDE. Medical records were reviewed for signalment, onset of clinical signs, neurological examination, MRI features, treatment and outcome. Eighty Dachshunds were included in the study, mostly ambulatory (55% grade 1 and 33% grade 2) and without nerve root signature (85%). There was a higher percentage of smooth‐haired (41%) and of neutered female and entire male dogs (36% and 34%, respectively). The onset of clinical signs was most often chronic (84%). The most common intervertebral disc space affected was C2–C3 (38%) and foraminal IVDEs were reported in 14% of dogs. A foraminal IVDE was diagnosed in only 25% of dogs presented with nerve root signature. Most dogs (77.5%) were treated surgically. In this group, a higher body condition score and a higher mean spinal cord compression ratio calculated on MRI were directly associated with a longer hospitalization time (*r* = 0.490 *P* = 0.005 and *r* = 0.310 *P* = 0.012). The recovery time was longer in dogs with acute and sub‐ acute onset compared to those with a chronic one (3.1 ± 6.5 days versus 1.6 ± 6.2, *P* < 0.001). Outcome data was available for 83% of dogs. Eighty percent of dogs were considered to have completely returned to normal. There was no association between the therapeutic choice (surgical versus medical management) and the outcome of the dogs included in this study. The present findings can support clinicians managing Dachshund with cervical IVDE.

## PO36

### Occipito‐atlanto‐axial ganglion cyst in a medium‐sized mixed‐breed dog. A case report

#### Daisy Feist^1^; Gudrun Bolln, Dr.^1^; Christoph Andrijczuk^1^

##### ^1^Evidensia Veterinary Clinic Norderstedt

A 10‐year‐old, spayed female mixed‐breed dog (26.7 kg body weight) was presented due to progressively increasing pain in the cervical spine over the past week. The neurological examination was unremarkable except for a mild Horner's syndrome on the left. The MRI examination revealed highly dilated atlantooccipital and atlantoaxial joint capsules on both sides with mainly dorsolateral spinal cord compression at the level of vertebral body C1. A clear, gelatinous material was obtained by CT‐guided puncture. A partial excision of the ventral capsules of the occipito‐atlanto an atlanto‐axial joints through a ventral approach and subsequent removal of the gelatinous content was performed. The surgical resulting in a complete remission of the pain over a period of 2 weeks. The histopathological examination confirmed the suspicion of a ganglion cyst.

At the check‐up 79 days postoperative the bitch was still pain‐free and showed only minor symptoms of Horner's syndrome on the left side.

This case shows that occipito‐atlantoaxial intraspinal ganglion cysts are a rare differential diagnosis in cervical myelopathy.

## PO37

### Thalamic pain syndrome as an unusual clinical sign of canine neosporosis

#### Agnieszka Olszewska^1^; Charlotte Günther^1^; Daniela Farke^2^; Martin Schmidt^2^; Adriana Czerwik^2^

**Keyword:** neosporosis, canine, central nervous system.

##### ^1^Fachtierarztzentrum Koeln Merheim, Olpener Str. 618, 51 109, Cologne, Germany; ^2^ 1Department of Veterinary Clinical Sciences, Small Animal Clinic, Justus‐Liebig‐University, Frankfurter Str. 114, 35 392 Giessen, Germany

**Introduction:** Canine neosporosis in adult dogs frequently manifests as necrotizing cerebellitis, leptomeningtis, encephalitis, or myositis. The most common clinical signs in adult canines include cerebellar and vestibular signs, as well as myalgia.

Thalamic pain syndrome is sparsely described in veterinary medicine, and to our knowledge, has not been associated with canine neosporosis to date.

**Case presentation:** A 3‐year‐old male, neutered Airedale Terrier was presented with acute, progressive signs of diffuse pain not responding to nonsteroidal anti‐inflammatory drugs. The dog was fed a raw meat diet. Clinical examination showed no abnormalities, but mild lethargy and diffuse painful reaction to non‐painful stimuli (allodynia) was observed upon palpation of the entire body.

Full‐body computer tomography scan with contrast revealed no abnormalities, and no changes in the muscles were found, that could explain the clinical symptoms. Despite pain management with gabapentin and paracetamol, no improvement in clinical signs was observed over 1 week, prompting referral for a magnetic resonance imaging (MRI) scan.

MRI of the brain revealed diffuse mild brain atrophy and multifocal, diffuse, bilateral asymmetric necrotizing changes in thalamus, midbrain, and pons.

Cerebrospinal fluid (CSF) examination revealed moderate mononuclear pleocytosis. IgM antibodies against Neospora caninum were moderately increased in serum. Detection of Neospora caninum parasite‐specific DNA by PCR was positive in the CSF. Antimicrobial multiomodal treatment led to no clinical improvement, and the dog was euthanised.

**Discussion/Conclusion:** This case highlights the unusual clinical presentation of adult canine neosporosis, presenting as allodynia in the form of thalamic pain syndrome.

## PO38

### Moraxella osloensis as the first case of vertebral osteomyelitis and epidural empyema in a dog

#### Carmen Aires Serrano^1^; Alba Farré Mariné^1^; Alejandro Luján Feliu‐Pascual^1^

**Keyword:** Osteomyelitis, epidural empyema, M. osloensis, infections, dog

##### ^1^Aúna Especialidades Veterinarias IVC Evidensia

A 7‐year‐old male entire American Bully had a T13‐L1 disc extrusion addressed surgically 2 years earlier and developed subcutaneous Serratia ureilytica infection, resolved with trimethoprim‐ sulfamethoxazole after culture and antibiogram.

Upon presentation, ataxia with severe ambulatory paraparesis and discomfort in the thoraco‐lumbar spine, indicated a T3‐L3 spinal cord lesion, and radiographs revealed osteolytic changes in the operated vertebrae. MyeloCT confirmed fusion of the affected intervertebral space, bony overgrowth, T12‐T13 osteolysis and moderate spinal cord compression by soft‐tissue attenuation. A right T12‐T13 minihemilaminectomy with biopsy and culture of the compressive material revealed pyogranulomatous tissue with the presence of M. osloensis. Cephalexin, paracetamol, meloxicam and pregabalin was initiated and the dog discharged with ambulatory paraparesis 24 h later.

After 6 weeks, the dog deteriorated when meloxicam was interrupted and suffered a generalized tonic‐clonic seizure. A CT scan revealed recurrence of the constrictive myelopathy and a round hypoattenuating non‐contrast‐enhancing lesion in the left temporal cortex compatible with glioma or ischemic infarct.

Medical treatment was changed to amoxicillin‐clavulanic acid, prednisolone and phenobarbital. Six months later, the animal improved to moderate ambulatory paraparesis without discomfort or seizures.

Despite the initial positive response, the prognosis appears guarded due to possible bacterial resistance to the initial antibiotic, and the recurrence of clinical signs highlights the need for long‐term management.

In conclusion, this first case of M. osloensis, an aerobic Gram‐negative bacterium that causes ocular and respiratory infections in people and cattle, causing osteomyelitis and epidural empyema, highlights the clinical and therapeutic complexity of this type of infections.

## PO39

### Does homecare routine change the outcome in dogs following acute non‐compressive nucleus pulposus extrusion, fibrocartilagenous embolism or hydrated nucleus pulposus extrusion?

#### Katherine Phillips^1^; Paul Freeman^1^

**Keyword:** Rest, retrospective.

##### ^1^University of Cambridge

**Introduction:** Acute non‐compressive nucleus pulposus extrusion (ANNPE), fibrocartilaginous embolism (FCE) and hydrated nucleus pulposus extrusion (HNPE) are all conditions presenting with acute to peracute onset of myelopathic signs in dogs and for which treatment usually is medical and conservative; a period of strict rest is often recommended. The aim of this retrospective study was to compare short term outcome of a group of patients specifically instructed to rest and a group encouraged to exercise after diagnosis of ANNPE, FCE or HNPE.

**Methods:** 43 dogs were included. Data was obtained via medical records and telephone calls to referring vets or owners. Dogs were retrospectively assigned to a rest or exercise group depending on their discharge letter instructions with those explicitly told to rest assigned to the rest group and those where rest was not recommended assigned to exercise group.

**Results:** 40 cases survived to discharge with 22 assigned to the exercise group and 18 to the rest group. All dogs recovered and no dogs in either group showed any relapse of signs or deterioration in neurological grade between discharge and their 4‐week recheck. There was no significant difference between the rest and exercise groups.

**Discussion:** Allowing exercise after a confirmed diagnosis of ANNPE, FCE or HNPE did not predispose to relapse of clinical signs in the short term follow up period in this group of dogs affected with ANNPE, HNPE or FCE.

## PO40

### Sacrocaudal meningeal pseudocysts in two dogs

#### Emma Hidalgo Crespo^1^; Alba Farré Mariné^1^; Martí Pumarola^2^; Alejandro Luján Feliu‐Pascual^1^

**Keyword:** pseudo‐cysts, sacrococcygeal, dog.

##### ^1^Neurology/Neurosurgery Service, AÚNA Especialidades Veterinarias hospital, Valencia, Spain.; ^2^Murine and Comparative Pathology Unit. Department of Animal Medicine and Surgery, Universitat Autònoma, Barcelona, Spain

Caudal lumbar meningeal cysts are rarely described in dogs. To the authors' knowledge, only one French Bulldog was reported with a caudal lumbar extradural meningeal cyst. Our aim is to describe the clinical signs, imaging findings, surgical treatment, histopathology, and follow‐up of two dogs with sacrococcygeal meningeal pseudo‐cysts.

A 6‐year‐old Great Dane male neutered with six‐month history of incoordination, ambulatory paraparesis and fecal incontinence presented delayed postural reactions in pelvic limbs, and decreased right hindlimb flexor reflex, bilateral perineal hypoesthesia, and anal sphincter reflexes localizing to L4‐S3. A four‐year‐old American Staffordshire Terrier female spayed with two‐week history of episodic hindlimb cramping/weakness when exited and normal neurological examination. Both exhibited normal physical examination and blood test results and showed isoattenuating, well‐ defined, oval lesions without contrast enhancement inside the vertebral canal from S2 to Cd2 vertebra. Complete macroscopic resection of both cystic‐like lesions with dorsal laminectomy were performed. Both contained CSF‐like fluid and the histopathology revealed connective tissue without epithelial lining in the first dog, and with associated leptomeningeal cell population in the second dog, leading to the diagnosis of meningeal pseudo‐cyst. In the first case, fecal incontinence resolved for 2 weeks, but then relapsed. In the second case, 1 week after the surgery, episodic weakness persisted despite normal neurological examination.

Our two cases demonstrate sacrococcygeal meningeal pseudo‐cysts, not previously described in dogs. Although uncommon in veterinary medicine, and of unclear clinical relevance in our cases, these pathologies should be considered in the differential diagnosis for neurolocalizations at L4‐S3.

## PO41

### Right nerve root C5C6 schwannoma with invasion to the vertebral canal in a Siamese cat. Surgical resolution and histopathological features

#### Xavier Cornet Illa^1^; Sandra Alemany Misserach^1^; Jéssica Molín Molina^2^; Marta López Font^3^; María Ortega Prieto^4^

**Keyword:** feline schwannoma, peripheral nerve seath tumor, modified cervical dorsal laminectomy

##### ^1^Hospital Veterinari de Catalunya; ^2^Departament de Ciència Animal, Universitat de Lleida; ^3^Diagnosi Veterinària; ^4^Anicura Indautxu Hospital Veterinario

**INTRODUCTION:** This study presents a rare case of a nerve root schwannoma in a cat with invasion of the cervical vertebral canal. Only three previous cases have been documented in the literature, highlighting the limited understanding of this pathology in felines. The patient, a 6‐year‐old Siamese cat, initially showed lameness in the right forelimb, which rapidly progressed to monoplegia.

**METHODS:** Magnetic resonance imaging revealed an intradural‐extramedullary lesion in the C5‐C6 region with spinal cord compression, and successful surgical excision was performed via modified dorsal laminectomy. Histopathological analysis of the tumor revealed features consistent with a schwannoma, notably the presence of Antoni A and B patterns. Immunohistochemical studies were positive for SOX10 i S100 and low levels for Ki67.

**RESULTS:** The clinical progression after surgery was very favorable, showing marked improvement in motor function and spinal reflexes in the affected limb. Radiotherapy treatment was carried out afterwards. The patient did not show any clinical or macroscopic recurrence in computed tomography 6 months after surgery.

**DISCUSSION:** The long‐term prognosis of schwannomas in cats remains uncertain, however, the low mitotic index in this case suggests low malignancy, supporting a favorable post‐surgery prognosis. This publication highlights the complexity in diagnosing and managing peripheral nerve sheath tumors in cats, focusing on the importance of detailed case presentations of this little‐known pathology. It underscores the need for future research to fully characterize the clinical progression, histopathological classification, and long‐term prognosis of feline nerve root schwannomas with cervical vertebral canal invasion.

## PO42

### External manual measurement of mandible length to predict the probability of a Pomeranian having syringomyelia

#### Koen Santifort^1,2,4^; Sophie Bellekom^2^; Ines Carrera^3^; Paul Mandigers^1,4^

**Keyword:** Chiari‐like malformation, Syringomyelia, Pomeranian, Morphometry

##### ^1^Evidensia Small Animal Hospital, Arnhem, The Netherlands; ^2^Evidensia Small Animal Hospital ‘Hart van Brabant’, Waalwijk, The Netherlands; ^3^Vet Oracle Teleradiology, Norfolk, United Kingdom; ^4^Expertise Centre of Genetics, Department of Clinical Sciences, Faculty of Veterinary Medicine, Utrecht University, Utrecht, Netherlands

Clinical signs (owner‐reported) have been linked to the likelihood of Pomeranians having or not having Chiari‐like malformation (CM) and syringomyelia (SM). Morphometric studies in this breed are currently lacking. The aim of this study is to describe and analyze morphometric factors of the skull of Pomeranians with and without CM/SM by means of CT as well as manual external measurements.

Ninety‐two Pomeranians >12 months of age were included that underwent both CT and MRI studies of the head and cervicothoracic vertebral column. Two observers independently reviewed the CT imaging studies and performed quantitative measurements. External measurements were taken from the head of dogs when under general anesthesia using a tape measure and a caliper.

Logistic regression analysis revealed that externally measured mandible length was associated with the probability of having SM (*P* = 0.043). Mandible length was moderately correlated with weight (Pearson correlation coefficient = 0.585, *P* < 0.001). The cutoff point on the receiver operating curve at a mandible length of 58 mm had a sensitivity of 96% (95% confidence interval 89‐100%), that is, dogs in this study population with a mandible length shorter than 58 mm were highly likely to have SM.

Manual external measurement of mandible length can be easily performed without sedation or anesthesia in most dogs. The presence or absence of owner reported clinicals signs in addition to the measurement of the length of the mandible could help to determine the probability of a Pomeranian having SM. MRI is still required to formally diagnose SM.

## PO43

### Spinal cord injury from a sewing needle in a cat: A case report

#### Pavlo Karataiev^1^; Mariia Karataieva^1^; Irina Doduh^1^; Ivanna Patsora^1^; Illia Zon^1^; Viktoriia Krivonozhko^1^; Kateryna Holieva^1^

**Keyword:** spinal cord injury, sewing needle

##### ^1^Veterinary clinic Neurocare

A 2‐year‐old DSH cat was admitted with acute, rapidly progressive tetraparesis. Upon examination, tetraplegia, marked hypertonus of all limbs, and neck pain were observed. The neurolocalization indicated the C1‐C5 spinal segments.

On X‐ray, a sewing needle was found in the cervical region, nearly perpendicular to the spine, passing through the vertebral canal at the atlanto‐occipital junction. A CT scan was also performed, providing a better localization of the needle in relation to the surrounding structures.

During intubation, the pharynx area was examined, and although the needle itself was not visualized, a thread was observed running dorsally, likely leading to the eye of the needle.

Surgery was performed using a standard suboccipital approach. A partial dorsal laminectomy of C1 and foramen magnum decompression were performed. The end of the needle was covered with inflammatory tissues. The needle was partially retracted dorsally, the thread was cut and then removed through the oral cavity. During further inspection, another piece of thread was found and removed from the spinal cord. Subsequently, the needle was carefully extracted dorsally.

After surgery, treatment and rehabilitation were carried out in the ICU. The cat was discharged home after a week with improvement, displaying non‐ambulatory tetraparesis. Over the next month, the condition continued to improve, almost to a normal gait.

To the authors' knowledge, spinal cord injury caused by a needle is a rare cause of neurological disorders. However, surgery followed by rehabilitation can significantly improve the neurological status of such patients.

## PO44

### Giant cell glioblastoma mimicking a meningioma: Diagnosis, treatment, histopathology, and outcome of a high‐grade brain tumor in a cat

#### Sergio Sánchez Briones^1^; Alba Farré Mariné^1^; Jessica Molín Molina^2^; Carla Zamora Perarnau^3^; Alejandro Luján Feliu‐Pascual^1^

**Keyword:** Brain tumor, Giant cell glioblastoma, Glial tumor, Meningioma

##### ^1^Neurology/Neurosurgery Service of AÚNA Especialidades Veterinarias hospital, Valencia, Spain; ^2^Serra Húnter Fellow, Department of Animal Science, School of Agrifood and Forestry Engineering and Veterinary Medicine, Universitat de Lleida, Spain; ^3^Diagnostic Imaging Service, Hospital Veterinario UCV Universidad Católica de Valencia, Spain

Intracranial neoplasia has a reported prevalence of 2.2% in the cat. The most common primary feline CNS neoplasm is meningioma and, far less frequent, glioma. Glial tumors can be classified according to the WHO with giant cell glioblastoma (WHO IV) representing less than 5% of reported intracranial neoplasms in humans and have been rarely described in domestic animals.

A 14‐year‐old male neutered cat was referred with a 2‐day history of disorientation, obtundation and behavioral changes. Neurological examination was consistent with a diencephalic lesion. MRI scan revealed a midline well‐defined extra‐axial mass with strong contrast enhancement located at the level of the corpus callosum and falx cerebri compressing the thalamus compatible with a meningioma.

The mass was macroscopically excised through a bilateral parietal approach. Immediate post‐surgical CT confirmed gross resection. The cat recovered uneventfully except for one generalized tonic‐clonic seizure treated with levetiracetam and was discharged 4 days later with normal neurological examination. Histological examination revealed an infiltrative, pleomorphic and anaplastic neoplastic proliferation with giant, multinucleated and karyomegalic cells, suggestive of undifferentiated sarcoma. The neoplastic cell population showed a strong and diffuse immunopositivity reaction to Olig‐2 and GFAP indicating glial origin. Final diagnosis was a giant cell glioblastoma (WHO IV). Six weeks later, the cat's neurological signs returned and developed seizures suggesting tumor relapse and due to its high malignancy was euthanized 45 days after the surgical excision.

In conclusion, this case reports the first cat diagnosed with a giant cell glioblastoma antemortem, its MRI findings and outcome after surgical treatment.

## PO45

### Lumbosacral disc extrusion and associated syringomyelia in a cat

#### Aina Duran Ferrer^1^; Cristian Santos Hernández^1^; Marta Pons‐Sorolla Casanova^1^; Carla Zamora Perarnau^1^; Miriam Martínez Garrido^2^; Daniel Sánchez Masián^1^

**Keyword:** Extrusion, Cat, Syringomyelia.

##### ^1^Veterinary Referral Hospital of The Catholic University of Valencia (UCV); ^2^ ProtonVet Veterinary Radiology

Feline intervertebral disc herniation (IVDH) prevalence ranges between 0.05% and 0.26%. Clinical signs are unusual or underestimated, commonly affecting older cats. Published evidence on intervertebral disc extrusions (IVDE) is limited compared to intervertebral disc protrusions. Most feline IVDH occur caudal to the 11th thoracic vertebra. Protrusions are common at L7‐S1, however IVDE in this location is rare. IVDE secondary myelopathies are unfrequently reported in cats.

An 8‐year‐old neutered‐male domestic cat was referred for 1‐month progressive history of lumbar pain, bilateral pelvic limb muscle atrophy and flaccid tail. Neurological examination revealed mild ambulatory paraparesis, though it was limited due to behavioral issues.

Magnetic resonance imaging of thoracolumbar and lumbosacral spine was performed. T2W and T1W hypointense L7‐S1 extradural material dorsal to the L7‐S1 disc was found, causing secondary complete obliteration of the vertebral canal. Meningeal enhancement was seen surrounding this material. Central canal distension from L4 to L5 was visible, showing T2W hyperintense and T1W hypointense cord signal consistent with syringomyelia.

L7‐S1 dorsal laminectomy was performed removing the extruded material. Lumbosacral intervertebral disc was not fenestrated as the spinal cord extended caudally to the IVDE and did not allow a proper visualization of the disc. One week after surgery the patient showed no pain and 1 month later, he regained tail motion, showing a normal gait without neurological deficits after 2 months.

To authors knowledge, this is the first case report of lumbosacral IVDE with secondary syringomyelia in a cat. Successfully outcome was achieved through decompressive surgery, as previously reported in feline IVDE.

## PO46

### Histopathological findings of neurolymphomatosis as an unusual presentation of allodynia and weakness in a dog without changes in diagnostic imaging—Case report

#### Adriana Czerwik^1^; Agnieszka Olszewska^2^; Elena Kloß^3^; Christiane Herden^3^; Martin Jürgen Schmidt^1^

**Keyword:** neurolymphomatosis, dog, allodynia

##### ^1^Department of Veterinary Clinical Sciences, Small Animal Clinic, Justus‐Liebig‐University, Frankfurter Str.114, 35 392 Giessen, Germany; ^2^Fachtierarztzentrum Koeln Merheim, Olpener Str. 618, 51 109, Cologne; ^3^Department of Veterinary Pathology, Small Animal Clinic, Justus‐Liebig‐University. Frankfurter Str. 96, 35 392 Giessen, Germany

Neurolymphomatosis is a rare condition defined as infiltration of peripheral nerves or nerve roots by neurotropic lymphoma. In humans, one of the basic clinical presentations is painful polyneuropathy/polyradiculopathy. The aim of this study was to present the neurological condition and diagnostic work‐up, as well as histopathological findings of a dog with neurolymphomatosis and angiotropic lymphoma.

A 7‐year‐old Spanish Greyhound was presented with a history of progressive signs of weight loss despite a normal appetite, signs of unlocalized pain, weakness of the pelvic limbs, and apathy. The clinical examination revealed generalized cachexia, apathy, severe tremors of the pelvic limbs upon standing, and allodynia of the lumbar vertebral column. The neurological localization was diffuse.

Magnetic resonance imaging of vertebral column was normal. Red blood count and biochemical analysis revealed moderate polycythemia vera, increased calcium, and CSF blastocytes with no pleocytosis. The owners opted for euthanasia. In the histopathological examination, the vessels of the spinal cord were filled with blastoid round cells. Additionally, from L5 increasing caudally, there was infiltration of the nerves and spinal ganglia with blastoid round cells, especially in the sciatic nerve. The same changes were found in the vessels of the forebrain, kidneys, and skeletal muscles. The diagnosis was neurolymphomatosis and angiotropic malignant lymphoma with involvement of central and peripheral nervous systems, and secondary polycythemia.

Nervous system lymphoma (NSL) is reported to be uncommon in dogs. The current case report describes that in the case of NSL, MRI findings may appear normal, and the prognosis tends to be guarded.

## PO47

### Atypical form of steroid responsive meningitis arteritis extended to severe myelitis with micro bleeding—Case report

#### Adriana Czerwik^1^; Agnieszka Olszewska^2^; Daniela Farke^1^; Anna Katarzyna Siwicka^1^; Martin Jürgen Schmidt^1^

**Keyword:** SRMA, dog

##### ^1^Department of Veterinary Clinical Sciences, Small Animal Clinic, Justus‐Liebig‐University, Frankfurter Str.114, 35 392 Giessen, Germany; ^2^Fachtierarztzentrum Koeln Merheim, Olpener Str. 618, 51 109, Cologne

Steroid‐responsive meningitis‐arteritis (SRMA) is an immunopathologic condition in young, adult dogs with described typical, acute, and atypical form. In the latter, the dogs can show neurological deficits and there are not many reports about it. Presented case describes neurological condition and follow‐ up of protruded SRMA case.

An 8‐month‐old mix‐breed dog was presented with a history of fever, apathy, low head carriage, progressing over 2 weeks. Despite fever and generalized hyperesthesia, the neurological examination was normal. The next day, before diagnostic work up, the dog showed acute worsening to non‐ ambulatory, flaccid paraplegia with intact nociception, absent perineal reflex, reduced muscle tone of the tail, allodynia of the lumbar vertebral column and hyperesthesia of the neck. The neurological localization was multifocal.

Magnetic resonance imaging of vertebral column showed diffuse, intramedullary oedema at the level of Th12 to L7 with moderate, inhomogeneous contrast enhancement within spinal cord as well as the meninges. In T2* sequence there was focal signal void within the spinal cord. CSF showed increased protein and purulent pleocytosis. IgA antibodies in serum and CSF were increased. The infectious profile (PCR) was negative. The diagnosis was SRMA extended to severe meningomyelitis with intramedullary bleeding.

Immunosuppressive treatment with prednisolone and arabinoside cytosine led to improvement of the hyperesthesia and systemic signs, but not of the paraplegia. Due to paraplegia and fecal incontinence decision for euthanasia after 2 months was made.

The current case report describes atypical severe extended form of SRMA with a guarded prognosis for neurological improvement despite aggressive treatment.

## PO48

### Suspected central horner syndrome in a dog with a paramedian thalamic lacunar ischaemic infarct

#### Lucia Castro Carnes^1^; Rodrigo Gutierrez Quintana^1^; Gawain Hammond^1^; Vicente Aige Gil^2^; Catherine Stalin^1^; Adriana Kaczmarska^1^

**Keyword:** Horner syndrome, Ischemic infarct, Neuro‐ophthalmology, Thalamus

##### ^1^Division of Small Animal Clinical Sciences, School of Biodiversity, One Health and Veterinary Medicine, College of Medical, Veterinary and Life Sciences, University of Glasgow; ^2^Facultad de veterinaria, Universidad Autonoma de Barcelona

Horner syndrome (HS) is a neuro‐ophthalmic condition resulting from disruption of the oculosympathetic pathway, characterized by miosis, ptosis, enophthalmos, and 3rd eyelid protrusion. Central HS is uncommon and can be identified based on the presence of associated hypothalamic, brainstem, or spinal cord signs.

A 13‐year‐old male crossbreed presented with a one‐month history of acute onset left‐sided HS and disorientation. Neurological examination confirmed HS and revealed pelvic limb ataxia and proprioceptive deficits.

MRI revealed a sharply delineated, comma shaped T2W hyperintense lesion in the dorsomedial aspect of the left thalamus, most consistent with a paramedian thalamic lacunar ischemic infarct in the territory of the caudal perforating arteries arising from caudal communicating artery. The absence of significant findings in cervical MRI and thoracic CT supported the infarct as the likely primary etiological factor for the clinical presentation. Subsequently, hypertension and proteinuria were identified, indicating their role in the pathogenesis of the ischaemic event, and further contributing to the diagnosis.

Besides the reported case, in a study on 16 dogs with three types of thalamic syndromes one dog with paramedian thalamic infarct also had HS. Similar studies are described in humans. The pathophysiology of HS related to paramedian thalamic lesion is unknown but could be explained by disruption of nearby associated neural networks that indirectly affect the sympathetic pathway.

In conclusion, HS secondary to thalamic stroke should be considered when evaluating dogs exhibiting HS. Any occurrence of HS in the context of thalamic lesion warrants further research to determine the precise pathways involved.

## PO49

### Chronic trismus in a cat with an extra‐axial mass causing compression of the pons

#### Heidi Thatcher^1^; Joana Tabanez^1^; Victoria Coates^1^; Lorna Arrol^1^

**Keyword:** Trismus, pontine lesion, cat

##### ^1^Lumbry Park Veterinary Specialists

This report describes a chronic case of trismus in a feline patient secondary to a single extra‐axial mass at the level of the pons. Chronic masticatory myositis, temporomandibular joint disease (TMJ) and tetanus are common differential diagnoses of trismus. We aim to report a rare occasion of a pontine lesion causing inability of opening the jaw without significant muscle atrophy or fibrosis.

A 15‐year‐old, female, neutered, domestic short hair (DSH) presented with a 5‐month history of initial dysphagia that, further progressed to an inability to open the mouth. This resulted in difficulty eating and weight loss. On neurological examination the patient was ambulatory with proprioceptive ataxia, cumbersome hopping responses, a mild head tilt towards the right and positional rotatory nystagmus. The mandibular range of motion was 20 mm, which is considerably less than the expected value in a healthy cat (62 ± 8 mm (median 63, range 41–84). Diagnostic imaging revealed a single, extra‐axial mass at the level of the pons. Unfortunately, concerns over the patient's quality of life resulted in humane euthanasia without the option of post‐mortem histopathology. There are rare reports of trismus associated to pontine lesions, more commonly ischaemic incidents. This is likely caused by stimulation, either loss of inhibition or facilitation, of the motor nucleus of the trigeminal nerve.

A pontine lesion, although rare, is an important differential diagnosis to be considered in cases of trismus within the feline population.

## PO50

### Vertebral vascular canal dysplasia in a pug dog: Clinical presentation and imaging characteristics

#### Amy Pergande^1^; Francesca Raimondi^1^

**Keyword:** Dysplasia, Vertebral malformation, Vascular, Anomaly

##### ^1^Southern Counties Veterinary Specialists, UK

**Introduction/Purpose:** Vertebral malformations and anatomical anomalies are common among brachycephalic breeds. Vertebral vascular canal dysplasia (VVCD) is an anomaly previously described in both English and French bulldogs, but not yet reported in pugs.

**Methods:** An 8‐year 5‐month‐old female pug presented with a chronic progressive history of pelvic limb gait changes consistent with a non‐painful T3‐L3 myelopathy. Investigations performed included hematology and biochemistry panels and cross‐sectional imaging (MRI and CT).

**Results:** MRI and CT identified diffuse thoracic caudal articular process (CAP) dysplasia, with focal sites of secondary constrictive myelopathy considered to be causative of the patient's signs. Multifocal osseus defects were also identified at the level of the mid body of multiple vertebrae, in the location of the vascular canal. Seven vertebrae (T10‐L2 and L5) showed defects extending to more than 50% of the vertebral body height, with significant lateral widening of the vascular canal. MRI signal of tissue within the osseus defects was hyperintense on T2W and T1W sequences, consistent with previous reports identifying the presence of fat on imaging and post‐mortem evaluations of cases with VVCD. The patient subsequently underwent a T7‐T13 vertebral stabilization as treatment of the observed CAP dysplasia and constrictive myelopathy.

**Discussion/Conclusion:** This case demonstrates the presence of lesions in a pug dog consistent with previous descriptions of VVCD in bulldogs. In this instance it was not believed to be causative of the patient's clinical signs, but further investigation into the prevalence and clinical significance within this breed is warranted.

## PO51

### Combined malonic and methylmalonic aciduria (CMAMMA) in a pomeranian dog

#### Sara Peverelli^1^; Guillaume Marc Albertini^2^; Max Foreman^3^

**Keyword:** Organic aciduria, Malonic, Methylmalonic, Pomeranian

##### ^1^Fitzpatrick Referrals Ltd, Godalming, United Kingdom; ^2^Movement Referrals, Runcorn, United Kingdom; ^3^Granta Veterinary Specialists, Linton, United Kingdom

Combined malonic and methylmalonic aciduria (CMAMMA) is an inborn error of metabolism that has been reported rarely in people and can manifest with a variety of non‐specific clinical signs. In veterinary medicine, CMAMMA has only been reported in one Labrador Retriever with progressive encephalomyelopathy. CMAMMA has never been reported in Pomeranians.

We describe a case of CMAMMA in a 10‐month‐old male entire Pomeranian, presented with a history of transient ambulatory tetraparesis and generalized proprioceptive ataxia and persistent thoracic limb hypermetria. Neuroanatomical localization was multifocal to the C1‐C5 segment and cerebellum. MRI of the brain revealed bilateral symmetrical T2w hyperintense, T1w hypointense and non‐contrast enhancing lesions affecting the caudate nuclei, thalamus, rostral colliculi, and medulla oblongata. Chiari‐like malformation and severe cervical syringomyelia were also identified. Additional investigations including serum biochemistry, cerebrospinal fluid analysis, serum thiamine, cobalamin, glucose, lactate, carnitine and plasma amino acids were unremarkable. Quantitative urine organic acid analysis revealed significant elevation of methylmalonic (32 mmol/mol creatinine; RI 0–9 mmol/mol creatinine) and malonic (21 mmol/mol creatinine; RI 0–1 mmol/mol creatinine) acids. Three months after dietary restriction of protein, methylmalonic and malonic acid levels were 30 and 5 mmol/mol creatinine respectively. Whole genome sequencing is pending.

This is the first case report describing CMAMMA in the Pomeranian breed, and the first with an MRI study. In humans, mutations in the ACSF3 gene is a common cause, necessitating further genetic studies in dogs. CMAMMA should be considered in young dogs with multifocal neuroanatomical localization and symmetrical intra‐axial changes.

## PO52

### Presuntive multiple discospondylitis in black bear (*Ursus tibethanus*)

#### Ares Burballa^1^; Carla Zamora^2^; Daniel Sanchez‐Masian^1^; Ferran Alegre^3^

**Keyword:** Ursus tibethanus, Asian Black Bear, multiple discospondilytis

##### ^1^Unavets; ^2^Universidad Catolica de Valencia; ^3^Animal Asia

A 15‐year‐old Black Bear male (*Ursus tibethanus*) was presented with a history of 3‐months of unspecific signs of pain and progressive difficulty to walk. Previously he was treated for carpus septic arthritis. On hands‐off neurological examination, pelvic limb paresis and ataxia, kyphosis and unusual postures were detected and no improvement after analgesic treatment was noted. Signs progressed to tetraparesis, low neck carriage and recumbency. Based on neurological examination, cervical lesion was suspected but a thoracolumbar disease couldn't be excluded.

Spine radiographic findings showed irregular endplate synthesis with extension into the vertebral body of L1‐L2, peripheral sclerosis to the endplate synthesis and collapse of the intervertebral disc space. Due to results, a CT‐scan of the spine was performed.

In L1‐L2 there is evidence of erosion of the endplates bilaterally, multifocal osteolysis, periosteal proliferation, involvement of a third of the vertebral bodies with osteolysis and sclerosis. Intervertebral disc extrusion and hemorrhagia and empiema inside the vertebral canal were also detected. Less severe findings were noted also in C3C4 and L7S1 intervertebral spaces.

A presumptive diagnosis of multiple discopondilytis was established. A possible origin of those lesions could be the previous arthritis. Based on previous synovial fluid culture, antibiotic therapy was started without improvement and elected euthanasia was performed.

This is the first report of presumed discopondilytis in a Black bear. The authors suspected as a possible cause of those lesions the previous septic arthritis however, no vertebral aspiration cytology/culture or blood culture were done.

## PO53

### Clinical presentation, diagnosis, treatment and follow up of two Asian black bears (*Ursus tibethanus*)

#### Ares Burballa^1^; Carla Zamora^2^; Daniel Sanchez‐Masian^1^; Ferran Alegre^3^

**Keyword**: Ursus tibethanus, Asian Black Bear, multiple discospondilytis

##### ^1^Unavets; ^2^Universidad Catolica de Valencia; ^3^Animal Asia

A 15‐year‐old Black Bear male (Ursus tibethanus) was presented with a history of 3‐months of unspecific signs of pain and progressive difficulty to walk. Previously he was treated for carpus septic arthritis. On hands‐off neurological examination, pelvic limb paresis and ataxia, kyphosis and unusual postures were detected and no improvement after analgesic treatment was noted. Signs progressed to tetraparesis, low neck carriage and recumbency. Based on neurological examination, cervical lesion was suspected but a thoracolumbar disease couldn't be excluded.

Spine radiographic findings showed irregular endplate synthesis with extension into the vertebral body of L1‐L2, peripheral sclerosis to the endplate synthesis and collapse of the intervertebral disc space. Due to results, a CT‐scan of the spine was performed.

In L1‐L2 there is evidence of erosion of the endplates bilaterally, multifocal osteolysis, periosteal proliferation, involvement of a third of the vertebral bodies with osteolysis and sclerosis. Intervertebral disc extrusion and hemorrhagia and empiema inside the vertebral canal were also detected. Less severe findings were noted also in C3C4 and L7S1 intervertebral spaces.

A presumptive diagnosis of multiple discopondilytis was established. A possible origin of those lesions could be the previous arthritis. Based on previous synovial fluid culture, antibiotic therapy was started without improvement and elected euthanasia was performed.

This is the first report of presumed discopondilytis in a Black bear. The authors suspected as a possible cause of those lesions the previous septic arthritis however, no vertebral aspiration cytology/culture or blood culture were done.

## PO53

### Clinical presentation, diagnosis, treatment and follow up of two Asian black bears (*Ursus tibethanus*)

#### Ares Burballa^1^; Daniel Sanchez‐Masian^1^; Shaun Thomson^3^; Ferran Alegre^2^

**Keyword:** idiopathic epilepsy, epilepsy classification, Asian Black Bear, Ursus tibethanus.

##### ^1^Unavets; ^2^Animal Asia; ^3^Vietnam Bear Rescue Center. Animal Asia Foundation

Two 12‐year‐old black bears (ursus tibethanus), one male and one female, were investigated for epileptic seizure activity. The epileptic activity was characterized by recumbency, decrease mental status, vocalization, hypersalivation and generalized tonic‐clonic movements. Physical examination and hands off neurological examination between events was unremarkable. General blood work including hematology and biochemistry, pre and postprandial bile acids, toxoplasma titres (IgG/IgM), Chest radiographs and abdominal ultrasonography were reported as unremarkable.

Phenobarbital treatment was started in both cases and blood levels rechecked every 6 months. In the male bear, the starting phenobarbital dose was 2.5 mg/kg/BID but had to be increased till 5 mg/kg/BID and potassium bromide was added due to poor seizure control. In the female bear, a seizure‐free period has been achieved for 2 years with the initial phenobarbital dose of 2.7 mg/kg/BID.

This is the first report of presumed idiopathic epilepsy (tier 1 confidence level) and antiseizure treatment monitoring in black bear (Ursus Tibethanus).

## PO54

### Surgical intervention for multiple depressed skull fracture fragments causing laceration and penetration of the brain parenchyma following a car accident in a dog

#### Amy Pergande^1^; Francesca Raimondi^1^

**Keyword:** Traumatic brain injury, Skull fracture

##### ^1^Southern Counties Veterinary Specialists, UK

**Introduction/Purpose:** Traumatic brain injury (TBI) is an alteration of brain function resulting from external injury. Patients with cranial fractures depressed greater than the thickness of the cranium should undergo surgery to prevent infection.

**Methods**: A 3‐year‐old spayed female CKCS presented after being hit by a car. The patient was stuporous, with a modified Glasgow coma scale (MGCS) score of 12. Conscious CT was performed, and the patient was stabilized with mannitol, broad‐spectrum antibiotics, antiepileptic and pain relief medications for 24 h with a subsequent improvement of MGCS score to 16.

**Results:** CT revealed multiple depressed fractures of the cranial vault affecting the right frontal, parietal and temporal bones and in the wing of the basisphenoid bone. Most fractures were comminuted with multiple displaced bone fragments. A large bone fragment originating from the parietal bone was markedly displaced caudomedially in relation to the calvarium, causing compression of the brain parenchyma. Surgery was performed to remove the penetrating bone fragments. The large fragment was drilled into two smaller fragments to avoid excessive manipulation, and the extensive dural defect was autografted with temporalis muscle fascia. The calvarial defect was covered with PMMA secured by three monocortical 1.5 mm screws.

The patient showed a dramatic improvement and was discharged 5 days post‐surgery. At re‐ examination after 1 month the owner reported blindness in the left eye and otherwise normal neurological status.

**Discussion/Conclusion:** This case discusses techniques used in the management of a patient with traumatic skull fractures, including autograft of a dural defect.

## PO55

### Clinical presentation, advanced imaging findings and successful treatment of a suspected infectious meningoencephalitis in a king penguin (*Aptenodytes patagonicus*)

#### Albert Aguilera Padros^1^; Joao Miguel De Frias^1^; Barbara Ferreira^2^; Simon Girling^2^; Tiziana Liuti^1^; Stephanie Mota^2^

**Keyword:** meningoencephalitis, exotic animal, fungal, MRI features, paroxysmal episodes

##### ^1^The Royal (Dick) School of Veterinary Studies, The University of Edinburgh; ^2^The Royal Zoological Society of Scotland, RZSS Edinburgh Zoo

**Case presentation.**

A 9‐year‐old, male, captive‐born King penguin (Aptenodytes patagonicus) was presented with progressive nine‐days of lethargy, anorexia and 7 abnormal paroxysmal episodes. These events were characterized by decreased awareness, right sided head tilt and cervical turn, left flipper abnormal tonic horizontal position, lasting 1–5 min. Neurological examination was unremarkable. Episodes were considered likely a focal epileptic seizure, although an unusual movement disorder or vestibular event could not be excluded.

Investigations included hematology, serum biochemistry, plasma protein electrophoresis (PPE), serum vitamin A, E and B1 concentrations. Abnormalities included a significant heterophil‐dominated leucocytosis, and the PPE results suggestive of an inflammation. A full body computed tomography (CT) and head magnetic resonance imaging (MRI) were performed. Thoracic and abdominal CT were unremarkable. Head CT and MRI revealed a large left telencephalic intra‐axial lesion. MRI findings included a signal void centred lesion with associated perilesional oedema and meningeal contrast enhancement.

Considering high prevalence of Aspergillus spp. infection in penguins, inflammatory changes and advanced imaging features, a presumptive diagnosis of infectious meningoencephalitis was suspected. Treatment was started with voriconazole, amoxicillin‐clavulanic, enrofloxacin, omeprazole and gabapentin.

Last focal epileptic seizure reported was 4 days after diagnosis. Gabapentin was stopped on day 20. Follow‐up after 4 weeks revealed a normal hematology and PPE. Antibiotics were discontinued after 12 weeks. At the time of writing, 4‐months post‐diagnosis, no further episodes were recorded.

**Conclusion:** To the authors' knowledge this is the first report which describes MRI features and successful treatment of a suspected infectious meningoencephalitis in a King penguin.

## PO56

### Surgical resolution of lumbosacral subluxation secondary to articular process aplasia in a Maltese dog

#### David Martínez Jiménez^1^; Alba Farré Mariné^1^

**Keyword:** Vertebral articular processes aplasia, Lumbosacral subluxation.

##### ^1^Aúna Especialidades Veterinarias IVC Evidensia

Vertebral articular processes aplasia (APA) is widely described at the caudal thoracic vertebras in veterinary literature, but not affecting the lumbosacral joint.

An 8‐year‐old Maltese dog was with lumbosacral pain of 1 year's duration showed. A neurologic exam with kyphosis, ambulatory paraparesis, reduced withdrawal reflex and perineal reflexes bilaterally, and severe lumbosacral pain, consistent with an L6‐S3 neurolocalization. Main differential diagnoses included intervertebral disc disease, discospondylitis, nerve sheath tumor, compressive transitional vertebra and yuxta‐articular cysts. Blood work‐up results were within reference limits and radiographs of the lumbar spine showed lumbosacral subluxation. Subsequently, a computed tomography of the area showed bilateral aplasia of L7 and S1 articular facets joint with overlapping of the dorsal lamina of the sacrum, narrowing of the vertebral canal and cauda equina compression.

The malformation was surgically addressed with traction and lumbosacral fixation with cortical screws and bone cement. Although attempted, complete anatomical reduction was not possible due to the chronicity of the condition. All neurological deficits resolved 7 weeks after surgery. Eight months later, the dog remains neurologically normal and pain‐free.

Although, to our knowledge, APA has not been previously described affecting the lumbosacral joint, other malformations such as transitional vertebra are frequent at that location, and the biomechanical consequences and surgical treatment are similar.

Lumbosacral APA should be considered as a differential diagnosis in dogs with signs consistent with an L6‐S3 neurolocalization, even in mature dogs. Moreover, surgical stabilization may lead to long‐term resolution of clinical signs as in our case.

## PO57

### Cranioplasty using a pre‐ modeled cutting guide and custom titanium plate designed with 3D digital modeling for resection of multilobular tumor of bone in a dog

#### Juan Aires Serrano^1^; Juan Aires Prieto^1^; Carmen Aires Serrano^1^; Donato Monopoli Forleo^2^; Adrián Berriel Vilas^2^; Hernando Afonso Martín^2^

**Keyword:** Pre‐modeled cutting guide, Multilobular tumor of bone, Cranioplasty, Pre‐modeled implant

##### ^1^Hospital Veterinario Puente Genil; ^2^Biosurgex

A 7‐year‐old female medium crossbreed dog presented with a rapidly growing mass in the caudo‐ dorsal part of the skull, indicative of MLO as suggested by a previous histopathology study. Neurological symptoms were absent, and pre‐anesthesia evaluation revealed normal parameters.

MLO requires prompt intervention, often through surgical resection. Cranioplasty post‐tumor removal safeguards the brain and enhances aesthetics. Traditional methods of cranioplasty involve muscle or fascia flaps with or without a polymethylmethacrylate cap, intraoperatively adapted nets, and pre‐ modeled titanium mesh.

In our case, CT scans of the skull and chest were conducted under general anesthesia for surgical planning and to exclude possible metastasis. The surgery was scheduled and performed within 2 weeks, utilizing a personalized cutting‐guide and a 3D pre‐designed titanium implant crafted to mimic the caudo‐dorsal skull shape, resembling the sagittal crest. A post‐operative CT scan confirmed the accurate placement of the implant.

This is the first reported case in which the resection and subsequent cranioplasty were performed utilizing a pre‐modeled cutting guide, facilitating precise resection with a 30% safety margin based on the original tumor size. This approach differs from prior studies, where conventional equipment was used for craniotomy.

One year post‐operatively, the patient shows no signs of tumor recurrence.

In conclusion, this first reported case of MLO resection and cranioplasty using a personalized cutting‐ guide and pre‐modeled implant demonstrates enhanced surgical precision and improved aesthetic outcomes for patients undergoing cranioplasty.

## PO58

### Congenital spinal epidural lipomatosis in a 1‐year‐old Siberian husky dog and long‐term outcome with conservative treatment

#### Sergio Moya García^1^; Nuria Delgado^1^; Jose Miguel Segura Navarro^2^; Jose Javier González^3^; Juan Carlos Cartagena^4^

**Keyword:** lipomatosis, epidural, congenital, Siberian Husky.

##### ^1^Neurology Department Bluecare Partners Hospital; ^2^Nexo Menescal; ^3^Vet&Vet; ^4^Oncology Departament Bluecare Partners

This case describes a spinal cord epidural lipomatosis in a 1‐year‐ old non neutered male Siberian Husky dog and the evolution after 1 year with conservative treatment.

The patient was admitted to the neurology department of the hospital due to proprioceptive ataxia 6 month ago. Orthopedic diseases have been ruled out by an orthopedic specialist.

Neurological examination revealed paraparesis, proprioceptive ataxia affecting the hind limbs and thoracolumbar paraspinal pain. Spinal reflexes and postural reactions were normal.

Our neurolocalisation was T3‐L3 myelopathy, with a congenital and inflammatory/infectious diseases being the most likely cause.

Computed tomography (CT) and magnetic resonance imaging (MRI) images identified large extension dorsal compression of the thoracolumbar spinal cord by hypertrophic. Cerebrospinal fluid (CSF) analysis revealed no pleocytosis and infectious diseases were ruled out by PCR. Spinal decompression surgery was ruled out because of the extent of compression but a hemilaminectomy was performed for biopsy and histopathological study. Histopathological examination of epidural lesions identified extensive well‐differentiated mature adipose tissue.

Due to the extent of compression and the age of the patient, conservative treatment with gabapentin at 10 mg per kg every 8 h and a low‐fat diet was chosen. After 1 year with this treatment the patient showed no worsening of clinical signs.

To the authors' knowledge this is the first described case of congenital epidural lipomatosis in dogs. Prednisolone is a reported predisposing factor in humans with lipomatosis and it has also recently been described in dogs with prolonged steroid treatment.

## PO59

### Adult‐onset neuronal ceroid lipofuscinosis (NCL) in a smooth‐haired Dachshund

#### Diogo Gouveia^1^; Emilie Cloup^1^; Max Foreman2

**Keyword:** neuronal ceroid lipofuscinosis, Dachshund, adult‐onset, genetic testing, histopathology, electronic microscopy.

##### ^1^Dick White Referrals; ^2^Granta Veterinary Specialists

**Introduction:** A 5‐year‐old smooth‐haired Miniature Dachshund presented with a two‐week history of episodes of aggression towards objects, head pressing, circling, and intermittent pelvic limb ataxia. Visual impairment had been suspected several months prior.

**Presentation.**

Physical examination was normal, but the neurological examination revealed depressed mentation, central blindness (bilaterally absent menace responses with intact pupillary light reflexes) and bilaterally absent nasal mucosal nociception. Bilateral forebrain disease was suspected.

**Results:** Blood, urine analysis and CSF analysis and brain magnetic resonance imaging were unremarkable. The patient was euthanised due to development of generalized seizures.

Accumulation of intracellular neuronal autofluorescent granular material was identified during histopathology, most frequently in the thalamus and hypothalamus. Multiple variably sized electron‐ dense granules were further characterized with electronic microscopy. These microscopic findings were compatible with the diagnosis of a neuronal ceroid lipofuscinosis (NCL).

Genetic testing for mutations in the canine PPT1 and TPP1 genes was negative.

**Discussion/Conclusion:** Adult‐onset NCL has only been described in three Dachshunds where genetic testing was not available. The authors believe this case might represent a novel subtype of NCL in the Dachshund breed, caused by a currently unidentified genetic mutation. NCL should be considered a differential diagnosis, regardless of breed and age, in cases of progressive visual impairment, abnormal and/or aggressive behavior, cognitive or motor decline, cerebellar ataxia, sleep disturbances and seizures. Histopathology is critical in reaching a diagnosis, therefore necropsies should be encouraged in any case with unexplained multifocal intracranial signs.

## PO60

### Obstructive hydrocephalus and cardiomyopathy secondary to disseminated protothecosis in a boxer dog

#### Anna Tauro^1^; John Macri^1^; Chris Gaudette^1^; Christopher L. Mariani^1^; Bonnie Brenseke^2^

**Keyword:** Prototheca, dog, heart, brain, Panfungal PCR, formalin‐fixed paraffin‐embedded tissues, cytology, histology, culture, MRI.

##### ^1^North Carolina State University; ^2^Campbell University

Canine protothecosis is a rare disease caused by saprophytic unicellular achlorophyllous aerobic algae that are ubiquitous in the environment. We report a novel case of neurological and cardiological manifestations associated with disseminated protothecosis. An adult spayed female Boxer dog was presented with a one‐week history of anorexia, progressive central vestibular signs, and a grade III/VI systolic heart murmur. Magnetic resonance imaging revealed obstructive hydrocephalus at the level of the mesencephalic aqueduct, while echocardiography and elevated troponin levels suggested an infiltrative cardiomyopathy. No obvious cause was identified. Cerebrospinal fluid collection was not performed due to associated procedural risks. Despite receiving symptomatic treatment and maintaining stability for 3 weeks, the dog eventually suffered cardiorespiratory arrest. Postmortem examination revealed disseminated protothecosis, with algae detected in heart, brain, ileum, and (medial thigh) skin. The death of this animal was attributed to the severity of disease in the vital organs of brain and heart. Panfungal PCR on formalin‐fixed paraffin‐embedded heart tissue failed to identify the specific Prototheca species, however, histomorphology, to include the finding of PAS‐positive staining with diastase‐resistant endospores, was consistent with Prototheca spp. We recommend that in cases where the cause of obstructive hydrocephalus is unclear, especially when cerebrospinal fluid collection is not feasible, a comprehensive diagnostic method should be implemented. This includes meticulous investigations to identify infected tissues, followed by sampling and performing cytology/histology and culture tests to confirm the presence of the algal organism. Early diagnosis may allow early treatment, although long‐term prognosis remains largely unfavorable due to the absence of effective treatments.

## PO61

### Paraparesis due to Leishmania Spp. in a domestic shorthaired cat

#### David Martínez Jiménez^1^; Alba Farré Mariné^1^; Alejandro Luján Feliu‐Pascual^1^; Pablo Reguera Burgos^1^

**Keyword:** Leishmania spp, Neurologic signs, Leishmania spp in cat

##### ^1^Aúna Especialidades Veterinarias IVC Evidensia

Leishmaniasis is caused by the protozoan Leishmania spp., extensively described in dogs and, to a lesser extent, in cats. Although uncommon, neurological signs have been associated with leishmaniasis in dogs. However, to the authors' knowledge, neurological dysfunction directly attributable to leishmaniasis in cats has not been reported.

An 8‐year‐old, male, castrated, domestic shorthaired cat was presented with acute progressive non‐ ambulatory paraparesis, reduced pelvic limb reflexes and severe lumbar discomfort for 1 week, localizing the lesion to the L4‐S3 spinal cord segments. The cat had been diagnosed with leishmaniasis with cytology and PCR 2 years earlier and treated with meglumine antimoniate and allopurinol. At the time of evaluation, the cat was still on allopurinol. Blood test results showed mild lymphocytosis and hyperglobulinemia, FeLV antigen, negative 2 years earlier, resulted positive. A myelo‐computed tomography showed no abnormalities.

The cerebrospinal fluid (CSF) revealed lymphocytic pleocytosis (16 WBC/μL) and positive Pandy reaction. Leishmania's PCR in the CSF was positive (52 parasite/mL), leading to the diagnosis of meningomyelitis due to Leishmania spp. Treatment with meglumine antimoniate, allopurinol, prednisolone and pregabalin initially achieved ambulatory paraparesis. However, deterioration 1 month later led to euthanasia 70 days after the diagnosis due to lack of improvement despite mycophenolate mofetil, cyclosporine and cytarabine.

Although rare, infectious meningomyelitis secondary to Leishmania spp. should be considered a differential diagnosis in cats living in an endemic area. In our case, there was lack of long‐term response despite aggressive treatment. The role of FeLV in the disease recurrence is uncertain.

## PO62

### Diagnostic work‐up and treatment of a meningoencephalitis secondary to a rhinosinusitis and naso‐ethmoidal meningoencephalocele infection with aspergillosis in a dog

#### Michelle Hermans^1^; Sanne Thijs^1^; Kenny Bossens^1^; Kaatje Kromhout^2^; Joris De Swert^3^

**Keyword:** aspergillosis, meningoeccephalocele, meningoencephalitis, dog

##### ^1^Nesto Veterinary Referral Center Orion, Herentals Belgium; ^2^Kromhout Medical Imaging, Turnhout, Belgium; ^3^Anicura Veterinary Referral Center De Ark, Westmeerbeek, Belgium

Encephalocele/meningocele/meningoencephalocele is a congenital disorder with cerebral tissue and/or meninges bulging through a skull defect. It is often recognized as a soft swelling outside the skull. This disorder is considered rare in veterinary medicine. Nasal encephalocoeles are less easily recognized as they are hidden by the sinus and nasal bone structures.

A 2‐year old crossbreed, adopted from Italy, was presented with a history of sneezing and nasal discharge from the right nostril and epileptic seizures for 6 months. Antimicrobial and phenobarbital treatment was started, but only partial improvement in seizure frequency was seen.

Clinical exam showed swelling, depigmentation, and serohemorrhagic discharge from the right nostril. Neurological exam showed obtundation as only abnormality.

CT and MRI showed the presence of a right sinonasal pathology with an ‘empty nose’ aspect and a right naso‐ethmoidal meningoencephalocele. A widening of cerebral sulci and gyri, enlarged right lateral ventricle and midline shift were suggestive for white matter loss secondary to a possible secondary meningoencephalitis. Rhinoscopy showed fungal plaques and turbinate destruction. Biopsies confirmed an Aspergillus Fumigatus infection.

Endoscopic debridement of the sinonasal structures was performed, combined with systemic itraconazole therapy for 8 weeks and phenobarbital therapy optimalization. A full recovery of the patient was seen without relapse of clinical signs since initiation of treatment 4 months ago.

To our knowledge, this is the first case report describing a rhinogenic A. Fumigatus meningoencephalitis and naso‐ethmoidal meningoencephalocele infection successfully treated with a combination of an endoscopic debridement and systemic itraconazole therapy.

## PO63

### Meningeal solitary fibrous tumor (hemangiopericytoma) in a dog

#### Alejandro Comesaña^1^; Jaume Alomar^2^; Martí Pumarola^3^; Cristian De La Fuente^1^; Sonia Añor^1^

**Keyword:** solitary fibrous tumor, hemangiopericytoma, spinal cord, myelopathy

##### ^1^Fundació Hospital Clínic Veterinari, Universitat Autònoma de Barcelona, Bellaterra, Barcelona, Spain; ^2^Servei de Diagnòstic de Patologia Veterinària, Universitat Autònoma de Barcelona (UAB), Bellaterra, Catalunya, Spain.; ^3^Unitat de Patologia Murina i Comparada, Departament de Medicina i Cirurgia Animals, Facultat de Veterinària, Universitat Autònoma de Barcelona, Bellaterra, Barcelona, Spain

Spinal diseases are a frequent chief complaint in clinical practice. Neoplasms affecting both the spinal cord and adjacent structures are a frequent cause of myelopathy. A 6‐year‐old, male, intact French Bulldog was presented for a 2‐month history of progressive ambulatory tetraparesis and occasional cervical pain. Neurological examination was consistent with a right C1‐C5 myelopathy. MRI of the cervical spine revealed a rounded intradural‐extramedullary (“golf‐tee sign”) mass located in the right dorso‐cranial aspect of the vertebral body of C2 in the vertebral canal. The mass was hyperintense on T2W images, isointense on T1W images, and enhanced strongly after contrast administration. CSF analysis showed albuminocytological dissociation. The mass was surgically excised and histopathological examination demonstrated a neoplastic vascular proliferation within a fibrous‐ myxoid stroma. Immunohistochemical staining revealed strong positivity of neoplastic staghorn pattern cells for SMA and minimal positivity for desmin. The interstitial fibrous‐myxoid stroma was positive for vimentin, PGP 9.5 and Sox10. The basal membrane and endothelial cells were positive for laminin and CD31, respectively. Immunostaining for EMA was negative. Based on the data from the histological and immunohistochemical study, a diagnosis of meningeal intradural solitary fibrous tumor was made according to the WHO 2021 nomenclature (formerly called hemangiopericytoma). The patient improved neurologically after surgery and no signs of relapse were noted during a follow‐ up performed a year later. The findings in this case are unique and had not been previously reported in dogs in this location.

## PO64

### Atypical steroid‐responsive cranial polyneuropathy and meningitis with concurrent fever of unknown origin and bilateral retinal detachment in a dog

#### Raquel Salip^1^; Sue Manning^2^; Francisco Gómez^2^; Christoforos Posporis^1^; Patricia Álvarez^1^

**Keyword:** steroid‐responsive, polyneuropathy, meningitis, fever of unknown origin, retinal detachmeng

##### ^1^Neurology and Neurosurgery Service, Pride Veterinary Referrals, IVC Evidensia group, Derby, United Kingdom; ^2^Ophthalmology Service, Pride Veterinary Referrals, IVC Evidensia group, Derby, United Kingdom

This case report aims to present the unusual manifestation of a complex presumed immune‐mediated neurological and ophthalmological syndrome in a dog.

A 7‐year‐old male Airedale Terrier presented with a one‐week history of fever, right head tilt, non‐ ambulatory vestibular ataxia, bilateral keratoconjunctivitis sicca, bilateral xeromycteria, bilateral facial paralysis, loss of corneal sensation, and absent oculocephalic reflex. Blindness developed due to bilateral retinal detachment.

Blood analysis revealed elevated C‐reactive protein (CRP) and moderate leucocytosis (neutrophilia and monocytosis). Toxoplasma gondii and Neospora caninum serologies were negative. Magnetic resonance imaging scan of the head showed bilateral otitis media‐externa, reactive lymphadenopathy (confirmed by cytology) and focal myositis of the left temporalis muscle. Cerebrospinal fluid analysis revealed moderate mixed pleocytosis, with 60% large mononuclear cells. Treatment comprised a tapering immunosuppressive protocol of prednisolone, a short course of broad‐spectrum antibiotics and topical ophthalmic treatment.

Corneal ulceration developed as a consequence of the combined neurological deficits and subsequently perforated in the left eye, requiring enucleation 1 month after discharge. Histopathology was inconclusive. At the 2‐month follow‐up, the patient was visually and neurologically normal apart from a mild right head tilt. CRP was normal. Thirteen months post‐diagnosis, the patient was found deceased in the garden with no previous signs of relapse, and medication had been discontinued 4 months earlier. No post‐mortem examination was conducted.

This case highlights a complex neurological and ophthalmological manifestation in a dog, distinct from other known immune‐mediated diseases. Further research is needed to identify the cause of similar complex neuro‐ophthalmic syndromes in veterinary patients.

## PO65

### Peripheral primitive neuroectodermal tumor at the level of the caudal medulla oblongata

#### Inmaculada Serrano Puerto^1^; Jessica Molín Molina^2^; Alba Farré Mariné^1^; Emma Hidalgo Crespo^1^; Alejandro Luján Feliu‐Pascual^1^

**Keyword:** PNET, tumor, dog, myelencephalon

##### ^1^Aúna Especialidades Veterinarias; ^2^Serra Húnter Fellow, Department of Animal Science, School of Agrifood and Forestry Engineering and Veterinary Medicine, Universitat de Lleida

Peripheral primitive neuroectodermal tumors (pPNET) are rare neuroendocrine neoplasias defined as embryonal undifferentiated tumors of neural crest origin arising in the peripheral nervous system.

A 10‐year‐old intact male Spanish water dog was referred with a 3‐month history of left hemiparesis, aphonia and coughing. Propioceptive ataxia of all four limbs with left side falls and moderate left pelvic limb postural deficits neurolocalized to the caudal medulla oblongata and C1‐C5 spinal cord segments.

A 16‐slides computed tomography (CT) using soft tissue algorithm combined with lumbar myelography revealed a well‐defined isoattenuating extra‐axial, intradural‐extramedullary lesion, markedly compressive in the caudal medulla oblongata and C1 spinal cord segment, at the level of the foramen magnum, causing widening of the left jugular foramen. The lesion homogeneously enhanced after iodinated intravenous contrast. CFS analysis showed no abnormalities. A presumptive diagnosis of neoplasia (meningioma, MPNST, histiocytic sarcoma, neuroendocrine tumor) was made.

Complete macroscopic resection through a left suboccipital approach was achieved, showing no evidence of contrast‐enhancing mass at the post‐surgical CT. Despite recovering from anesthesia, the patient developed aspiration pneumonia 24 h post‐surgery and was euthanized due to suspected sepsis and lack of response to treatment. The histopathology and immunohistochemistry revealed a pPNET.

To the authors' knowledge, this is the first report of an intracranial pPNET in the caudal medulla oblongata, treated with complete macroscopic removal, although with an unsatisfactory outcome. pPNET should be considered a differential diagnosis of cranial nerve dysfunction in the caudal medulla oblongata, with surgery as a possible therapeutic option.

## PO66

### Clinical experience with the intraparenchymal intracranial pressure monitoring codman microsensor skull bolt kit in one dog and two cats undergoing craniectomy

#### Cristiano Bendinelli^2^; Marianna D'angelo^2^; Paolo Gritti^1^; Valentina Ciampi^3^; Viola Bigazzi^4^; Rocco Lombardo^2^

**Keyword:** intracranial pressure monitoring, craniectomy, Codman Microsensor Transducer

##### ^1^Papa Giovanni XXIII Hospital, Bergamo, Italy.; ^2^ NVA Neurologi Veterinari Associati, Small Animali Neurology Referrall Center, Private Practice, Milano, Italy.; ^3^San Francesco Veterinary Hospital, Private Practice, Milano, Italy; ^4^University Teaching Veterinary Hospital, Parma, Italy

Objective Intracranial Pressure (ICP) postoperative monitoring with Codman MicroSensor Transducer (CMST) in patients undergoing craniectomy for brain tumor removal.

Methods One dog (CASE 1) and two cats (CASE 2 and 3) undergoing craniectomy for tumor removal received application of an intraparenchymal cerebral transducer (CMST) to monitor ICP in the postoperative period. The transducer was applied 3 cm from the surgery area with Codman Skull Bolt Kit. During the hospitalization the patients were sedated with dexmedetomidine continue infusion and received clinical and multiparametric monitoring including median arterial pressure (MAP) and ICP measurement.

Cerebral perfusion pressure values were calculated (CPP = MAP‐ICP). If the ICP increased over 20 mmHg for more than 45 min, 3 mL kg‐1 NaCl 7.5% was administered. After 24 h the transducer was removed, and a magnetic resonance imaging study was performed.

Results In CASE 1 the averages of values were: ICP 9.7 mmHg, MAP 76.9 mmHg and CPP 67.7 mmHg, dexmedetomidine infusion 1.8 mcg kg^−1^ h^−1^. The ICP increased at 20 mmHg for more than 1 h so a bolus of 3 mL kg^−1^ NaCl 7.5% was administered. In CASE 2 the averages were: ICP 4.7 mmHg, MAP 78.8 mmHg, CPP 74.2 mmHg, dexmedetomidine infusion 2 mcg kg^−1^ h^−1^. In CASE 3 the averages were: ICP 4.6 mmHg, MAP 90.9 mmHg, CPP 86.3 mmHg and dexmedetomidine infusion 1.5 mcg kg^−1^ h^−1^.

Conclusions The CMST device is useful to monitor postoperative ICP in patients undergoing craniectomy. The CMST consents to individuate ICP increases before the onset of clinical signs or Cushing's reflex onset.

## PO67

### Prevalence of gastrointestinal signs in dogs diagnosed with steroid responsive meningitis‐arteritis in dogs

#### Ana Oliveira^1^; Megan Madden^1^; Kiterie Faller^1^

**Keyword:** SRMA.

##### ^1^Hospital for Small Animals, The University of Edinburgh

**Introduction:** Steroid Responsive Meningitis‐Arteritis (SRMA) is an immune‐mediated inflammatory disorder that affects the leptomeninges and associated arteries, and shows a positive clinical response to corticosteroids. Associations between SRMA and immune‐mediated polyarthritis (IMPA) have been established, supporting the premise that SRMA is a systemic inflammatory disorder. Anecdotally, dogs with SRMA often develop gastrointestinal signs before or during hospitalization.

**Purpose:**

The primary aim of this study is to determine the prevalence of gastrointestinal signs in dogs with SRMA.

Methods: Retrospective case‐control study. Inclusion criteria were: dogs referred to the practice between 2018 and 2023, with a confirmed diagnosis of SRMA and with complete medical records. Control group includes dogs of similar age (less than two‐years old) that were hospitalized within the same week and with similar hospitalization length and who had not been specifically referred for gastrointestinal signs. Presence of gastrointestinal signs was determined from the medical records and compared between groups.

**Results:** Fifty dogs were included in each group. Gastrointestinal signs were more common in SRMA group when compared to the controls (*P* < 0.0005). Within the SRMA group, gastrointestinal signs were more likely to be present before starting corticosteroids (*P* = 0.003). The prevalence of vomiting (*P* = 0.001) and diarrhea (*P* = 0.028) was significantly higher in SRMA when compared to controls, but no significant difference in frequency of regurgitation (*P* = 0.558) was found.

**Conclusion:** Preliminary results suggest that dogs with SRMA frequently present gastrointestinal signs. Further research is required to exclude confounding variables and determine if inflammatory gastrointestinal disease is present in this population of patients.

## PO68

### Deep partial laminectomy, durotomy, rhizotomy, focal myelotomy and duroplasty for surgical excision of a peripheral nerve sheath tumor in a cat

#### Javier Espinosa^1^; Tomás Basto^1^; David Walker^2^; Christoforos Posporis^1^; Juanjo Minguez^1^

**Keyword:** durotomy, rhizotomy, laminectomy, pnst, myelotomy, duroplasty, neurosurgery

##### ^1^Pride Veterinary Referrals; ^2^Veterinary Pathology Group (VPG)

**Introduction:** Information regarding the surgical treatment and outcome of PNST in cats is scarce. The objective of this study is to report the surgical treatment and outcome of a cat with a benign PNST affecting the cranial thoracic spinal cord.

**Methods:** An 8‐year‐old DSH cat presented with a 3‐week history of non‐painful progressive left‐sided ambulatory paraparesis. MRI showed a compressive left‐sided mass at T1–T2 extending through the corresponding foramen. A modified paramedian approach to the vertebral column followed by a left T1–T2 unilateral deep dorsal laminectomy with subsequent durotomy was performed, and the mass was resected successfully. Rhizotomy of T1 dorsal and ventral spinal nerve roots and focal dorsolateral myelotomy was also required and were followed by duroplasty. Transient postoperative neurological deterioration was observed, including left‐sided Horner syndrome. Histopathology and immunohistochemistry confirmed a benign PNST.

**Results:** Forty‐eight hours after surgery, the cat showed a gradual but significant improvement. A neurological examination 1‐month after surgery showed further neurological recovery with resolution of Horner syndrome. Follow‐up at 3 months revealed no neurological abnormalities.

**Conclusion:** This case report describes the surgical treatment and short‐term outcome of a cat with a compressive benign PNST affecting the cranial thoracic spinal cord. It also provides evidence that T1 rhizotomy and/or focal dorsolateral myelotomy did not lead to permanent Horner syndrome in this case. Surgery should be considered for diagnostic and decompressive/cytoreductive purposes but its impact on the long‐term outcome of PNST in cats requires further investigation.

## PO69

### Use of human intravenous immunoglobulin for refractory limbic encephalitis in a cat

#### Adriana Kaczmarska^1^; Rodrigo Gutierrez‐Quintana^1^; Catherine Stalin^1^; Ana Cloquell Miro^1^; Margaux Kuijlaars^1^; Sophie Binks^2^

**Keyword:** seizures, limbic encephalitis, cat, human intravenous immunoglobulin

##### ^1^Division of Small Animal Clinical Sciences, School of Biodiversity, One Health and Veterinary Medicine, College of Medical, Veterinary and Life Sciences, University of Glasgow, Glasgow, United Kingdom; ^2^Oxford Autoimmune Neurology Group, Nuffield Department of Clinical Neurosciences, Oxford, United Kingdom; ^3^Department of Neurology, Oxford University Hospitals NHS Foundation Trust, Oxford, United Kingdom

Autoimmune limbic encephalitis (LE) associated with antibodies to leucine‐rich glioma‐inactivated 1 (LGI1) has been increasingly recognized in cats. There are no standardized optimal therapeutic protocols, and prognosis varies depending on treatment response.

A 14‐year‐old, male neutered cat presented with a 72‐hour history of behavioral changes, aggression, suspected hallucinations, and focal cluster seizures with orofacial involvement progressing to refractory status epilepticus. Routine blood work, infectious diseases serology and cerebrospinal fluid (CSF) analysis were normal except from mild hyponatraemia (142 mmol/L, reference: >145) and mild hypokalaemia (3.1 mmol/L, reference: >3.5). MRI showed subtle T2W hyperintensity of both hippocampi. Thoracic radiographs and abdominal ultrasound did not reveal any primary or metastatic neoplastic disease. The cat tested positive for serum feline‐specific LGI1‐antibodies (titre 1:320) (negative in CSF) (research assay; Royal Veterinary College ethics URN 2020 1957‐2).

The cat was treated with immune‐suppressive doses of dexamethasone, cytarabine continuous rate infusion (CRI), phenobarbital, levetiracetam, midazolam CRI, ketamine and alfaxalone CRI. Given the lack of significant improvement treatment with human intravenous immunoglobulin (IVIG) was administered (0.75 g/kg/day infusion over 10 h). The cat's condition started stabilizing 4 days post‐treatment and he was discharged after 11 days being neurologically normal and receiving prednisolone and phenobarbital.

In human medicine, IVIG and plasma exchange are effective first‐line immunomodulatory LE treatments, leading to faster clinical and cognitive recovery. Here, we report the first use of human IVIG in a cat with LE, which tolerated the treatment without apparent side effects. In conclusion, human IVIG shows promise in treating cats with refractory LE.

## PO70

### Treatment and outcome of three dachshunds with acute intradural—Intramedular disc herniation

#### Nuria Delgado Lucena^1^; Cristina Ortiz Pérez‐Hita^2^; Jose Luis Pedrero Bautista^1^; Sergio Moya García^1^

**Keyword:** INTRADULAR‐INTRAMEDULAR DISC HERNIATION, DACHSHUNDS

##### ^1^Hospital veterinario Bluecare Partners; ^2^Centro Veterinario Almanzora

Dachshunds are chondrodystrophic breeds highly predisposed to suffer compressive myelopathies due to disc extrusion. Their genetics and conformation make them susceptible to this degenerative disease. In this study, three female Dachshunds, aged 3–5 years, presented to the neurology department with clinical signs of spinal pain, paraparesis, and in one case, paraplegia without pain sensation and incontinence. The neurologic examen located the lesion in a T3‐L3 myelopathy, with acute disc extrusion as the primary differential diagnosis.

CT scans revealed Hansen Type I disc herniations, showing 85% to 90% compression: two cases in the T11‐T12 intervertebral space and one in T13‐L1. The CT images showed extradural and hyperattenuating material with suspected intradural‐intramedullary invasion. In one case, MRI also showed severely compressive material.

Surgery involved hemilaminectomy extending cranially and caudally to the affected spaces in all cases. A smaller amount of material was removed than indicated by the CT scans. Intraoperative fluoroscopy confirmed the surgical site. In one case, a mini‐hemilaminectomy was performed on the opposite side, where no extruded material was found. Post‐surgical CT scans showed persistent extruded material. No durotomies were performed.

Within 3 months, all dogs were fully ambulatory and continent, without spinal pain. Follow‐up was conducted by the physiotherapy team. The authors suspect that the surgical success was solely due to the decompression component. Further studies are needed to evaluate the benefits of durotomy in cases of suspected intradural material.

## PO71

### Gut feelings: Gastrointestinal signs and their outcome in french bulldogs undergoing spinal surgery

#### Michelle Du Toit^1^; Luca Motta^1^

**Keyword:** Intervertebral disc disease, French Bulldog, Gastrointestinal complications.

##### ^1^Northwest Veterinary Specialists

The French bulldog (FBD) is a brachycephalic breed prone to several neurological conditions, of which intervertebral disc herniation (IVDH) is considerably prevalent. Gastrointestinal (GI) disease is a reported complication in dogs surgically treated for IVDH.

The objective of this study was to describe GI signs and their outcome in FBDs surgically treated for IVDH.

Data regarding the GI signs (vomiting, diarrhea and regurgitation), their frequency and short‐term outcome in FBDs surgically treated for IVDH (cervical, thoracolumbar or lumbar) between January 2017 and April 2023 were obtained from medical records at one institution. Categorical variables were compared using Fisher exact tests, and ordinal/continuous data between categorical groups using Kruskal‐Wallis or Mann–Whitney tests.

Ninety‐seven FBDs were included for analysis. GI signs occurred in 74/97 (76.29%) FBDs while hospitalized, with 33.78% and 66.22% developing GI signs pre‐ and post‐operatively, respectively. FBDs that developed GI signs had a mean of 4.9 episodes. Diarrhea was the most common GI sign encountered (51/74) compared to regurgitation (38/74) and vomiting (22/74). Resolution of GI signs occurred within a mean of 2.23 days. Mean duration of hospitalization post‐surgery was 4.57 days in FBDs that developed GI signs versus 3.74 days in FBDs that did not (*P* = 0.033). Anesthesia length was associated with developing GI signs (*P* = 0.037). Neurological severity, neuroanatomical localisation and surgical procedure were not associated with development of GI signs (*P* = 0.42, *P* = 0.794 and *P* = 1, respectively).

In conclusion, GI signs were commonly encountered in FBDs surgically treated for IVDH and associated with length of anesthesia and prolonged hospitalization.

## PO72

### Surgical treatment of spinal stenosis causing neurological signs in an aldabra tortoise (*Aldabrachelys gigantea*) through carapacial osteotomy and hemilaminectomy

#### Viviana Rojas^1^; Tom Dutton^2^; Paul Freeman^1^

**Keyword:** Giant Tortoise, Spinal Compression, Neurosurgery, Chelonians.

##### ^1^Department of Veterinary Medicine, University of Cambridge, UK; ^2^Great Western Exotics Referral Centre, Swindon, UK

An approximately 40‐year‐old male Aldabra Tortoise (Aldabrachelys gigantea) was presented with 6 months history of paraparesis progressing to non‐ambulatory status. The tortoise was zoo kept and weighed 130 Kg. No additional clinical signs were reported and no concerns in the rest of the group. CT examination revealed moderate scoliosis of thoracic vertebral column with marked vertebral canal stenosis centred on T3. Multimodal anesthesia via dexmedetomidine, ketamine and propofol plus maintenance with propofol and isofluorane enabled access to the vertebral column via a 20 cm left sided square carapacial ostetomy. Lung lobe retraction facilitated identification of the affected vertebrae and an extensive left sided hemilaminectomy to decompress the bilaterally flattened region of spinal cord. The excised portion of carapace was replaced and secured with Kooliner. Recovery from anesthesia occurred uneventfully over the next 2 days, and improvement of the gait was reported over the following 4 months. Postoperatively the patient developed penile prolapse and despite reduction and suture correction, amputation of the penis was required. There was subsequent deterioration in gait and recurrence of moderate paraparesis, and 12 months post‐operatively the patient died. Clinicopathological findings suggested an active systemic inflammatory response to an infection. At the site of laminectomy there was complete bony regrowth and recurrence of spinal cord compression. To the knowledge of the authors there is only one previous report of surgical management for scoliosis inducing spinal cord compression in a giant tortoise without follow up. This case includes peri and postoperative care, anesthesia protocol, follow up and postmortem examination.

## PO73

### Lipid storage myopathy in a dog with trismus and generalized myopathy

#### Hamish Glover^1^; Veronica Gonzalo‐Nadal^1^; Rodrigo Gutierrez‐Quintana^2^; Catherine Stalin^2^; Diane Shelton^3^

**Keyword:** myopathy, trismus, canine, genetic disease.

##### ^1^Southfields Veterinary Specialists; ^2^University of Glasgow; ^3^University of California San Diego

Lipid storage myopathy (LSM) has been infrequently reported in veterinary medicine, with only around 30 cases in the literature. Myalgia, muscle weakness and atrophy are the most common clinical signs. Trismus has not been reported in dogs with LSM.

A 3‐year‐old Border Collie presented with a seven‐month history of progressive pelvic limb stiffness, lethargy, hyporexia and trismus. Physical and neurological examination revealed trismus, pyrexia, generalized myalgia and muscle atrophy over the lumbar spine, pelvic limbs and masticatory muscles. Full blood analysis, including thyroid profile, lactate serum levels, and masticatory myositis 2 M antibody titers were normal. Abdominal ultrasound and urinalysis revealed no abnormalities. MRI of the thoracolumbar spine, nerve conduction velocities and electromyographic studies were normal. Muscle biopsies obtained from gastrocnemius and masticatory muscles revealed excessive intramyofiber lipid droplets suggestive of a metabolic myopathy. Carnitine serum levels were reduced. L‐carnitine and Vitamin B complex were implemented as long‐term treatment. The dog showed clinical improvement but incomplete resolution of trismus. Sporadic myalgia was treated with short courses of meloxicam and gabapentin. A year after supplementation, carnitine serum levels were normal.

This case report presents a unique clinical presentation for a rare disease, being the first report of a dog with trismus due to LSM in the UK. Although complete cure is not achievable, the positive long‐term response of this patient to oral carnitine and vitamin B supplementation is consistent with the literature. Trismus should be considered as a clinical sign associated with LSM, alongside generalized myalgia and muscle atrophy.

## PO74

### Subarachnoid shunt placement in a dog with recurrent cervical spinal arachnoid diverticula

#### Flora Decrop^1^; Juanjo Minguez^1^; Patricia Álvarez Fernández^1^

**Keyword:** Subarachnoid shunt, Spinal arachnoid diverticula, Neurosurgery, Syringohydromyelia

##### ^1^Neurology and Neurosurgery Service, Pride Veterinary Referrals, IVC Evidensia group, Derby, United Kingdom

This case report describes the placement of a subarachnoid shunt as a novel treatment for the recurrence of cervical spinal arachnoid diverticula (SAD) in a dog.

A 3‐year‐old female spayed Pug showed recurring neurological signs 6 months post‐durectomy via dorsal laminectomy for a C2‐C3 SAD. Neurological signs included fecal and urinary incontinence and non‐painful moderate ambulatory tetraparesis, and proprioceptive ataxia. Repeat magnetic resonance imaging (MRI) scan confirmed the presence of SAD recurrence and caudal diffuse severe syringohydromyelia from C3 to T1 vertebral bodies. Conservative management was initially elected based on anti‐inflammatory dose of prednisolone, gabapentin and physiotherapy.

Revision surgery was performed 28 months after the initial operation. A C2‐C4 dorsal laminectomy depicted a fibrotic dural band firmly adhered to the pia mater at the previous surgical site. A partial durectomy removed the latter along with the underlying pia‐arachnoid adhesions. A 4 cm BD Angiocath 16GA was inserted to bypass the affected area, positioned within the subarachnoid space from C2 to C4 vertebrae. The shunt was sutured to the adjacent dura mater cranially and caudally using a finger trap suture pattern. Additionally, an artificial dura mater graft was placed covering the durectomy defect. A post‐operative MRI scan confirmed the correct shunt placement and partial improvement of the syringohydromyelia.

The patient demonstrated neurological improvement with persisting mild generalized proprioceptive ataxia and ambulatory tetraparesis, as well as complete resolution of both urinary and fecal incontinence. At the 8‐month follow‐up, the neurological examination was static, with no deterioration in clinical signs.

## PO75

### Post parvoviral polyneuropathy in 3 puppies resembling acute polyradiculoneurities

#### Carmen Stan^1^; Fabio Stabile^2^; Anamaria Coelho^3^; Jessica Bacon^2^; Susan Halstead^4^; Clare Rusbridge^2^; Mark Lowrie^1^

**Keyword:** parvovirus, polyneuropathy, acute polyradiculoneurities

##### ^1^Movement Referrals, Preston Broke, United Kingdom; ^2^Wear Referrals Veterinary Specialists, Linnaeus Veterinary Limited, Stockton‐on‐Tees, United Kingdom; ^3^Wear Referrals Veterinary Specialists, Linnaeus Veterinary Limited, Stockton‐on‐Tees, United Kingdom; ^4^Neuroimmunology Laboratory, School of Immunology and Infection, University of Glasgow, Glasgow, United Kingdom

Molecular mimicry is hypothesized to play a key role in the pathogenesis of acute polyradiculoneuritis (APRN), similar to Guillain‐Barré syndrome (GBS) in humans. GBS has been linked to *Campylobacter jejuni*, cytomegalovirus, and Parvovirus B19. In dogs the cause of APRN is often unknown, although recent vaccination or infection with *Campylobacter jejuni* and *Clostridium perfringens* from raw poultry diet has been documented. Anti‐GM2 and anti‐GalNAc‐GD1a IgG anti‐glycolipid antibodies are potential serum biomarkers for APRN.

Eight 2.5‐months‐old sprocker spaniel littermates and a 3‐month‐old female cockapoo presented with severe gastrointestinal signs and were diagnosed with Parvovirus infection. Six littermates died, while 2 pups (one male, one female) and the cockapoo recovered. Seven days later, the male puppy developed pelvic limb weakness progressing to flaccid tetraparesis over 24 h. The following 2 weeks, contracture of both forelimbs developed. Both female pups, 10 days after recovering from illness, developed neuromuscular signs which deteriorated for 9 days before showing improvement. They exhibited a short‐strided choppy gait with kyphosis and the cockapoo had dysphonia. Both gradually recovered over the next 4 months.

Plasma from the two littermates tested negative for anti‐ganglioside antibodies 1 and 3 months after onset of clinical signs.

Treatment included supportive care. The littermates received also a 2‐week course of anti‐ inflammatory prednisolone, and methocarbamol. The female puppy improved within 24 h of starting prednisolone, but the male was euthanised due to poor quality of life.

This study highlights the potential for a new canine Parvovirus variant predisposing dogs to neuromuscular signs after gastrointestinal illness.

## PO76

### Disseminated angiostrongylosis causing paradoxical vestibular disease with larva found in csf in a middle‐aged boxer dog

#### Clémence Berthomé^1^; Hadrien Frankar^1^; Laurent Cauzinille^1^; Hugo Kaufmann^1^

**Keyword:** angiostrongylosis, paradoxical vestibular syndrome, brain hemmorrhage, qPCR.

##### ^1^Centre Hospitalier Vétérinaire Frégis, Neurology service

Angiostrongylosis caused by *Angiostrongylus vasorum* is a more and more described cause of cerebral or spinal disease in dog, caused either by the presence of the parasite in the nervous system or by hemorrhages caused by severe coagulation defects associated with A. vasorum infection. A 2‐year‐old entire female Boxer dog was presented for acute onset of vestibular disease, in a context of chronic cough. Physical examination revealed only mild crackles at pulmonary auscultation. Neurological examination concluded to a severe right central vestibular syndrome. Hematology showed mild thrombocytopenia (111 000/uL) and neutrophilic (11,89×10^9^/L) leukocytosis (14.5×10^9^/L). Magnetic resonance imaging (MRI) of the brain revealed an acute hemorrhage at left pontocerebellar angle and signs of meningoencephalitis in frontal lobes. Cerebrospinal fluid (CSF) punction was performed. Purulent tracheal secretions were present at extubation, and larvae consistent with *A. vasorum* were identified on cytology. CSF analysis showed increased proteins, red blood cells count and neutrophilic pleocytosis. A larva was also seen on CSF. Quantitative polymerase chain reaction (qPCR) for *A. vasorum* on secretions and CSF was positive. Prothrombin time (PT) was superior to 100 s (normal: 11–17 s).

Treatment with fenbendazole, dexamethasone and tranexamic acid was initiated. The dog was transferred the day after diagnosis to the intensive care unit because of respiratory deterioration and eventually died because of respiratory arrest.

This is a first description of paradoxical vestibular disease supported by MRI‐observable lesions caused by *A. vasorum*. It emphasizes the fact that the parasite can be detected in CSF and by qPCR on CSF which has been rarely described.

## PO77

### Otogenic meningoencephalitis caused by streptococcus canis and sarocladium strictum in a dog

#### Aurora Cocchetto^1^; Anastasios Athanasiou^1^; Erica Fiorentino^1^; Luca Magna^2^; Marika Menchetti^1^

**Keyword:** SAROCLADIUM STRICTUM, STREPTOCOCCUS CANIS, OTOGENIC MENINGOENCEPHALITIS

##### ^1^Neurology and Neurosurgery Division, San Marco Veterinary Clinic, Viale dell'Industria 3, 35 030 Veggiano, Italy; ^2^Pathology Division, San Marco Veterinary Clinic, Viale dell'Industria 3, 35 030 Veggiano, Italy

Bacterial otogenic meningoencephalitis is an inflammation of the meninges and adjacent neuroparenchima caused by a bacterial infection spreading from an otitis media‐interna (OMI). The diagnosis is based on clinical presentation, blood tests, magnetic resonance imaging (MRI), and cerebrospinal fluid (CSF) analysis. Treatment involves antibiotic therapy with or without total ear canal ablation and lateral bulla osteotomy. Pathogens isolation is rarely successful. There are currently no reported cases of concomitant bacterial and fungal otogenic meningoencephalitis.

An 11‐year‐old English bulldog was referred for an acute onset of progressive difficulty walking with the hind‐limbs, loss of balance and left head tilt. The physical examination revealed mild hyperthermia. The neurological examination was consistent with a left central vestibular syndrome. The MRI showed bilateral OMI, worse on the left side, with adjacent meningeal contrast enhancement. The cytology of the CSF revealed a severe neutrophilic pleocytosis and the presence of cocci and fungi. The CSF culture was positive for Streptococcus canis, and the CSF PCR for fungi identified Sarocladium strictum. The dog was given a combination of marbofloxacin, clindamycin, and itraconazole and although there was initial improvement, the dog's neurological condition worsened and the owners elected euthanasia.

In human medicine, only a few cases of neurological disease caused by the opportunistic phytopathogen Sarocladium strictum have been described, mostly affecting immunocompromised patients. To the author's knowledge, this is the first veterinary report of concomitant bacterial and fungal meningoencephalitis and the first to identify Sarocladium strictum as a pathogen causing meningoencephalitis.

## PO78

### Short term outcome of a surgically treated spinal embryonal tumor in a dog

#### Ozgur Ulas Agdaci^1^; Alessia Silvia Colverde^1^

**Keyword:** Embryonal tumor, Peripheral neuroectodermal tumor, pNET, Glioblastoma, Nephroblastoma.

##### ^1^Wear Referrals

Spinal embryonal tumors, formerly known as peripheral neuroectodermal tumors (pNET), are rare types of neoplasia that originate from the neural crest.

To our knowledge, this is the first case in veterinary literature where the surgical removal and the post‐operative outcome has been described.

A 3‐year‐old, 18 kg female neutered cross breed, was presented for 1 month history of lethargy, neck pain, and abnormal gait. The neurological examination showed a wide based stance on all four limbs, low head carriage and thoracolumbar kyphosis, mild pelvic limb ataxia and paraparesis on the forelimbs, more prominent on left side. The neuro localization was C1‐C5 myelopathy lateralized to the left side. The MRI of the cervical spine was performed and an intramedullary, well defined, mixed signal, contrast enhancing mass was found between C1‐C2 spinal segment.

A left sided dorsal laminectomy, durotomy and myelotomy were performed, and the mass was macroscopically removed from inside the ependymal canal. The surgery was uneventful, and the patient was discharged 3 days after the surgery. The neurological examination at the time of the discharge showed improvement of the gait, with only mild four limb ataxia and left hemiparesis, and delayed proprioception on the left thoracic and pelvic limbs.

At the 2 months follow up, patient was considered neurologically normal, with only mild left torticollis, and the recheck MRI showed no relapse of the neoplasia.

In conclusion, surgical removal of the spinal embryonal tumors can be considered to achieve a short‐ term improvement of the quality of life in these patients.

## PO79

### Multifocal discospondylitis and osteomyelitis due to salmonella species in a cat

#### Despoina Douralidou^1^; Laura Muñiz^2^; Miguel Solano^3^; Lorenzo Mari^1^

**Keyword:** Salmonella spp., feline discospondylitis, ostomyelitis

##### ^1^Neurology department, The Ralph Veterinary Referral Centre, Marlow, UK; ^2^Diagnostic Imaging department, The Ralph Veterinary Referral Centre, Marlow, UK; ^3^Orthopedic department, The Ralph Veterinary Referral Centre, Marlow, UK

Feline discospondylitis is a rare condition, mostly caused by *E. coli* and *Staphylococcus* spp. A 1‐year‐ old male‐neutered Savannah cat presented with chronic recurrent lethargy, stiffness, right pelvic limb lameness and spinal hyperaesthesia. Eight months prior referral, he was treated with prednisolone, remdesivir and one‐week‐course amoxicillin/clavulanic‐acid and marbofloxacin for suspected feline‐ infectious‐peritonitis. Multiple recurrences were then treated with 1‐week‐courses of amoxicillin/clavulanic‐acid. Neurological examination did not reveal further findings. Hematology showed neutrophilia/monocytosis. Spinal/limb/thoracic radiographs revealed irregular endplates and narrowing of T12‐T13 and L7‐S1 intervertebral disc spaces, metaphyseal lesions of multiple long bones with heterogeneous medullary bone and reduced corticomedullary distinction, and a focal area of increased opacity in the left cranial lung. Abdominal ultrasound showed lymphadenopathy, reactive on cytology. FeLV/FIV/Toxoplasma serology and urine culture were negative. Blood culture was positive for *Salmonella* spp. Amoxicillin/clavulanic‐acid 20 mg/kg/q12h was started with clinical improvement. Relapse occurred after 7 months while still on treatment. Blood culture showed *Phocaeicola massilensis*, possibly contaminant. Metronidazole 11 mg/kg/q12h was added based on sensitivity, with clinical improvement but relapse after discontinuation 3 months later. Neutrophilia/monocytosis and hyperproteinemia/globulinemia were identified. Echocardiography was unremarkable. Recheck radiographs showed worsening of the osteomyelitis, but improvement of the discospondylitis. Salmonella spp. was cultured from blood and bone marrow. Marbofloxacin 4.5 mg/kg/q24h was started. At 6‐month follow‐up, for the first time, complete resolution of clinical and laboratory findings was documented. This is the first report of *Salmonella* spp. discospondylitis and osteomyelitis in a cat. Antibiotic treatment can improve the clinical signs but guidelines on duration and antibiotic of choice are needed.

## PO80

### Concurrent spontaneous compressive symptomatic pneumorrhachis and multifocal intradiscal vacuum phenomena at the site of a congenital vertebral malformation in a dog

#### Irenka Baldo Clemot^1^; Laura Muñiz Moris^2^; Lorenzo Mari^1^; Cristoforo Ricco^1^

**Keyword:** pneumorrhachis, vacuum phenomena, vertebral malformation.

##### ^1^Neurology Department, The Ralph Veterinary Referral Centre, Marlow, UK; ^2^Diagnostic Imaging Department, The Ralph Veterinary Referral Centre, Marlow, UK

Spontaneous pneumorrhachis (presence of gas in the vertebral canal) in dogs is rarely documented and its pathophysiology is debated. Concurrent spontaneous pneumorrhachis and multifocal intradiscal vacuum phenomena at the site of a spinal malformation was never reported. Reported cases with spontaneous symptomatic compressive thoracolumbar pneumorrhachis, always underwent surgical decompression.

A 6‐year‐old male‐neutered French bulldog was presented for acute, progressive, non‐painful, non‐ ambulatory paraparesis, localizing at T3‐L3 spinal cord segments. MRI revealed an extra‐dural, right dorsolateral, well‐defined, round, signal‐void area in T1W, T2W and GRE sequences at T10‐T11, moderately compressing the spinal cord. No evidence of intervertebral disc extrusion was found. Ventral and/or median vertebral body aplasia/hypoplasia were detected at multiple vertebrae between T5‐T11 causing kyphosis centred at T9‐T11, with concurrent marked spondylosis. CT confirmed the malformations and pneumorrhachis described and revealed concurrent gas content within the T9‐T10 and T10‐T11 congenitally abnormal intervertebral disc spaces. A conservative approach enabled recovery of ambulation. One‐month follow‐up revealed only mild persistent neurological deficits. Recheck CT identified full resolution of the pneumorrhachis but persistent vacuum phenomena. The dog had no relapse for 6 months, before suddenly passing away from an unrelated event.

This is the first report of concurrent spontaneous pneumorrhachis and multifocal intradiscal vacuum phenomena at the site of a congenital vertebral malformation. The pathogenesis of the multifocal gaseous intradiscal content and its migration to the epidural space is debated. A correlation with degenerative processes and/or microinstability at the malformation site is suspected. Clinical and imaging resolution of the pneumorrhachis was obtained with conservative treatment.

## PO81

### Surgical removal of intradural brainstem foreign body (microchip) with ventral basioccipital approach in a cat

#### Magdalena Olender^1^; Jérôme Couturier^1^

**Keyword:** foreign body, basioccipital, cat.

##### ^1^Veterinary Specialist Center Azurvet, Saint Laurent du Var

**Introduction:** A 2‐months old kitten was examined because of acute ataxia and depressed mental status following implantation of a pet identification microchip 2 days before.

**Methods:** Radiographs done by the referring veterinarian immediately after the implantation showed the presence of a longitudinal metallic foreign body (electronic microchip) within the cervical vertebral canal at the level of C1‐C2. A computed tomography (CT) examination was done 2 days after the incident and showed cranial migration of the microchip ventrally to the caudal brainstem.

A surgical intervention was immediately performed in order to retrieve the microchip by ventral approach to the caudal brainstem. The cat was placed in dorsal recumbency, with the head and neck in extended position. Surgical approach was similar to that described for atlantoaxial luxation with few modifications made to attain basioccipital bone. Craniectomy was made using a 2 mm burr and drilling was continued until the microchip was visualized in subdural position. A durotomy was made before removal the microchip with a fine forceps.

**Results:** The cat recovered uneventfully after the surgery and was discharged 2 days later. At two‐weeks follow up, neurological examination of the cat was normal with complete resolution of ataxia. No long‐ term complications were reported.

**Discussion:** This case report describes the unusual migration of a microchip after inadvertent implantation and its surgical removal by ventral basioccipital craniectomy.

## PO82

### Facial and vestibular neuropathy of unknown origin in 22 dogs

#### Nicola Della Camera^1^; Erica Fiorentino^1^; Marika Menchetti^1^

**Keyword:** FVNUO, dog, neuropathy

##### ^1^Neurology and Neurosurgery Division, San Marco Veterinary Clinic and Laboratory, Veggiano (PD), Italy

Facial nerve palsy (FP) is frequently observed in dogs with concurrent peripheral vestibular syndrome (PVS) due to proximity of the two nerves within the facial canal, and approximately 70% of PVS‐FP are diagnosed as idiopathic. The objective is to report the long‐term outcome.

Medical records of dogs with FVNUO confirmed by MRI and with a follow‐up of at least 6 months were retrospectively reviewed.

Twenty‐two dogs met the inclusion criteria. 6/22 patients were American Staffordshire Terrier. The mean age was 7.63 years. The left side was affected in 12/22 cases. MRI revealed a contrast enhancement of the involved nerves in all cases but one. When performed (10/22), the CSF was normal. All dogs improved without treatment after a mean time of 48 days. Recovery was complete in 9 dogs, while facial and vestibulocochlear nerve dysfunction were still present in 7 and 3 dogs respectively. Neurological relapse was observed in 6/22 dogs but in all cases a complete neurological recovery was observed.

Here we described a high prevalence of American Staffordshire Terrier. When recovery was not complete, the deficits were mainly related to dysfunction of the facial nerve. A marked improvement to complete recovery without treatment confirms the possible self‐limiting nature of the condition.

## PO83

### Alternative management of a corneal ulcer in a dog with neurogenic keratoconjunctivitis sicca

#### Francisco Gómez Villasclaras^1^; Pedro Malho^1^

**Keyword:** Neurogenic Keratoconjunctivitis Sicca, n‐butyl cyanoacrylate, tissue adhesive, corneal ulcer, NKCS, pilocarpine

##### ^1^Pride Veterinary Referrals—IVCE

A 4‐year‐old male neutered Bedlington Terrier previously diagnosed with Neurogenic Keratoconjunctivitis Sicca (NKCS) was presented with a deep stromal corneal ulcer in the right eye (OD). Clinical signs of NKCS such as ipsilateral xeromycteria and absent tear production (Schirmer tear test [STT] 0 mm/min) were present at consultation. Failure of normal tear production and ocular lubrication, crucial for ocular health, often leads to corneal ulceration in patients with NKCS. Notwithstanding treatment with pilocarpine, which acts on muscarinic M3 receptors of acinar cells, thus bypassing the conventional neural pathway, the return to normal tear production is protracted.

An alternative approach to treatment of the corneal ulcer comprised the use of n‐butyl cyanoacrylate tissue adhesive to cover the defect once the ulcerated surface was cleaned and de‐epithelised after topical anesthesia. Following a fast polymerization process, the procedure was repeated once more, filling the corneal defect. A contact bandage lens was applied, and treatment started with chloramphenicol ointment QID and frequent ocular lubrication (carbomer).

Treatment with oral pilocarpine 1% at a dose of 4 drops BID was continued, with the dosage increased by one drop in food every 3 days until tolerated. The corneal ulcer healed with minimal fibrosis, and STT was 16 mm/min by day 37 (9 pilocarpine drops BID).

Treatment with tissue adhesive may be indicated in selected cases of corneal ulceration secondary to NCKS. The procedure is minimally invasive and can be performed without the need of specialized equipment or general anesthesia, and results in reduced healing time and minimal corneal scarring.

## PO84

### Atlantoaxial synovial cyst treated by surgical fenestration in a large breed dog presenting with phantom scratching

#### Eleftheria Skovola^1^; Sara Formoso Prado^1^; Adrianna Skarbek^1^; Julius Klever^1^; Benjamin Haythornthwaite^1^; Anne‐Katherine Jasensky^1^; Joe Fenn^1^

**Keyword:** atlantoaxial cyst, phantom scratching, surgical fenestration.

##### ^1^Royal Veterinary College

**Introduction/Purpose:** Extradural synovial cysts at the atlantoaxial junction are rarely reported in dogs. A previous case report describes a Chihuahua with atlantoaxial instability and an extradural cyst causing marked neck pain without neurological deficits. Here we describe the clinical presentation, imaging findings, surgical treatment and outcome of a large breed dog diagnosed with an extradural atlantoaxial synovial cyst presenting with phantom scratching.

**Methods** Case report.

**Results:** A 9‐year‐old female neutered Labrador Retriever presented with chronic progressive episodic phantom scratching to the left side of the neck over 6 months. Neurological examination revealed mild ambulatory tetraparesis and ataxia, with intact spinal reflexes in all limbs consistent with a C1‐C5 myelopathy. Cervical MRI revealed an extradural cystic lesion causing marked spinal cord compression at the level of the C1‐C2 right neuroforamen. Cytology, obtained under ultrasonographic guidance, was consistent with synovial fluid. The patient did not respond to medical management and surgical fenestration was pursued 3 months following diagnosis. The dog recovered well with static clinical signs at discharge. The dog had only mild gait deficits but ongoing phantom scratching 3 months post‐operatively. No improvement was seen on trial treatment with maropitant.

**Discussion/Conclusion:** To the authors' knowledge, this is the first description of the surgical treatment of an atlantoaxial synovial cyst in a large breed dog. Interestingly, the dog presented with phantom scratching to the cervical region in the absence of syringomyelia. Medical and surgical treatments maintained a good quality of life, however, the phantom scratching persisted despite surgical fenestration.

## PO85

### Surgical resection of a respiratory epithelial cyst in the fourth ventricle of a dog

#### Elizabeth Mahon^1^; Virginia Crespo^1^; Kate Turner^1^; Ane Uriarte^1^; Emma Scurrel^2^; Roberto Jose‐Lopez^1^

**Keyword:** Caudal fossa cystic lesion, Sub‐occipital craniectomy, Respiratory subtype neuroenteric cyst.

##### ^1^Southfields Veterinary Specialists; ^2^Cytopath Veterinary Pathology

Surgical removal of caudal fossa cystic lesions is rarely described in the veterinary literature and with variable success. A 3‐year‐old, female neutered Cocker Spaniel presented for a four‐month history of intermittent head tilt and spontaneous yelping. Neurological examination showed left‐sided head tilt, left ventral strabismus and cervical spine discomfort. Magnetic resonance imaging (MRI) revealed a large, rounded, space‐occupying lesion in the fourth ventricle, hyperintense to gray matter with a thin hypointense halo on T2‐weighted images and FLAIR, and isointense on T1‐weighted images with marked, homogeneous peripheral contrast enhancement. It obliterated the fourth ventricle causing marked cerebellar compression, obstructive hydrocephalus and syringohydromyelia. A diagnosis of a cystic lesion in the fourth ventricle was made. A sub‐occipital craniectomy was performed and the cyst was grossly resected. Immediate postoperative MRI revealed mild pneumocephalus and marked intraventricular hemorrhage. The dog recovered from surgery showing marked cerebellovestibular signs and an absent gag reflex. It then developed aspiration pneumonia requiring mechanical ventilation and subsequently suffered four cardiopulmonary arrests associated with fluctuating signs of focal brainstem disease and intracranial hypertension. Repeat MRI 3 days after surgery revealed a large haematoma at the level of the previous cystic lesion. Revision surgery was undertaken for debulking. Despite initial mild improvement, the dog then deteriorated and was euthanised 3 days later. Histopathology showed a benign non‐keratinising epithelial cyst associated with chronic hemorrhage, cholesterol granuloma formation and fibrosis. This was consistent with a respiratory subtype neuroenteric cyst, which has been reported only once previously in this location in a dog.

## PO86

### Using the idexx procyte Dx for automated evaluation of bovine cerebrospinal fluid

#### Sara Ferrini^1^; Giulia Cagnotti^1^; Ugo Ala^1^; Claudio Bellino^1^; Giorgia Di Muro^1^; Barbara Miniscalco^1^; Antonio D'angelo^1^; Elena Bozzetta2

**Keyword:** cerebrospinal fluid, bovine neurology, automated analysis.

##### ^1^Department of Veterinary Sciences—University of Turin (Italy); ^2^Istituto Zooprofilattico Sperimentale del Piemonte Liguria e Valle d'Aosta (Italy)

**Introduction/Purpose:** Cerebrospinal fluid (CSF) analysis is crucial for diagnosing central nervous system (CNS) disorders, traditionally involving manual cell counting and microscopic cytologic evaluation. Automated CSF analysis could potentially provide much faster results, which is especially valuable in emergencies. Our study aims to evaluate the performance of the ProCyte Dx hematology analyzer for bovine CSF analysis, advancing automated diagnosis of CNS disorders.

**Methods:** We enrolled 24 healthy (Auth. N°242/2020‐PR), and 107 neurologically impaired cattle, analyzing CSF with both ProCyte Dx and traditional methods within 1 hour of collection. We evaluated the agreement between total nucleated cell count (TNCC) and differential cell count (DCC) from both methods and calculated the reference interval (RI) for normal CSF TNCC with the ProCyte Dx.

**Results:** The RI for normal CSF TNCC was <20 cells/μL. A significant correlation between the TNCC provided by the two methods was found (rho = 0.68; *P* < 0.0001). ProCyte Dx demonstrated robust diagnostic accuracy (AUC = 0.94, sensitivity = 0.84, specificity = 0.93) in detecting CSF pleocytosis. Protein concentration in CSF had the highest impact on TNCC differences between the 2 methods. DCC was feasible only for TNCC >1000 cells/μL.

**Discussion/Conclusion:** ProCyte Dx represents a potent alternative to traditional laboratory analysis, effectively detecting increased TNCC in bovine CSF in a short timeframe, with values exceeding 20 cells/μL indicating CSF pleocytosis. However, precision in TNCC measurement may be negatively affected in CSF samples with high levels of protein. Software modifications are needed for DCC.

## PO87

### Meningomyelitis presumptively associated with leishmaniasis in dogs: Clinical presentation, diagnostic findings and outcome

#### Tamara Heredia^1^; Emili Alcoverro^1^; Elisabet Domínguez^1^; Jordi Puig^1^; Christian Maeso^1^

**Keyword:** Leishmania, Meningomyelitis.

##### ^1^ AniCura Ars Veterinaria

Neurological manifestations related to Leishmania infantum (LI) infection in dogs have been described in the veterinary literature. However, meningomyelitis associated with LI is only occasionally reported. This case series aims to describe the clinical and diagnostic findings and the outcome of six dogs with meningomyelitis presumptively associated with LI infection.

Median age was 25.5 months. Two dogs were Shepherd breeds. The presenting complaints were non‐ ambulatory tetraparesis (5/6) and non‐ambulatory paraparesis (1/6). Neurological localisations were C1‐ C5 (1/6), C6‐T2 (4/6), and L4‐S3 (1/6) spinal cord segments. There was hyperthermia in 3/6 patients. Non‐ regenerative anemia and hypergammaglobulinaemia were present in dogs 4/6 and 6/6, respectively. All dogs had serological results compatible with LI infection. MRI revealed extensive diffuse T2W hyperintense intramedullary lesions without contrast enhancement (5/6), and intralesional signal void on T2*W sequence with diffuse post‐contrast enhancement (1/6). CSF analysis (performed in 4/6 dogs) revealed mononuclear pleocytosis and increased protein concentration. PCR on CSF for Toxoplasma gondii, Neospora canis, canine distemper virus, and LI were negative (4/4). Treatment with meglumine antimoniate, allopurinol, and tapering anti‐inflammatory doses of corticosteroids was administered in all cases. There was a complete recovery in all dogs, and there were no reports of any relapses. Median follow‐up was 45 months.

This case series describes a possible predilection of LI infection for the cervical spinal cord with an excellent outcome. Further studies with more cases are necessary to better describe and understand meningomyelitis associated with leishmaniasis in dogs.

## PO88

### Histopathological confirmation of septic emboli in a dog with streptococcal meningoencephalomyelitis

#### Irenka Baldo Clemot^1^; Lorena Martinez Anton^1^; Chiara Briola^2^; Carlo Bianco^3^; Catherine Ennett^3^

**Keyword:** Septic emboli, bacterial meningitis, Streptococcus, meningoencephalitis, meningoencephalomyelitis.

##### ^1^Neurology Department, The Ralph Veterinary Referral Centre, Marlow, UK; ^2^Diagnostic Imaging Department, The Ralph Veterinary Referral Centre, Marlow, UK; ^3^School of Veterinary Medicine and Science, University of Nottingham, LE12 5RD, UK

Bacterial meningoencephalomyelitis is an inflammation of the meninges and neuroparenchyma secondary to a bacterial infection. Streptococcal meningitis results from direct extension from the sinuses, middle ear, or from bacteraemia.

A 2‐year‐old female‐neutered cross‐breed dog presented for acute onset of lethargy and paraparesis that progressed to paraplegia and pyrexia within 2 days. Neurological examination was consistent with a multifocal lesion including brainstem, cervical and thoracolumbar spinal cord segments. MRI of brain, cervical and thoracolumbar spine revealed thickening and contrast enhancement of the cerebral leptomeninges and cranial cervical spine meninges, diffuse T2W and STIR intramedullary hyperintensity from L2 to L7 in association with suspected multifocal subdural empyema, and diffuse T2W and STIR paravertebral hyperintensity. The MRI findings were compatible with cerebral leptomeningoencephalitis, cervical and thoracolumbar meningomyelitis, multifocal subdural empyema and paravertebral myositis. CSF analysis documented neutrophilic pleocytosis and cultured a moderate growth of Streptococcus canis. The patient developed signs of brain death and was euthanised after 24‐h of mechanical ventilation without improvement. Histopathology of the cervical spinal cord and brain was suggestive of epidural bacterial infection with suppurative inflammation, and multifocal bacterial emboli within the cerebral vascular bed. Within the brain parenchyma, some vessels were almost obliterated by numerous colonies of coccoid gram‐positive bacteria, with no associated inflammatory influx. No comorbidities or underlying conditions were identified.

Although streptococcal infection has been associated with toxic shock syndrome, no signs of organ failure were present. This is the first case confirmed by histopathology of multiple bacterial emboli associated to brain death in a dog.

## PO89

### Detection of bacterial pathogens in bovine cerebrospinal fluid using 16S RRNA gene RT‐QPCR to improve diagnosis of central nervous system infection

#### Sara Ferrini^1^; Patrizia Nebbia^1^; Ugo Ala^1^; Elena Biasibetti^2^; Giulia Cagnotti^1^; Giorgia Di Muro^1^; Elena Grego^1^; Barbara Iulini^2^; Maria Cristina Stella^1^; Antonio D'angelo^1^

**Keyword:** bacterial meningitis, cerebrospinal fluid, bovine neurology, RT qPCR, 16S rRNA gene.

##### ^1^Department of Veterinary Sciences—University of Turin (Italy); ^2^Istituto Zooprofilattico Sperimentale del Piemonte Liguria e Valle d'Aosta (Italy)

**Introduction/Purpose:** Bacterial meningoencephalitis/myelitis (BME/M) in cattle causes significant economic and welfare issues and can be caused by various bacterial agents. Cerebrospinal fluid (CSF) bacterial culture often yields false negatives and has a long turnaround time. RT‐qPCR allows faster diagnostic results and detection of unculturable bacteria. This study evaluated RT‐qPCR targeting the bacterial 16S rRNA gene in CSF samples, as an alternative method to detect bacterial pathogens causing BME/M in cattle.

**Methods:** We enrolled 15 cattle with BME/M and 15 healthy (H) cattle (Auth. N°242/2020‐PR), performing RT‐ qPCR targeting the 16S rRNA gene on CSF DNA. H cattle and blank controls (BL) were included to account for environmental and technical sample contamination. The ΔΔCq method compared the amount of the 16S gene between BME/M and H cattle, after normalizing by subtracting the 16S signal from BL. Bacterial load was estimated by comparing *Cq* values to an *Escherichia coli* DNA standard curve.

**Results:** Our analysis revealed that 16S RT‐qPCR effectively detected bacteria in case of BME/M when the bacterial count exceeded 1500 bacteria/mL per sample, as in 7/15 cattle with BME/M. Below this threshold, the 16S signal was indistinguishable from that observed in H cattle.

**Discussion/Conclusion:** Our study indicates that 16S RT‐qPCR can detect bacterial presence in a proportion of cases of BME/M. However, low bacterial levels may result in signal similar to the background due to technical noise of the procedure and sample contamination. Including BL and H subjects in the study design is crucial for establishing reliable diagnostic thresholds.

## PO90

### Long‐term follow‐up of 35 dogs with meningoencephalitis of unknown origin treated with a combination of cytosine arabinoside and glucocorticosteroids

#### Jan Waelkens^1^; Sofie Batthi^1^; Kimberley Stee^1^; Ine Cornelis^1^

**Keyword:** Meningoencephalitis of unknown origin, Cytosine arabinoside.

##### ^1^Small Animal Department, Faculty of Veterinary Medicine, Ghent University, Belgium

**Background:** Meningoencephalitis of unknown origin (MUO) is a well‐known idiopathic immune‐mediated CNS inflammatory disease in dogs regularly diagnosed in a neurology referral center. Glucocorticosteroids are the mainstay of treatment, commonly combined with cytosine arabinoside (CA).

**Methods:** This retrospective study evaluated the outcome of a 69‐week treatment protocol using glucocorticosteroids combined with CA (two initial CRI's followed by recurrent SC injections) in dogs diagnosed with MUO. Thirty‐five dogs were followed up for at least 96 weeks. The outcome parameters were treatment success, relapse and death at three time points after diagnosis: 25 weeks, 69 weeks and 96 weeks.

**Results:** 62.9% of dogs with MUO survived the 96‐week period. Overall, 42.9% of dogs survived 96 weeks without signs of relapse (treatment success), 40% of dogs relapsed during the 96‐week period of which 20% died. The remaining 17.1% of dogs that died did so within the first 7 days after diagnosis. Success and relapse rates were respectively 65.7% and 17.1% at 25 weeks, 48.6% and 34.3% at 69 weeks and 42.9% and 40.0% at 96 weeks. The majority of the dogs that experienced relapses in this study (86%) did so during the treatment protocol. However, two dogs (14%) relapsed during the first 6 months after discontinuation of treatment.

**Conclusion:** The treatment protocol used in this study does not demonstrate superiority over earlier studies using a comparable treatment protocol. This study emphasizes the need for long‐term follow‐up to detect potential relapses after treatment discontinuation.

## PO91

### Vascular complications in a young golden retriever with steroid‐responsive meningitis‐arteritis

#### Alexander Paul Sablotni Aguilera^1^; Anja‐Maria Schmidt^2^; Birgit Parzefall^1^

**Keyword:** SRMA, Vasculitis, Hematoma, Arteritis.

##### ^1^Small Animal Clinic Oberhaching, Oberhaching, Germany; ^2^ IDEXX GmbH/Vet Med Labor GmbH, Kornwestheim, Germany

Steroid‐responsive meningitis‐arteritis (SRMA) is a common neurological and systemic disease in dogs, particularly affecting medium to large breeds. This case report details vascular complications in a one‐ year‐old intact female Golden Retriever diagnosed with SRMA.

The patient presented to the neurology service because of lethargy, fever, scleral hemorrhage, and suspected cervical pain 3 days after the removal of a large intrathoracic mass via thoracotomy. Neurological examination revealed significant cervical, thoracic, and lumbar spinal pain. Blood tests showed leucocytosis, elevated C‐reactive protein levels, and normal blood coagulation.

Magnetic Resonance Imaging of the spine suggested multifocal epidural hemorrhage. Cerebrospinal fluid analysis showed xanthochromia, increased protein, and neutrophilic pleocytosis with negative results for infectious diseases, indicating SRMA. Histology of the thoracic mass revealed a haematoma. The patient recovered within days of starting corticosteroid treatment and multimodal pain therapy. Steroids were tapered over a course of 7 months, with no relapse 16 months after diagnosis.

SRMA occasionally is associated with hemorrhage because of the underlying arteritis. As it represents a systemic disease, the bleeds could be located anywhere in the body and have previously been described in the brain, spinal cord, vertebral canal and in the thorax. Former case reports describe presumed mediastinal haematomas based on imaging.

This is the first case report with a histologically confirmed thoracic haematoma in a dog with SRMA which occurred simultaneously with scleral and epidural hemorrhage indicating an underlying systemic vasculitis. Therefore, SRMA should be considered as differential diagnosis for young febrile dogs with spinal pain and bleeding.

## PO92

### Subarachnoid diverticulum associated with a high‐velocity low‐ volume disc extrusion in a Staffordshire bull terrier

#### Francisca Couto^1^; Joana Tabanez^1^; Jeremy Rose^1^; Lorna Arrol^1^

**Keyword:** subarachnoid diverticulum, spinal cord, high‐velocity low‐volume disc extrusion.

##### ^1^Lumbry Park Veterinary Specialists

Spinal subarachnoid diverticulum (SAD) is a dilation of the subarachnoid space filled by cerebrospinal fluid (CSF), often reported in the thoracolumbar area, and typically associated with progressive, non‐ painful, pelvic limb ataxia and urinary/fecal incontinence. The underlying etiology of SAD formation in dogs is unknown but intervertebral disc disease, vertebral anomalies, spinal cord trauma and inflammatory cord disease are proposed as possible causes of acquired SAD; however most cases are not imaged prior to the development of SAD.

An 8‐year‐old, male, Staffordshire Bull Terrier was presented for investigation of 1 year of progressive, non‐painful, pelvic limb ataxia and urinary and fecal incontinence. 2 years prior to presentation this dog was diagnosed by MRI with a T13‐L1 low volume high velocity disc extrusion from which it had recovered over 2 months. MRI and CT scans at our institution revealed spinal cord compression of the cord at the level of the T12‐L1 vertebrae suspected to be caused by a combination of meningeal adhesions causing irregular dilation of the dorsal subarachnoid space and changes compatible with a syrinx at the level of the T11‐12 vertebrae. These changes were suspected to be related with the previous diagnosis of low volume high velocity disc extrusion at T13‐L1. Surgical management was declined and the dog was initially treated medically prednisolone but, due to side effects, this was changed to non‐ steroidal anti‐inflammatory and physiotherapy. This dog continued to deteriorate, eventually becoming non‐ambulatory paraplegic and was euthanised 18 months after diagnosis.

## PO93

### Case report: Diagnosis and surgical management of a type IVB dermoid sinus in the parietal and occipital bones in a young cat

#### Sophie Fish^1^; Anna Adrian^4^; Kaspar Matiasek^3^; Lara Matiasek^2^

**Keyword:** Type IVb Dermoid Sinus, Brain surgery, Cat.

##### ^1^Sophie E Fish, Small Animal Referral Center, Frontier Veterinary Specialists, Munich, Germany; ^2^Lara A Matiasek, Small Animal Clinic, Centre for Clinical Veterinary Medicine, LMU Munich; ^3^Kaspar Matiasek, Section of Clinical and Comparative Neuropathology, Centre for Clinical Veterinary Medicine, LMU Munich; ^4^Anna Adrian, Small Animal Referral Center, Frontier Veterinary Specialists, Munich, Germany

**Introduction/Purpose:** A dermoid sinus is a congenital, tubular skin defect due to incomplete separation of the skin and nervous system during embryonic development. Commonly seen in Rhodesian ridgebacks, it is rare in cats and usually affects the spine. It has occasionally been described intranasally or superficially on the head. This report details a unique case of a cat with dermoid sinus on the head with intracranial extension, emphasizing the diagnostic and surgical challenges involved.

**Methods:** A 1.5‐year‐old female British Shorthair cat presented with a previously superficially resected recurring bump on her head (showing abnormal pattern of hair growth and small ulcerations), focal scratching and proprioceptive deficits. MRI and CT were performed to assess extension for surgical planning.

**Results:** Imaging revealed an extraaxial mass extending in a tubular fashion through a midline defect in the parietal and occipital bones, caudally along the malformed tentorium. There was mild focal cerebellar compression and suspected secondary meningitis, slightly more right‐sided. The surgical procedure involved an ellipsoid incision and slight enlargement of the bone defect via combined caudotentorial and small right‐sided rostrotentorial craniectomy. Careful dissection along the lesion borders and durectomy of focally attached thickened meninges allowed complete resection. No post‐surgical complications or further neurological deficits occurred. Histopathological examination confirmed a dermoid sinus. Eight months follow‐up revealed no recurrence.

**Discussion/Conclusion:** This case report describes, to the authors knowledge, the first type IVb dermoid sinus in a cat including successful surgical treatment, contributing valuable insights into diagnosis and management of similar cases in veterinary medicine.

## PO94

### Sterile multifocal pyogranulomatous osteomyelitis in a dog

#### Henry Mendo^1^; Alicja Czerwiec^1^; Erica Fiorentino^1^; Arianna Costa^2^; Marika Menchetti^1^

**Keyword:** osteomyelitis, corticosteroids, immunomediated.

##### ^1^Neurology and Neurosurgery Division, San Marco Veterinary Clinic and Laboratory, Veggiano (PD), Italy; ^2^Diagnostic Imaging and Interventional Radiology, San Marco Veterinary Clinic and Laboratory, Veggiano (PD), Italy

Osteomyelitis is an inflammatory condition of bone and bone marrow mostly secondary to an infectious agent. Typical radiological features include monostotic lesion with osteolysis and/or osteoproliferation and periosteal reaction. In human medicine, chronic recurrent multifocal osteomyelitis (CRMO) is a sterile bone disease affecting mainly children. To our knowledge, there is only one case report of a dog with multifocal osteomyelitis resembling CRMO and here we describe a second case with similar findings.

A 5‐year‐old, female Jack Russel Terrier was presented with a chronic history of lethargy, fever and weight loss. The neurological examination revealed lower‐head carriage, mild paraparesis, diffuse hyperesthesia at the thoraco‐lumbar vertebral column and reluctance to cervical extension. Blood work showed a marked inflammatory pattern. Whole body CT showed multifocal osteolysis at the thoraco‐lumbo‐sacral vertebrae and bone marrow osteopathy in multiple appendicular bones. An incisional biopsy of the wing of the ileum was performed. Histopathological analysis revealed pyogranulomatous osteomyelitis. No etiologic agent was identified. The diagnosis was consistent with sterile multifocal pyogranulomatous osteomyelitis (SMPO). Steroid therapy was initiated at immunosuppressive dose and the dog showed clinical and hematological improvement. During a telephone follow‐up 1 month later the owner reported significant improvement.

SMPO is a challenging disease with guarded prognosis according to scarce literature. To establish a diagnosis, histopathologic analysis and exclusion of etiologic agents are mandatory. SMPO should be included as differential diagnosis in multifocal osteomyelitis with similar CT features.

## PO95

### Long‐term clinical and magnetic resonance imaging follow‐up of a third‐ventricule tumor treated by a single palliative ventriculoperitoneal shunt in a 2‐year‐old dog

#### Juliette Dailly^1^; Maud Debreuque^1^

**Keyword:** Intraventricular mass, ventriculoperitoneal shunt, hydrocephalus

##### ^1^Centre hospitalier vétérinaire Saint Martin

The third ventricle can be affected by various tumors, mainly choroid plexus carcinoma. Clinical signs are commonly caused by secondary internal hydrocephalus. Treatment options are limited, and no gold‐standard has been described. Radiotherapy associated with ventriculoperitoneal shunts have been reported to decrease tumor volume and increase survival time.

A 2‐year‐old Staffordshire Bull Terrier is presented with chronic non‐intentional generalized tremors (1 year) and recent behavior changes with hypovigilance (1 month). MRI revealed a cystic/hemorrhagic globular mass in the 3rd ventricle with strong contrast enhancement and secondary obstructive internal hydrocephalus. Anti‐inflammatory corticosteroid therapy combined with unilateral ventriculoperitoneal shunt rapidly improved neurological signs.

MRI follow‐up at 5 and 11 months post‐operative showed resolution of ventriculomegaly and progressive mass reduction (50% after 11 months). The dog showed no clinical sign of recurrence 15 months after surgery.

We described a unique case of a dog affected by a 3rd ventricular lesion with atypical chronic clinical presentation, treated with a single ventriculoperitoneal shunting without clinical recurrence over 15 months. Long term follow‐up is rare in cases palliatively treated with ventriculoperitoneal shunting and mass reduction without antitumor treatment has never been described.

Age, chronic clinical history at presentation, long‐term follow‐up with unilateral ventriculoperitoneal shunt and decrease in tumor size, lead us to suspect rare benign ventricular lesion (papilloma, ependymoma, neurocytoma or vascular hamartoma) although initial MRI characteristics may be compatible with choroid plexus carcinoma. Ventriculoperitoneal shunt may be an effective option for ventricular lesion with secondary hydrocephalus, without definitive histopathological diagnosis or specific antitumor treatment.

## PO96

### Novel cause of trismus in a cat: ‘LUNG‐DIGIT SYNDROME’ with locked jaw

#### Jad Abouzeid^1^; Friederike Rau^1^; Adrian Philbey^2^; Veronica Nadal^1^

**Keyword:** Trismus, exophthalmos, metastatic neoplasia, lung digit syndrome, Feline pulmonary carcinoma, locked jaw

##### ^1^Southfields Veterinary Specialists, Linnaeus Ltd, UK; ^2^Easter Bush Pathology, Royal (Dick) School of Veterinary Studies, University of Edinburgh, Edinburgh

The inability to open the mouth, known as trismus, has been reported in dogs and cats with disorders of the temporomandibular joint (TMJ), masticatory muscles and trigeminal nerve. Trauma and masticatory muscle myositis (MMM) are the most common causes but MMM has been reported rarely in cats. Feline pulmonary carcinomas are a relatively uncommon primary neoplasm recognized for having unusual patterns of metastasis with ‘lung‐digit syndrome’ being a well‐documented presentation.

A 9‐year‐old, male neutered domestic short hair cat presented with a progressive two‐week history of trismus, left sided exophthalmos and left thoracic limb lameness. Physical examination revealed complete trismus which persisted under general anesthesia. Computed tomography revealed a primary pulmonary mass, multiple masses in the thoracic and abdominal cavities, distant skeletal muscle masses, an osteolytic lesion of the first digit of the left thoracic limb and marked bilaterally symmetrical contrast enhancement of the masticatory muscles. Post‐mortem examination revealed primary pulmonary carcinoma of the left caudal lung lobe, with extensive metastasis including infiltration and fibrosis of the pterygoid muscles. Histopathology revealed similar pleomorphic columnar to polygonal cells with a high mitotic count in primary and metastatic areas, including the pterygoid muscles, without infiltrative inflammatory cells.

Although primary neoplasias affecting the TMJ has been reported previously, there are no reports of metastasis affecting the masticatory muscles in cats prior to this case. Therefore, metastatic cell infiltration within the masticatory muscles should be considered as a differential diagnosis in cats presenting with trismus and concurrent multiple mass lesions compatible with metastatic neoplasia.

## PO97

### Neuroaxonal dystrophy in Shiba Inu; Clinical neurological presentation and pathological findings

#### Anne Muhle^1^; Anna‐Mariam Kiviranta^1^; Julija Tamosauskaite^2^; Pernilla Syrjä^1^

**Keyword:** dog, brain, neuroaxonal dystrophy.

##### ^1^Faculty of Veterinary Medicine University of Helsinki Finland; ^2^Animal Hospital Aisti Finland

**Introduction:** Investigating familial neurological disease using canine littermates can detect novel diseases, elucidate comparative pathogenetic mechanisms, and enable genetic discovery. A prerequisite is the verification of a shared phenotype among affected dogs. This poster describes the clinical and pathological findings in two Shiba Inu littermates affected by neuroaxonal dystrophy.

**Methods:** Two male Shiba Inu littermates were presented at the age of 6 and 12 months because of progressive neurological signs, starting as puppy. The dogs underwent clinical and neurological examination, brain MRI and genetic testing for gangliosidosis. Both dogs underwent postmortem examination including staining for iron, polysaccharides, autophagic (p62, LC3), structural (neurofilament) and mitochondrial markers (VDAC/porin).

**Results:** Both dogs showed gait changes: hind limb ataxia, swaying, falling towards both sides, front limb hypermetria, tendency to circle right. Behavioral changes with aggressiveness, biting fits of flanks and hind limbs, as well as episodes of dyskinesia: ventroflexion, lifting and cramping of front limb, tremors occurred. The menace responses and postural reactions were decreased. The neurological localization was multifocal. MRI showed corpus callosum thinning. Both dogs were negative for gangliosidosis. Postmortem examination revealed variable sized spheroids containing mitochondria and neurofilaments throughout the gray and white matter of the CNS, milder in the PNS. No iron accumulation was present, and spheroids were PAS negative.

**Discussion:** neuroaxonal dystrophy is described in various breeds, however not previously in the.

Shiba Inu. Occurrence in two littermates may indicate a genetic etiology and histopathology suggest axonal dystrophy due to structural and/or axonal transport alteration.

## PO98

### Hearing assessment in pomeranian dogs with and without chiari‐like malformation based on brainstem auditory evoked response wave latencies—‐A pilot study

#### Marta Plonek^1^; Paul Mandigers^1,2^

**Keyword:** BAER, CM, wave latencies

##### ^1^ IVC Evidensia Dierenziekenhuis Arnhem; ^2^Department of Clinical Sciences, Faculty of Veterinary Medicine, Utrecht University, Utrecht, Netherlands

Chiari‐like malformation is an inherited structural disorder of the caudal cranial fossa described both in humans and animals. In veterinary medicine it is usually observed in brachycephalic and toy breeds, including the Pomeranian. It has been postulated the disrupted cerebrospinal fluid passage at the craniocervical junction in the course of CM may alter the auditory pathway, leading to increase of wave I‐V inter‐peak latencies and the latency of wave V.

This pilot study compared the brainstem auditory evoked response wave latencies in Pomeranians suffering from CM 1 with healthy same‐breed controls.

Ten dogs without history of hearing abnormalities were sedated and underwent MRI and CT imaging for CM grading and brainstem auditory evoked response (BAER) recording as part of a screening program in the breed.

Six dogs were graded as CM1 and four dogs had CM0. BAER recordings at 20–90 dB every 10 dB were recorded for the right and left ear and waves I, III and V latencies were measured. In addition, wave I–III, I–V and III–V inter‐peak latencies were recorded. No significant differences in wave latencies between the CM0 and CM1 groups were noted (*P* > 0.05).

The preliminary results show no significant influence of mild CM on BAER wave latencies in the Pomeranian. A future study including Pomeranians with CM1 as well as more advanced CM2 and a more numerous control group will help provide more definitive results.

## PO99

### Cervicothoracic kyphoscoliosis in a llama CRIA

#### Marta Plonek^1^; Koen Santifort2,^3^; Dimitry Verduijn^4^; Ewa Stanczyk^5^; Wilhelmina Bergmann^6^; Paul Mandigers^7^

##### ^1^ IVC Evidensia Dierenziekenhuis Arnhem; ^2^ IVC Evidensia Dierenziekenhuis Arnhem IVC Evidensia Dierenziekenhuis Hart van Brabant,; ^3^Department of Clinical Sciences, Faculty of Veterinary Medicine, Utrecht University, Utrecht, Netherlands; ^4^ DAP Oostland; ^5^Vet Oracle Teleradiology, Norfolk, United Kingdom; ^6^Division of Pathology, Department of Biomedical Health Sciences, Faculty of Veterinary Medicine, Utrecht University, Utrecht, Netherlands; ^7^Department of Clinical Sciences, Faculty of Veterinary Medicine, Utrecht University, 3584 CM Utrecht, The Netherlands

Vertebral malformations are rare in camelids and are reported to comprise 2% of all congenital defects. They include hemivertebrae and various forms of scoliosis and single cases have been reported to occur in the cervical, thoracic, and caudal vertebrae.

A 6‐month‐old cria presented with a scoliotic deformity of the cervicothoracic vertebral column, mild ambulatory tetraparesis, and progressive dyspnea. She was slightly shorter and thinner than other crias of the same age. There was no history of trauma and the owners reported progression of the deformity over the previous weeks. Due to the concerns about the quality of life of the cria, euthanasia was performed.

Post‐mortem CT and MRI studies revealed a complex levo‐kyphoscoliosis including a malformed lateral wedge‐shaped T6 vertebra and severe malformation and deviation of the ribcage. Histopathological assessment of the thoracic spinal cord revealed mild gliosis in gray matter, but no changes in the white matter.

Scoliosis in camelids has been described as a congenital malformation or secondary to trauma. In congenital scoliosis, malformed vertebrae cause secondary vertebral column distortion, as appreciated in this case. The present case was singular and most likely caused by a fetal environmental developmental disorder of the T6 vertebra.

## PO100

### Magnetic resonance imaging and histopathological findings of a plant foreign body in the optic nerve of a dog. A case report

#### Magdalena Maria Dyrka, Dvm Mrcvs^1^; Gawain Hammond, Ma Vetmb Mvm Certvdi Dipecvdi Fhea Mrcvs^1,2^; George Peplinski, Ma Vetmb Certvophthal Mrcvs^1^; Emma Scurrell Bvsc Dipacvp Mrcvs^3^; Stacie Aarsvold, Dvm Dacvr^2^; Maria Pasierbińska, Dvm Mrcvs^1^; Rodrigo Gutierrez‐Quintana, Mvz Mvm Dipecvn Mrcvs^1^; Adriana Kaczmarska, Dvm Mvm Dipecvn Mrcvs^1^

**Keyword:** MRI, optic nerve, foreign body, head tilt, strabismus, histopathology, enucleation, dissociated hypotropia.

##### ^1^Division of Small Animal Clinical Sciences, School of Biodiversity, One Health and Veterinary Medicine, College of Medical, Veterinary and Life Sciences, University of Glasgow, Glasgow, UK; ^2^ VetCT Teleradiology Teleconsulting, Cambridge, CB3 0FA, UK; ^3^ CytoPath Veterinary Pathology Ltd, Ledbury, Herefordshire, HR8 2YD, UK

Grass awns commonly cause morbidity in canines, affecting the auditory canal, interdigital regions, eyes, thoracic cavity, and iliopsoas muscles.

A 1‐year‐old female Labradoodle presented with acute onset lethargy, a right head tilt and right positional ventral strabismus, with bilateral enophthalmos, blepharospasm, and conjunctivitis. Clinical signs progressed to right uveitis, intra‐retinal hemorrhages, and elevated intraocular pressure, with initially preserved vision.

Magnetic resonance imaging (MRI) of the head showed T2W hypointense material within the vitreous chamber of the right globe and a small irregular T2W hypointense material within the posterior aspect of the same eye with associated retinal detachment, optic nerve thickening and contrast enhancement of the sclera, iris, and extra‐ocular muscles. These findings were compatible with an inflammatory process affecting the right eye globe and associated structures.

Cerebrospinal fluid analysis, routine blood tests and serology for infectious diseases were normal. Lack of response to antibiotics and steroids, leading to blindness, necessitated enucleation. Histopathological examination identified plant material in the vitreous chamber as well as embedded within the optic nerve head and optic nerve itself with associated suppurative endophthalmitis, retinal detachment and secondary glaucoma.

This case report describes the clinical, MRI and histopathological characteristics of a migrating foreign body (plant material) involving the optic nerve. The dog's initial atypical head posture was likely adaptive response to enhance vision and mitigate discomfort. The observed positional ventral strabismus could represent a dissociated hypotropia, indicative of ocular motility dysfunction characterized by downward excursion of a non‐fixing eye due to visual impairment.

## PO101

### Clinical features, MRI findings and outcome in dogs with non‐ traumatic intra‐cranial intra‐axial hemorrhage

#### Jiaqi Sng^1^; Flora Decrop^2^; Christoforos Posporis^2^; Ella Fitzgerald^1^; Sophie Wyatt^1^

**Keyword:** Stroke, Cerebrovascular accident, Magnetic resonance imaging.

##### ^1^Department of Veterinary Clinical Science and Services, Royal Veterinary College, University of London, Hatfield, UK; ^2^Neurology and Neurosurgery service, Pride Veterinary Referrals, IVC Evidensia Group, Derby, United Kingdom

**Introduction/purpose:** Canine non‐traumatic intra‐cranial‐intra‐axial hemorrhage (IAH) is caused by rupture of intra‐cranial blood vessels and can be classified as primary (unknown cause/concurrent hypertension) or secondary. The study objective was to document clinical features and diagnostic findings in canine IAH and evaluate the association between short‐term and long‐term outcome.

**Methods:** A multicenter observational study was undertaken using medical records of dogs diagnosed with IAH using previously published MRI‐criteria. Data was collected regarding clinical features (ambulation, seizure occurrence, mentation status), and MRI findings (brain herniation, midline shift, lesion size and location). Dogs which had a confirmed/suspected neoplastic mass with associated hemorrhage were excluded. Short‐term and long‐term (>6 months) outcome was recorded.

**Results:** Thirty‐two dogs were included; 22 (69%) had primary and 10 (31%) had secondary IAH. The most common concurrent conditions were coagulopathy associated with angiostrongylus vasorum (2/10) and proteinuria (2/10). Twenty‐seven dogs (84%) survived to discharge. There was no significant difference in survival to discharge between primary and secondary IAH (*P* = 0.637). Obtundation (*P* = 0.355), seizures (*P* = 0.161), and non‐ambulatory status (*P* = 0.163) were not significantly associated with survival to discharge. MRI features including larger lesion size (*P* = 0.001), multifocal lesion location (*P* = 0.020), and brain herniation (*P* = 0.024) were significantly associated with non‐survival to discharge. Survival to discharge was positively associated with long‐term survival (*P* = 0.002).

**Discussion/conclusion:** The majority of dogs diagnosed with IAH have no identifiable underlying cause. Brain herniation, lesion size and lesion location are important short‐term prognostic indicators. Providing dogs survive to discharge they have a good prognosis for long‐term survival.

## PO102

### Clinical signs, magnetic resonance imaging features and surgical treatment of spinal myxoma/myxosarcoma in 4 dogs: A case series

#### Nina Schneider^1^; Marco Armellini^3^; Kaspar Matiasek^2^; Francesca Cozzi^1^; Rocco Lombardo^1^

**Keyword:** Myxoma, Spinal tumor, Spinal synovial myxoma, Dogs, MRI.

##### ^1^Clinica Neurologica Veterinaria NVA, Milan, Italy; ^2^Institute of Veterinary Pathology, Center for Clinical Veterinary Medicine, Ludwig‐Maximillians‐University Munich, Munich, Germany; ^3^Futurovet, Tolentino, Italy

**Introduction:** Synovial myxomas are histologically unique joint tumors affecting mostly limb joints in middle‐aged, large‐breed dogs. Vertebral synovial myxomas have rarely been reported in veterinary literature.

**Methods:** This retrospective case series describes clinical signs, magnetic resonance imaging (MRI) findings and surgical treatment in four dogs with histopathologically confirmed vertebral synovial myxoma or myxosarcoma.

**Results:** Dogs presented at a median age of 8 years and with a history of variable chronic progressive clinical signs including ambulatory or non‐ambulatory paraparesis in three dogs and thoracic limb lameness in one dog. MRI in all dogs revealed an extradural, multilobulated or roundish, well‐defined, markedly T2W hyperintense, T1W hypo‐isointense variable contrast‐enhancing mass lesion. In three dogs, the mass was associated with a facet joint at the level of the thoracic, lumbar or lumbosacral spine and extended into the vertebral canal. In the fourth dog, the mass was associated with the costovertebral joint at the level of T1 occupying the entry zone of the first thoracic spinal nerve without invasion into the vertebral canal. Surgical excision of the mass was performed in all dogs combined with either hemilaminectomy, dorsal laminectomy or partial costectomy depending on the location of the mass. Based on histopathology, two dogs were diagnosed with myxoma and two dogs with myxosarcoma. All dogs improved after surgery. In one dog with diagnosed myxoma, a follow‐up MRI after 2 years showed no evidence of local recurrence.

**Discussion/Conclusion:** Synovial myxomas/myxosarcomas should be included as differentials for vertebral mass lesions with described characteristic MRI features.

## PO103

### Encephalic mycobacterial pyogranuloma in a domestic ferret (*Mustela putorius* furo)

#### Ana Cloquell^1^; Jessica Molin Molina^2^; Pilar González‐Iglesias Sitges^3^

**Keyword:** Ferret, mycobacteria, pyogranuloma.

##### ^1^Small Animal Clinical Sciences, School of Biodiversity, One Health and Veterinary Medicine, College of Medical, Veterinary and Life Sciences, University of Glasgow, Glasgow, United Kingdom; ^2^Serra Húnter Fellow, Departament de Ciència Animal, Campus Agroalimentari, Forestal i Veterinari, Universitat de Lleida, Lleida, Spain; ^3^C. V. Exovet Vetersalud, Madrid, Spain

Mycobacteria are Gram‐positive, aerobic, acid‐fast, nonsporulating, facultative intracellular bacilli. Mycobacteriosis in domestic ferrets has been sporadically described, typically presenting with gastrointestinal signs or pneumonia. However, clinical signs and imaging findings associated to a nervous system involvement have not been previously reported.

A 5‐year‐old female‐neutered ferret presented with chronic ambulatory paraparesis progressing over 4 weeks to depressed mentation, ambulatory tetraparesis worse on the right side, and proprioceptive ataxia in all limbs. A left forebrain lesion was localized. Routine blood work showed mild elevation of ALP (307 U/L, reference: <120). A head CT scan revealed an intra‐axial, round (4 mm), well‐demarcated, hypoattenuating lesion in the left thalamus with marked ring enhancement, causing midline shift to the right and ventricular collapse. Cerebrospinal fluid analysis showed neutrophilic pleocytosis, but culture was negative. Differential diagnoses included inflammatory/infectious diseases and less likely neoplastic processes. Treatment with trimethoprim‐sulphonamide and steroids showed no improvement. The ferret deteriorated, showing depression, decerebellate rigidity, anisocoria, and spontaneous death 3 weeks later. Histopathology revealed a focally extensive pyogranulomatous inflammatory lesion in the thalamus and adjacent cerebral hemisphere, lateral ventricle, and subarachnoid space, with numerous Ziehl‐Neelsen positive intrahistiocytic bacilli, consistent with a mycobacterial pyogranuloma. Additional special stains and immunohistochemistry excluded fungal and coronavirus infection, respectively.

Mycobacteriosis is a serious, and progressive disease, generally presenting systemically. This case highlights central nervous system involvement in ferrets, not previously described. Prognosis for disseminated mycobacteriosis is guarded, depending on the mycobacteria type, infection extent, and severity. The zoonotic potential underscores the importance of recognizing this disease.

## PO104

### Photosensitive epilepsy in a dog: A case report of lafora disease

#### Manon Guehl^1,2^; Solene Diop^1,2^; Elsa Lyon^3^; Sabine Chahory^2^; Stéphane Blot^1,2^

**Keyword:** lafora disease, EEG, iris atrophy, light‐induced myoclonus.

##### ^1^Univ Paris Est Créteil, INSERM, IMRB, F‐94010 Créteil, France; ^2^Centre Hospitalier Universitaire Vétérinaire Animaux de Compagnie (CHUV‐AC), Ecole nationale vétérinaire d'Alfort, F‐94700 Maisons‐ Alfort, France; ^3^Univ Lyon, VetAgro Sup, Marcy‐l'Etoile, France

Lafora disease is a rare progressive myoclonic epilepsy in canines, characterized by the accumulation of Lafora bodies in neurons, leading to myoclonus, tremors, or seizures. We report the case of a 10‐year‐old spayed Chihuahua presenting with light‐induced myoclonus which appeared suddenly 6 months before the consultation and were only observed by the owner during daytime walks. The clinical evaluation and complementary tests included a detailed history, neurological and ophthalmological examinations, serum biochemistry, brain magnetic resonance imaging (MRI), lumbar puncture, electroencephalogram (EEG), and genetic testing. The differential diagnosis included acquired or genetic photosensitive myoclonus. The neurological examination revealed head and generalized myoclonus triggered by external light stimuli. Biochemical screening, brain MRI and lumbar puncture results were unremarkable. The EEG showed polyspikes occurring both simultaneously and independently of the myoclonus, increasing during intermittent photic stimulation. The ophthalmological examination revealed severe bilateral senile iris atrophy, associated with hyporeflexive pupillary reflexes causing dazzle under photopic conditions. The diagnosis of Lafora disease was confirmed by genetic analysis. In this case, the myoclonic signs of Lafora disease may have been secondarily triggered by the onset of retinal atrophy, leading to photosensitivity in the animal. This case highlights the importance of considering ocular causes in reflex epilepsies, particularly in rare neurodegenerative diseases such as Lafora disease.

## PO106

### Reliability of deep pain perception test prior to referral for dogs diagnosed with thoracolumbar disc extrusion

#### Cara‐May Bailie^1^; Filipe Maximiano Sousa^2^; Megan Madden^1^; Aran Nagendran^1^; Joao Miguel De Frias^1^

**Keyword:** Intervertebral Disc Extrusion, Nociception.

##### ^1^Hospital for Small Animals, Royal (Dick) School of Veterinary Studies, The University of Edinburgh; ^2^Veterinary Public Health Institute, Vetsuisse Faculty, University of Bern, Switzerland

**Introduction:** Evaluating deep pain perception (DPP) in paraplegic dogs with thoracolumbar intervertebral disc extrusion (TL‐IVDE) is paramount for determining recovery of ambulation.

**Purpose.**

To determine the prevalence of DPP discrepancy from general practitioner (GP) to referral clinician (RC) and clinical factors affecting reliability of this test.

**Methods:** Dogs non‐ambulatory on pelvic limbs, where DPP was performed by GP, were retrospectively selected and clustered: Group‐1 GP DPP positive + RC DPP positive and GP DPP negative + DPP negative; group‐2 GP DPP negative + RC DPP positive; group‐3 GP DPP positive + RC DPP negative; group‐4 individualized limb with DPP at GP and/or RC. Dogs without confirmed TL IVDE with advanced imaging and assessment by RC > 48 h from GP were excluded. Chondrodystrophy, body weight, body condition score, sex, age, duration of clinical signs, anxiousness, neurolocalisation, spinal shock, lateralisation and spinal hyperesthesia were compared between group‐1 and 2. Variables with *P* ≤ 0.25 in univariable logistic regression were included in the multivariable model and considered significant if *P* ≤ 0.05.

**Results:** Total 146 dogs: 107 group‐1; 21 group‐2; 3 group‐3 and 15 group‐4. Spinal hyperesthesia and duration of clinical signs were identified as risk factors. Absence of spinal hyperesthesia [OR 2.89 (1.05–7.94)] and duration of clinical signs for less than 12 h [OR 10.75 (1.13–102.3)] had higher chances of DPP test to be inaccurate.

**Conclusion:** In 14% of TL‐IVDE dogs, GP DPP assessment was misclassified negative. Peracute onset in non‐painful dogs can affect GP DPP evaluation.

## PO107

### Multifocal meningioangiomatosis in the spinal cord of a young dog

#### Hana Gunovska^1^; Sara Degl'Innocenti^2^; Mike Targett; Ines Carrera^4^; Sergio A. Gomes^1^ MRCVS; ^2^ DVM, PhD, MRCVS; ^3^ MA, VetMB, PhD, DipECVN, SFHEA, MRCVS; ^4^ DVM, MVM, PhD, DipECVDI, MRCVS

**Introduction/Purpose:** Meningioangiomatosis (MA) is an unusual proliferative disorder of the central nervous system (CNS), typically a non‐malignant, focal lesion arising within the leptomeninges and compressing/infiltrating the underlying neural‐parenchyma by leptomeningeal and meningovascular proliferation. Limited reports are available describing spinal cord involvement in young/middle aged dogs. Predilection site in dogs are the brainstem and cerebrum with lesions involving the cervical and thoracic spinal cord reported. This is the first case report of MA with a multifocal spinal cord localisation affecting the cervicothoracic, thoracic, thoracolumbar, lumbar regions.

**Methods/Results:** A 2‐year‐old male entire Labrador retriever presented for a 3‐week history of pelvic limb ataxia progressing to non‐ambulatory paraparesis. Neurological examination revealed ambulatory paraparesis, intact segmental spinal reflexes. A neurolocalisation was to the T3‐L3 spinal cord segments.

MRI of the thoracolumbar (TL) spine revealed two presumed intramedullary lesions, the largest located from the caudal aspect of T8 to the mid‐aspect of T9, and a second located over L3‐4. The lesions were both homogeneous, T2W hyperintense, T1W isointense, with intense and homogeneous contrast enhancement.

Surgical exploration was undertaken. Durotomy revealed a mass protruding from the parenchyma of the spinal cord. The mass was firmly adherent, resection was only possible by sharp dissection. Euthanasia was performed due to neurological deterioration.

Histopathology of the excised mass and necropsy of the CNS demonstrated multifocal to coalescing leptomeningeal and meningovascular proliferations consistent with MA, some of which were not visible on MRI.

**Discussion/Conclusion:** This case highlights meningioangiomatosis as a possible differential diagnosis for multifocal intramedullary masses affecting young dogs.

## PO108

### Lumbo‐sacral lipomyelomeningocele associated with urinary and fecal incontinence in an adult dachshund treated surgically

#### Ana Martinez^1^; Sam Khan^2^; Fernando Constantino‐Casas^2^; Bethany Guy^1^; Georgina Harris^1^

**Keyword:** Spinal dysraphism, Neural tube defect, Lipomyelomeningocele, Magnetic resonance imaging, Incontinence, Surgical treatment, Spina bifida.

##### ^1^The Queeńs Veterinary School Hospital, University of Cambridge; ^2^The Queen's Veterinary School Hospital, University of Cambridge

Lipomyelomeningocele is a rare type of neural tube defect associated with spina bifida occulta with herniation of neural tissue and meninges out of the defect that is also associated with a lipoma.

A 4‐year 7‐month 7.2‐kg male neutered Miniature Dachshund presented for evaluation of a 5‐month history of chronic and progressive urinary and fecal incontinence with initial response to anti‐ inflammatory non‐steroidal medication. The clinical signs progressed after discontinuation of the treatment. The dog had a previous history of caudectomy at 7 weeks old due to self‐trauma.

General physical examination was unremarkable apart from a docked tail. The findings in the neurological examination revealed reduced perineal reflex bilaterally. Neurological examination localized a lesion at the S1‐S3 spinal cord segments. Possible differential diagnoses included; anomalous (spina bifida, meningomyelocele), degenerative (intervertebral disc disease) or neoplasia. Non‐neurological causes of incontinence were considered less likely due to previous work up and neurological exam findings.

Blood test and urinalysis including culture were unremarkable. Magnetic resonance imaging revealed spina bifida with a suspected lipomyelomeningocele at L7‐S1. Surgical resection of the suspected lipomyelomeningocele via a L7‐S1 dorsal laminectomy was performed. Histopathological examination confirmed a lipomyelomeningocele. Four weeks postoperatively the dog had complete resolution of urinary and fecal incontinence. The perineal reflex remained reduced. Six months follow‐up revealed the dog's continence was sustained.

This is the first report of a lipomyelomeningocele at the lumbosacral area in a dog causing urinary and fecal incontinence treated surgically with a good outcome mid‐term.

## PO109

### Severe lumbosacral syndrome due to presumed immunomediated polymyositis and intramuscular neuritis in three dogs

#### Ilaria Tartari^1^; Tania Al Kafaji^1^; Giorgio Cancedda^2^; Antonella Gallucci^1^

**Keyword:** lumbosacral syndrome, intramuscular neuritis, polymyositis.

##### ^1^Veterinary Neurological Center “La Fenice”, Selargius (CA), Italy; ^2^Veterinary Clinic “Cancedda”, Carbonia (CI), Italy

Simultaneous myositis and neuritis are a rare condition in dogs. Here we present three dogs affected by a worsening lumbosacral syndrome due to a concurrent presumed immunomediated myositis and neuritis.

All dogs were presented for lumbosacral pain and worsening paraparesis resulting in the inability to walk on the pelvic limbs. Spinal reflexes and proprioception were decreased on the pelvic limbs. Magnetic resonance imaging showed normal spinal cord but severe bilateral muscles alterations in the lumbosacral tract. Cerebrospinal fluid was normal. Electrodiagnostics showed spontaneous muscle activity in the lumbosacral epiaxial and appendicular muscles of the pelvic limbs. Sciatic motor nerve conduction was reduced in case 1 and 2, not performed in case 3. Serology for infectious disease were negative in all dogs. In dog 1 muscle biopsies revealed multifocal interstitial lymphocytic and neutrophilic infiltrates of the muscle fibers and nerve branches. Microorganisms were not detected. In dog 2 cytological preparations showed rare lymphocytes and mature neutrophils. In dog 3 the owner declined muscle biopses. Clinical successful was achieved with immunosoppressive doses of prednisolone in all dogs, despite the persistence of slight paraparesis in dog 1 and left monoparesis in dog 3. Fully recovery was obtained in dog 2.

This case series illustrates the possibility that a polymyositis and intramuscular neuritis may cause a severe lumbosacral disease that improved clinically with glucocorticoid treatment. Thus, it should be considered as a differential diagnosis in dogs with clinical signs of lumbosacral syndrome. Muscle biopsies should be preferred to confirm the diagnosis.

## PO110

### Congenital hydrocephalus combined with colobomatous cyst, microphtalmia and orbital malformation in a young dog

#### Iris Van Soens^1^; Esther Lichtenauer^1^; Koen Santifort^1^; Kaspar Matiasek^2^; Dorien Willems^1^

**Keyword:** hydrocephalus, colobomatous cyst, MRI, post‐mortem examination.

##### ^1^Evidensia Small Animal Hospital Hart van Brabant, Waalwijk, The Netherlands; ^2^Section of Clinical & Comparative Neuropathology, Institute of Veterinary Pathology, Centre for Clinical Veterinary Medicine, Ludwig‐Maximilians‐Universität München, Munich, Germany

A colobomatous cyst is an eye malformation characterized by a cavity lined by neuroectodermal tissue that communicates with the vitreous cavity. It is suspected to result from improper fusion of embryonic fissures and is often associated with microphthalmia.

We present a case involving a 3.5‐month‐old Pomsky with combined congenital hydrocephalus and colobomatous cyst with microphthalmia. The puppy presented with acute onset non‐ambulatory tetraparesis and obtundation. Left‐sided microphthalmia with vision loss was diagnosed at 6 weeks, and by 12 weeks, the left eye bulb had enlarged. At 14 weeks, the puppy became suddenly lethargic and non‐ambulatory.

Neurological examination showed obtundation, non‐ambulatory tetraparesis with decreased proprioception in the pelvic limbs, and absent menace response, direct and indirect pupillary reflexes, and vestibulo‐ocular reflex in the left eye. A multifocal neurolocalization was suspected. Congenital malformation, immune‐mediated/infectious meningoencephalitis, or less likely metabolic disease or neoplasia were considered.

Complete blood cell count, biochemistry, and pre‐prandial bile acids were unremarkable. MRI of the head revealed severe generalized hydrocephalus with cerebellar compression and foramen magnum herniation. Furthermore, findings were consistent with left‐sided microphthalmia and a suspected colobomatous cyst.

Due to progressive clinical signs, humane euthanasia was chosen. Post‐mortem examination confirmed a bicompartimental herniation following concentric forebrain hydrocephalus, with a septum defect and ventral dislocation of the fornix with obliteration of the interventricular foramina. A dysplastic microphthalmos was observed at the wall of the colobomatous cyst occupying the entire conal compartment.

Colobomatous cyst with microphthalmia is rare in dogs and, in this case, was associated with congenital hydrocephalus.

## PO111

### Atypical clinical signs of peracute asymmetric encephalomyelopathy without signs of emphysema due to clostrial myositis in a dog

#### Jasmin N. Nessler^1^; Kristina Merhof^1^; Alexandra Schütter^1^; Ingo Gerhauser^2^; Filip Kajin^3^

**Keyword:** Clostridium, myositis.

##### ^1^Dept. of Small Animal Internal Medicine and Surgery, University of Veterinary Medicine Hannover, Foundation, Hannover, Germany; ^2^Dept. of Pathology, University of Veterinary Medicine Hannover, Foundation, Hannover, Germany; ^3^Clinic for Internal Diseases, Faculty of Veterinary Medicine, University of Zagreb, Zagreb, Croatia

Clostridium perfringens myositis is rare in dogs and mostly reported as gas gangrene with clinical signs of emphysema and rhabdomyolysis. We report a peracute asymmetric encephalomyelopathy in a dog with clostridial myositis without clear clinical signs of myositis and myonecrosis.

A 12‐year‐old male Beagle presented with peracute pain and vomiting. He had mild systemic signs including elevated body temperature (39.5°C), pink mucous membranes, and mild tachypnea. Initially, he showed neurologic signs compatible with a painful, ambulatory T3‐L3 myelopathy. Blood tests showed mildy increased liver enzyme activity and thrombocytosis, with normal creatine‐kinase activity. Within 1 day, the cardiovascular status deteriorated and signs of multifocal asymmetric encephalopathy with reduced consciousness, asymmetric menace deficits, and unilateral extensor muscle spasticity emerged. After stabilization, the dog underwent a magnetic resonance imaging examination of the whole spine and brain under general anesthesia, which revealed multifocal, partly confluent lesions in the paraspinal and shoulder muscles with ill‐defined, heterogeneous T2weighted hyperintense signal and multifocal well demarcated round areas of signal void, loss of normal muscle architecture, mild mass effect, and contrast enhancement in T1weighted images. Central nervous system (CNS) parenchyma and meninges were unremarkable.

Myositis due to gas producing bacteria was suspected. No cerebrospinal‐fluid puncture was performed to prevent CNS contamination by potential infectious agents from the musculature. The dog suffered from cardiorespiratory arrest while recovering from anesthesia. Histopathological examination of a muscle biopsy revealed hyaline muscle fiber degeneration with neutrophilic inflammation and intralesional rod‐shaped bacteria, most likely compatible with Clostridium spp.

## PO112

### Multiple spinal meningioma drop metastases 16 months after surgery of a meningioma at C1‐C2 in a Cairn Terrier

#### Theresa Kalliwoda^1^; Matiasek Kaspar^2^; Loderstedt Shenja^1^

**Keyword:** meningioma, canine.

##### ^1^Department for Small Animals, Leipzig University, Leipzig, Germany; ^2^Section of Clinical & Comparative Neuropathology, Centre for Clinical Veterinary Medicine, Ludwig‐Maximilians‐Universität München, Munich, Germany

**Introduction/Purpose:** Meningiomas are the most frequent spinal cord tumors in dogs and occur most common in the cervical spinal cord. Here we describe multiple meningioma metastases along the spinal cord.

**Methods:** An 8‐year old Cairn Terrier presented with progressive tetraparesis, underwent MRI and decompressive surgery. Control MRI was performed directly, 9‐ and 16‐months post‐surgery. The dog deteriorated and post mortem examination revealed the same type meningioma as the surgical biopsy.

**Results:** Ambulatory tetraparesis, proprioceptive deficits with normal segmental reflexes in all 4‐limbs localized to C1‐C4 spinal cord segments. MRI revealed an intradural extramedullary mass at the level of C1–C2 vertebral bodies (VB) most consistent with meningioma. Mass removal was performed via dorsal laminectomy C1‐2. Post‐surgical MRI showed a 2 mm contrast enhancing structure interpreted as tumor residual tissue. Histopathological examination classified the surgical biopsy as spinal grad II meningioma. Re‐MRI was performed 9‐months and 16‐month post‐surgery when the dog represented with increasing pelvic limb deficits. Multiple contrast enhancing extramedullary, mass lesions at C1, C2, T8‐9, T13 and L4 VB were diagnosed. Surgery was declined, the dog was euthanized and pathological examination revealed a spinal meningioma with the same morphologic features as in the previous examination. Distal CSF drop metastasis was considered most likely.

**Discussion/Conclusion:** We now report an undescribed presentation of canine spinal meningioma metastasis. CSF drop metastasis are described for intracranial meningiomas. Post‐surgical drop out metastasis should be considered as unusual complication predicting the prognosis after surgical meningioma removal.

## PO113

### Confounding parameters in neurological examination video analysis

#### Loderstedt Shenja^1^; Flegel Thomas^1^; Feist Daisy^2^

**Keyword:** Neurological examination, video analysis.

##### ^1^Department for Small Animals, Leipzig University, Leipzig, Germany; ^2^Veterinary Clinic for Small Animals Norderstedt, Norderstedt, Germany

**Introduction/Purpose:** Video analysis of clinical examination has become important in clinical work, education and teaching. Criteria like video content and technical aspects are used to evaluate quality and usefulness of video‐ material. Here we describe confounding parameters for analysis of neurological examination videos.

**Methods:** Neurological examination videos recorded between 2004 and 2014 were reviewed. Potential confounding parameters were defined and categorized in: recording conditions, recording technique, examination environment, animal, examiner. 100 examination sequences were randomized selected, cut, distributed into 8 video‐compilations and digitally provided to 48 reviewers. Each reviewer evaluated two video‐compilations and answered a questionnaire. Interobserver agreement was determined using the mean percentage agreement [r%], Fleiss‐Kappa [KF] and intraclass correlation coefficient [ICC].

**Results:** Agreement about the presence of confounding parameter [KF] was almost perfect for recording conditions [0.913–0.93] and patient parameters (urinary catheter extension [0.834]), substantial for patient parameters (leash on dog [0.776], neck collar [0.651]) and examination environment (additional person in the image [0.646]). Most interfering parameters [r%] were fast‐moving camera [18,5%], zooming during examination [15.8%] and if animal and examiner are only partially on screen [14.7%] (recording technique). The ICC on how confounding the parameter were, was best for neck collar [0.77], leash on the animal [0.741] and fast‐moving camera [0.692].

**Discussion/Conclusion:** There is a variety of potential confounding parameters potentially influencing the video analysis of examination videos. Recording technique and patient parameters appear to be the most important categories influencing video quality. Awareness of confounding parameters on video analysis is essential to provide best quality video material.

## PO114

### Cats are not small dogs—Idiopathic vestibular syndrom might differ between cats and dogs

#### Magdalena Putzer^1^; Steven De Decker^2^; Rodrigo Gutierrez Quintana^3^; Anna Morgana Mosel^1^; Holger A. Volk^1^

**Keyword:** FLAIR, Idiopathic vestibular syndrome, inner ear, magnetic resonance imaging, vestibular signs.

##### ^1^Department of Small Animal Medicine and Surgery, University of Veterinary Medicine Hannover, Hanover, Germany; ^2^Department of Clinical Science and Services, Royal Veterinary College, University of London, United Kingdom; ^3^School of Veterinary Medicine, College of Medical, Veterinary and Life Sciences, University of Glasgow, Glasgow, UK

**Hypothesis/Purpose:**

Idiopathic vestibular syndrome (IVS) in cats is a common presentation in first opinion practice. The etiology remains unknown. In dogs it had been shown that there is difference in MRI signal intensity of the inner ear between the affected and the unaffected side. The aim of this study was to investigate if this finding can also be observed in cats with IVS.

**Methods:** Cats were included that had an acute onset and clear lateralisation of vestibular signs and had undergone an MRI of the brain, with no radiologic abnormalities observed. Both inner ears were identified in the transverse T2W images, outlined and the FLAIR suppression was quantified for each region of interest (ROI) by computing the ratio of the difference in signal intensity between the T2W and T2‐FLAIR images.

**Results:** Out of the 20 cats examined, 11 exhibited a head tilt to the left side, while 9 cats showed a head tilt to the right side. In each case, a unilateral reduction in the suppression ratio was observed. In 9 out of 20 cases, the radiologically identified abnormal side did not correspond with the lateralization of the clinical signs, while in the remaining 11 out of 20 cases, it did.

**Conclusion:** Unlike in dogs with IVS, there is no clear association between the lateralisation of clinical signs and the signal intensities of the inner ear on MRI. This might indicate that the etiology of IVS might differ between cats and dogs.

## PO115

### Spinal arachnoid diverticulum presumed secondary to thoracolumbar decompressive surgery for intervertebral disc extrusion in two dogs

#### Tiffany Pereira^1^; Guillaume Marc Albertini^2^; Edward James Ives^3^; Rita Gonçalves^4^; Christoforos Posporis^5^; João Miguel De Frias^6^; Bruno André Lopes^7^

**Keyword:** Spinal arachnoid diverticulum, Thoracolumbar intervertebral disc extrusion, Decompressive surgery.

##### ^1^Southfields Veterinary Specialists; ^2^Movement Referrals; ^3^Anderson Moores Veterinary Specialists; ^4^Small Animal Teaching Hospital ‐ University of Liverpool; ^5^Pride Veterinary Referrals; ^6^The Royal (Dick) School of Veterinary Studies ‐ University of Edinburgh; ^7^Southfields Veterinary Specialists, University of Cambridge

**Introduction:** Spinal arachnoid diverticulum (SAD) describes a cerebrospinal fluid‐filled dilatation of the subarachnoid space causing spinal cord compression. They are classified as congenital or acquired but are often considered multifactorial. The association between acquired SAD following decompressive spinal surgery has been scarcely described.

**Purpose:**

To describe SAD occurring at the same level as historic decompressive surgery for intervertebral disc extrusion (IVDE) in dogs.

**Methods:** The medical records of dogs that had been diagnosed with SAD at the same intervertebral space as previous surgery for thoracolumbar IVDE were included.

Results: Two dogs from a total five recruited are described. Firstly, a six‐year‐old, female French Bulldog with a two‐week history of acute, progressive, non‐lateralised, non‐painful ambulatory paraparesis, diagnosed with a caudal tethered, dorsal midline, bilobed T12‐T13 SAD. A right‐sided hemilaminectomy for IVDE at the same intervertebral space had been performed 1 year prior. Secondly, a six‐year‐old, male neutered French Bulldog with a three‐month history of acute, non‐progressive, right‐sided, non‐ painful ambulatory paraparesis, and fecal incontinence. A caudal tethered, dorsal T13‐L1 SAD was identified at the same intervertebral space as a right‐sided hemilaminectomy for IVDE that had been performed four years earlier. Both cases underwent dorsal laminectomy at the affected site with associated durectomy and spinal stabilization. Short‐term outcome showed neurologic improvement in both dogs.

**Conclusion:** Our findings suggest that SAD can be recognized as a potential long‐term complication following surgical decompression for IVDE in dogs. We are seeking collaboration to identify further cases and understand possible risk factors for SAD following spinal surgery.

## PO116

### Lafora disease in two French bulldogs confirmed by genetic test

#### Alfredo Molero Sierra^1^; María Ortega Prieto^1^; Laura Martín Muñiz^1^

**Keyword:** Lafora disease, French Bulldog, Genetic Test, Myoclonic episodes

##### ^1^Service of Neurology and Neurosurgery of Anicura Indautxu (Bilbao), Spain

Lafora disease (LD) is an autosomal recessive genetic disorder produced by a mutation of NHLRC1 or EPM2B genes that encodes malin and laforin glycogen phosphatase. Loss of function of one of them, result in formation, precipitation, aggregation and accumulation of insoluble glycogen in the brain, leading to neurodegeneration. Clinical signs usually appear between 6 and 13 years old and mainly consist of myoclonic episodes in response to visual, auditory or sensory stimuli. The symptoms may progress to generalized seizures, vision loss, ataxia and decreased cognitive function.

Two French Bulldogs (male and female, both of 9 years old) were presented due to myoclonic episodes both triggering by visual stimuli, mainly rapid light/shadow changes. General, ophthalmologic and neurological examination were normal. Complete blood work and CSF tap were unremarkable. Brain MRI on female case revealed a thalamic lesion compatible with an old cerebrovascular accident.

Genetic DNA testing was performed for the known NHLRC1 mutation responsible to the LD and both dogs were homozygous for the disease mutation. Treatment with levetiracetam at 20 mg/kg every 8 h resulted in complete resolution of clinical signs on female case and decreased frequency and intensity of myoclonic episodes in male case.

To the authors knowledge this is the first report of LD on French Bulldogs only diagnosed by genetic test, without the histopathology requirement.

## PO117

### Intravitam diagnosis of ganglioradiculoneuritis in a dog

#### Valentina Buffagni^1^; Kaspar Matiasek^2^; Giovanni Mattioli^1^; Serena Galaverna^1^; Diletta Dell'apa^1^; Ezio Bianchi^1^

**Keyword:** dog, ganglioradiculoneuritis, spinal ganglion, electrodiagnosis.

##### ^1^Dept. Of Veterinary Science, University of Parma, Italy; ^2^Section of Clinical and Comparative Neuropathology, Ludwig‐Maximilians‐Universitaet, Munich, Germany

Canine ganglioradiculoneuritis (CG) is a rare, presumably immune‐mediated disease that causes the abrupt onset of progressive sensory signs. Definitive diagnosis of CG requires histological proof of inflammation of cranial and/or spinal nerve roots and respective ganglia. Currently, most clinicians are reluctant to procure biopsies from this region unless the lesion mimics a neoplasm. We describe a case of a dog with an intravitam diagnosis of CG.

A 9‐year‐old, female, Australian Cattle dog, was referred for abnormal gait, licking/biting and self‐ mutilation of the toes. The signs had started acutely 2 months earlier and progressed rapidly.

On neurological examination, the dog was non‐ambulatory, with absent proprioception and reduced spinal reflexes of all limbs, and hypo/anesthesia of the distal extremities and face.

Differential diagnoses for this acquired generalized (mainly sensory) neuromuscular disorder included inflammatory, toxic and metabolic diseases. CBC, serum biochemistry, thyroid panel and serology for infectious causes of polyneuropathy were unremarkable.

Electrodiagnostic tests, which included evaluation of sensory conduction after administration of neuromuscular blocking agents, confirmed the predominant involvement of sensory fibers. Muscle, nerve and spinal ganglion biopsies revealed the presence of a severe sensory lymphoplasmohistiocytic ganglioradiculitis. Treatment included prednisolone, cyclosporine, gabapentin, oclacitinib and physiotherapy. Four months after diagnosis, the dog was euthanised due to lack of improvement.

To the authors' knowledge, this is the first case report of CG with confirmed intravitam diagnosis. Biopsy of the spinal ganglion should be included in the diagnostic work‐up if CG is suspected. Early diagnosis and treatment could improve the outcome of this serious condition.

## PO118

### Intermittent vestibular episodes in a dog secondary to syringobulbia

#### Alfredo Molero Sierra^1^; Raúl Altuzarra Fernández^2^; María Ortega Prieto^1^; Laura Martín Muñiz^1^

**Keyword:** Syringobulbia, Intermittent Vestibular Episodes, Chiari‐like malformation, Syringomyelia.

##### ^1^Service of Neurology and Neurosurgery of Anicura Indautxu (Bilbao), Spain; ^2^Diagnostic Imaging Service of Anicura Indautxu (Bilbao), Spain

Syringobulbia is a pathologic condition characterized by one or more fluid‐filled cavities within the brainstem. The pathogenesis is supposed to be produced by altered cerebrospinal fluid (CSF) hydrodynamics, in fact in most cases syringobulbia is an advancement of syringomyelia craneal to the foramen magnum. Chiari‐like malformation (CLM) is also related in most dogs with syringobulbia. Previous clinical signs associated with syringobulbia include vestibular signs, hyperalgesia, sensory deficits, dysphagia and seizures. Magnetic resonance imaging (MRI) reveals a slit‐like, bulbous, and vertical linear shapes of the cavities on T2‐weighted hyperintense and T1‐weighted hypointense signals similar to the CSF.

The present case is a 5 years old miniature pinscher that suffered vestibular episode characterized by horizontal nystagmus and circling during the recovery of a general anesthesia. Subsequently, similar episodes were described monthly with rapid recovery. Physical and neurological examination were completely normal, except circling to the right in stressful situations. MRI of the head revealed a small vertical and linear fluid‐filled cavity in the medulla oblongata, slightly deviated to the right side. No other abnormalities were evident. CSF tap was normal and the dog recovered uneventfully from the general anesthesia. No treatment was started and the dog was completely normal at 6 months of follow‐up, without more vestibular episodes.

To the author's knowledge it is the first case reported with intermittent vestibular episodes and syringobulbia with no other abnormalities in the MRI such as syringomyelia, CLM or cranio‐cervical abnormalities, in contrast with the previous veterinary literature.

## PO119

### Auricular dyskinesia associated with pyometra in a dog

#### Miriam Vargas Mendoza^1^; Júlia Vilardell Rigau^1^; Jorge Pérez‐Accino Salgado^1^; Cristina Font Nonell^1^

**Keyword:** Auricular, Myoclonus, Dyskinesia, Pyometra.

##### ^1^Hospital Veterinari Canis Girona

A 9‐year‐old female entire cross‐breed dog presented for investigations of a 3‐week history of lethargy, polydipsia, polyuria and bilateral ear twitching. Physical examination revealed pyrexia (39.6°C) and continuous jerky auricular myoclonus, which appeared bilateral, variably rhythmic and often synchronous. They were no suppressible and disappeared during sleep. Otoscopic examination was unremarkable and neurological examination was normal.

Hematology showed neutrophilic leukocytosis (WBC 86.1 K/uL, reference 5.5–16.9 K/uL) with left shift neutrophilia. Serum biochemistry revealed mild hyperglycaemia and hyperglobulinemia.

Imaging was performed to investigate an inflammatory, infectious or neoplastic focus.

Thoracic radiographs were unremarkable. Abdominal ultrasound was consistent with pyometra. The patient underwent emergency ovariohysterectomy which was performed without complications and the patient recovered uneventfully. The patient received intraoperative potentiated amoxicillin and NSAIDs. The abnormal involuntary ear movements resolved 4 days after surgery with no other specific treatment.

Auricular myoclonus is a rare presentation in both human and veterinary medicine with poorly understood etiology. To our knowledge, there is only one published report where abdominal botryomycosis was suspected to be associated with involuntary ear movements in a dog. In humans, it has been reported secondary to neuroleptic drugs, focal seizures, idiopathic condition (which are mostly treated with muscle botulin toxin A injections), and streptococcal infections. It has been theorized that antibodies targeting the bacteria may cross‐react with epitopes on neurons in the basal ganglia, resulting in motor dysfunction. In our case, there were no other indicators of obvious CNS abnormalities and they disappeared after the pyometra surgery.

## PO120

### C2‐C3 intramedullary disc extrusion associated with aphonia and tetraparesis in a cat

#### Solene Diop^1,2^; Jeremy Mortier^2^; Aurore Masson^2^; Manon Guehl^1,2^; Stephane Blot^1,2^

**Keyword:** Disc extrusion, Aphonia, Feline, Cervical myelopathy.

##### ^1^Univ Paris Est Créteil, INSERM, IMRB, F‐94010 Créteil, France; ^2^Centre Hospitalier Universitaire Vétérinaire Animaux de Compagnie (CHUV‐AC), Ecole nationale vétérinaire d'Alfort, F‐94700 Maisons‐ Alfort, France

An 8‐year‐old female neutered domestic shorthair cat was presented with acute ambulatory tetraparesis. Trauma was considered unlikely, as the cat was kept indoors. Complete aphonia was also reported by the owners, demonstrated by unfruitful attempts to vocalize. Neurological examination was consistent with a C1‐C5 myelopathy, although localization to the caudal myelencephalon could not be ruled out because of the aphonia. Magnetic resonance imaging of the head and neck revealed a linear, ill‐defined, intramedullary T2W hyperintense, T1W hypointense lesion at the level of the C2‐C3 intervertebral disc space. The C2‐C3 intervertebral disc showed partial loss of the nucleus pulposus T2 hyperintensity, decreased volume and mild dorsal protrusion. Magnetic resonance imaging of the brain and visual examination of the larynx during intubation were unremarkable. Cerebrospinal fluid analysis showed marked neutrophilic pleocytosis, with multiple erythrophagocytosis figures. These findings were consistent with C2‐C3 intramedullary disc extrusion.

Medical management with an anti‐inflammatory dose of prednisolone and physiotherapy was initiated. Progressive improvement in the locomotion and the aphonia was observed, particularly within the first 2 weeks after the onset of clinical signs.

Dysphonia associated with cervical spinal cord injury has been previously described in humans and dogs. To the author's knowledge, this is the first description of cervical intramedullary disc extrusion associated with aphonia in a cat.

## PO121

### postoperative seroma after spinal surgery in dogs: Prevalence, potential risk factors, treatment and outcome

#### Ruben Jimenez Gonzalez^1^; Chris Posporis^1^; Juan José Mínguez^1^; Patricia Álvarez^1^

**Keyword:** Seroma, Paraspinal fluid, Spinal surgery, Neurosurgical complications.

##### ^1^Neurology and Neurosurgery Department, Pride Veterinary Referrals, IVC Evidensia, Derby, United Kingdom

**Introduction/Purpose:** Seroma formation is anecdotally reported following spinal surgery. The purpose of this study is to describe the prevalence of seroma in dogs following spinal surgery and to evaluate potential risk factors, treatment and outcome.

**Methods:** Medical records of dogs undergoing spinal surgery between January 2016 and June 2023 were reviewed. Cases were allocated in a control group (CG) consisting of dogs without seroma or seroma group (SG). Collected data from complete medical records included physical and neurological examinations, advanced imaging, spinal surgery reports, and details about seroma formation based on physical examination and/or fluid evaluation. Follow‐up information was documented until complete resolution of the seroma.

**Results:** 94 dogs met the inclusion criteria (SG: 47; CG: 47). The prevalence of seroma was 4.0%. Statistical analysis revealed a significant difference in neuter status (*P* = .021) and body weight (*P* = .00001) between the two groups. Seroma was more common in male intact dogs (44.7%) with a body weight.

≥15 kg (55.3%) that underwent thoracolumbar surgery (91%) and hemilaminectomy (76.6%). The median time of seroma onset after surgery was 14 days (7‐16). The median time of seroma resolution was 14.5 days (range 7–21) in 20/47 dogs with this information available. Strict rest and hot‐cold packing three times daily was the elected treatment in 66% of patients.

**Discussion/Conclusion:** The prevalence of postoperative seroma after spinal surgery is low in dogs. Potential risk factors may include neuter status, higher body weight and thoracolumbar surgery. Prognosis is favorable following conservative management.

## PO122

### Presumed primary spinal nerve root melanoma in a dog

#### Jessica Lobb^1^; Emma Scurrell^2^; James Elliott^1^; Jessica Molin^3^; Marti Pumarola^4^; Sebastien Behr^5^; Roberto Jose‐Lopez^1^

**Keyword:** Spinal nerve, Melanocytic tumor, Canine, Magnetic resonance imaging, Debulking surgery.

##### ^1^Southfields Veterinary Specialists; ^2^Cytopath Ltd; ^3^Universitat de Lleida; ^4^Universitat Autonoma de Barcelona; ^5^Willows Veterinary Centre

A 2‐year‐old female neutered Springer Spaniel was evaluated for acute onset of lower motor neuron signs to the pelvic limbs, flaccid tail paralysis without nociception and severe lumbar pain. Magnetic resonance imaging identified a dorsal extradural mass extending over L5‐6 vertebrae severely compressing the spinal cord. The mass was widening the vertebral canal and thinning L5 right lamina. Lesion signal was heterogeneously and predominantly iso‐intense to gray matter on T2‐weighted images, and markedly hyperintense on T1‐weighted images with mild heterogeneous contrast enhancement. Lumbar cerebrospinal fluid analysis revealed mild mononuclear pleocytosis with melanophages. An L5‐6 dorsal laminectomy and partial L5 right pediculectomy demonstrated a large dark‐brown mass intimately related to L5 dorsal nerve root. A rhizotomy was necessary for debulking. Histopathological and immunohistochemical analysis results (including high Melan A positivity and negative Schwann cell markers [p. 75, neurofilaments]) were consistent with malignant melanoma. Diagnosis was presumed primary nerve root melanoma based on the lack of a primary lesion on computed tomography of the thorax and abdomen, or repeat oral cavity, ophthalmic, aural, nailbeds and rectal examinations. The patient received anti‐inflammatory steroid therapy and three doses of the melanoma vaccine. A remarkable improvement of the lower motor neuron signs in the pelvic limbs and tail tone and sensation was noted until 5 weeks post‐surgery, when presenting signs started to gradually relapse. The patient was euthanised 8 weeks after diagnosis due to refractory pain and the owners declined post‐mortem examination. This is the first report of a spinal nerve root melanoma in a dog.

## PO123

### Suspected meniere's disease in a dog with repeated vestibular episodes

#### Maximilian Eugen Ladenburger^1^; Katarina Kuzek^1^; Annika Schmitz^1^; Axel Stanger^1^; Andrea Fischer1

**Keyword:** dog, vestibular syndrome, Morbus Ménière, brainstem auditory evoked response, otoacustic emissions, hearing test.

##### ^1^Small Animal Clinic, Centre for Clinical Veterinary Medicine, Ludwig‐Maximilians‐Universität München, Munich, Germany

**Introduction:** Ménière's disease is a disorder of the inner ear characterized by hearing loss, tinnitus, and two or more spontaneous episodes of vertigo with each lasting 20 min to 12 h.

**Methods:** Retrospective descriptive case report.

**Results:** A 15‐year‐old crossbreed dog presented with a history of three vestibular episodes in the last 2 weeks, each lasting 30–60 min, and a complaint of progressive hearing loss. Neurological examination revealed mild head tilt to the right, positional strabismus of the right eye and bilateral rotary nystagmus consistent with right‐sided peripheral vestibular disease. Otoscopic examination showed mild bilateral otitis externa, and thromboelastography indicated hypercoagulability. MRI showed unremarkable middle and inner ear structures, no signal abnormalities of the intra‐ labyrinthine fluids on T2‐weighted and FLAIR sequences, and incidental cerebral microbleeds. Cerebrospinal fluid analysis was unremarkable. Comprehensive hearing testing with click ABR hearing thresholds and distortion product otoacoustic emissions indicated bilateral sensorineural hearing loss with hearing thresholds >70 dB nHL (right ear) and >90 dB nHL (left ear), and preservation of high frequency DPOAE at 8 kHz on the right ear. The dog improved gradually with treatment with intravenous fluids, oral maropitant and clopidogrel. A relapse occurred after 2 years.

**Discussion:** Based on the dog's sensorineural, low frequency hearing loss and clinical presentation with repeated vestibular episodes, a suspected diagnosis of Ménière's disease was made, aligning with the diagnostic criteria for humans. However, we cannot exclude that the recurrent vestibular episodes and sensorineural hearing loss are coincidental findings rather than a singular disease process.

## PO124

### Paraplegia and ischaemic myelopathy following a lumbosacral epidural steroid injection in a dog

#### Charis Tang^1^; Chris Posporis^1^; Juan Jose Minguez^1^; Patricia Álvarez^1^

##### ^1^Neurology and Neurosurgery Service, Pride Veterinary Referrals, IVC Evidensia Group, Derby, United Kingdom

This case report describes the first known dog experiencing paraplegia and ischaemic myelopathy as a presumed secondary complication of an epidural steroid injection.

A seven‐year‐old male neutered Dalmatian underwent a lumbosacral epidural steroid injection under general anesthesia as treatment for degenerative lumbosacral stenosis at the primary vets. The neurological examination prior to the infiltration was normal. On recovery, the patient became acutely paraplegic, with tail paralysis and urinary incontinence.

Upon referral the following day, the neurological examination revealed a left‐lateralised non‐painful non‐ambulatory paraparesis, tail paralysis with absent nociception in tail and perineal region, reduced pelvic limb spinal reflexes, and absent perineal reflex. The lesion was localized to the L4 to caudal spinal cord segments. Iatrogenic injection into the spinal cord, such as traumatic haematomyelia, or compressive myelopathy was initially suspected. The MRI scan confirmed the presence of a bilateral and slightly left‐lateralised intramedullary hyperintensity extending from L3 to L5 vertebrae, predominantly affecting the gray matter, indicative of ischaemic myelopathy. The patient displayed gradual gait improvement with physical rehabilitation, yet tail nociception and bladder function had not returned at nine‐day follow‐up.

Ischaemic myelopathy following epidural or transforaminal steroid infiltration is a rare but recognized complication in human medicine. Its occurrence has never been reported in the veterinary literature until now. Though extremely rare, it is important to recognize this potential consequence. Several mechanisms have been proposed, such as direct vascular trauma, arterial spasm, and steroid embolism. The prognosis for this patient remains uncertain requiring longer follow‐up to reach more definitive conclusions.

## PO125

### Suspected anti‐ glial fibrillary acidic protein encephalitis in a 4‐year‐ old labrador retriever: A case report

#### Mercedes Aradillas Pérez^1^; Corinna I. Bien^2^; Christian G. Bien^2^; Jana Van Renen^1^; Lara A. Matiasek^1^; Andrea Fischer^1^

**Keyword:** GFAP antibodies, Autoimmunity, Limbic encephalitis, Epilepsy.

##### ^1^Clinic of Small Animal Medicine, Centre for Clinical Veterinary Medicine, Ludwig‐Maximilians‐ Universität München, Munich, Germany; ^2^Labor Krone, Salzuflen, Germany

**Introduction:** Antibodies against glial fibrillary acidic protein (GFAP) have been linked to autoimmune GFAP astrocytopathy in humans and meningoencephalitis of unknown etiology in dogs, most commonly necrotizing meningoencephalitis in pugs. This report describes a case of suspected anti‐GFAP encephalitis in a Labrador Retriever with epileptic seizures.

Material and Methods: Retrospective case report.

**Results:** A 3‐year‐old male Labrador Retriever presented in status epilepticus with myoclonic and focal seizures evolving to bilateral tonic‐clonic seizures with severe hyperthermia. The owner reported on an unusually long seizure of >10 min duration with a similar semiology 3 months ago. Laboratory examination showed anemia, thrombocytopenia and positive angiostronglyus antigen test. Magnetic resonance imaging revealed bilateral asymmetric marked T2‐weighted hyperintense, T1‐weighted hypointense hippocampi and a presumably secondary porencephalic lesion in the right piriform lobe without evidence of hemorrhage on gradient‐echo sequences. Cerebrospinal fluid analysis was unremarkable. Initial treatment consisted of phenobarbital, levetiracetam, imidacloprid/moxidectin and anti‐inflammatory dosages of prednisolone. After 3 months, the dog experienced further seizures. Follow‐up MRI revealed persistent but less pronounced hippocampal changes and a new left thalamic T2‐weighted hyperintense lesion. GFAP antibodies in CSF tested positive, suggesting anti‐GFAP autoimmunity. Prednisolone was increased to immunosuppressive dosages, achieving adequate seizure control.

**Conclusion:** As far as we know, this is the first report of anti‐GFAP antibodies in a Labrador retriever with clinical features resembling autoimmune limbic encephalitis. Longitudinal investigations of anti‐GFAP antibodies and potential triggers are warranted to clarify their causality and interactions.

## PO126

### Case report: Diagnostic findings and surgical management of a cauda equina ependymoma in a dog

#### Lara Matiasek^1^; Anna Adrian^2^; Sophie Fish^2^; Andrea Fischer^1^; Kaspar Matiasek^3^

**Keyword:** ependymoma, cauda equina, dog, surgery.

##### ^1^Small Animal Clinic, Centre for Clinical Veterinary Medicine, LMU Munich, Germany; ^2^Small Animal Referral Center, Frontier Veterinary Specialists, Munich, Germany; ^3^Section of Clinical and Comparative Neuropathology, Centre for Clinical Veterinary Medicine, LMU Munich, Germany

Ependymomas are rare spinal cord tumors in dogs. In humans, up to 40% of spinal ependymoma occur as Cauda Equina Ependymoma (CEE). This unique case describes diagnostic findings of a surgically resected CEE in a dog.

A 10.5‐year‐old male Boston Terrier with progressive lower motor neuron paraparesis and occasional fecal incontinence underwent spinal MRI, CT and CSF investigations and subsequent surgery.

Cross sectional imaging revealed a seemingly extradural/ intradural‐extramedullary mass at the level of L6, focally occupying the vertebral canal completely, extending caudally to L7. Cranial to the lesion the spinal cord was displaced dorsolaterally to the right. At the caudal level of L6 the cauda equina was visible ventrolateral to the mass. The L6 lamina appeared atrophied. A modified L6 dorsal laminectomy was performed and slightly extended craniocaudally. Durotomy was necessary to explore the mass, which was rostrally intermingled with four nerve roots in the dural sac and attached to the conus medullaris. About 95% of the mass was resected and attached dura was removed. Histopathology revealed a papillary ependymoma (WHO grade II), poorly proliferative and focally neuroinvasive involving conus medullaris, filum terminale and lumbosacral nerve roots. Postoperatively the gait improved quickly, however urinary reflex dyssynergia and more pronounced fecal incontinence occurred transiently. Eight months after surgery the dog revealed no recurrence of the initially presenting complaint.

This is, to the authors knowledge, the first description of a CEE in a dog, contributing valuable information in veterinary medicine in view of diagnostic imaging findings and outcome with surgical treatment.

## PO127

### *Rasamsonia piperina* disseminated mycosis associated with intracranical granuloma and discospondylitis in a German Sheperd dog

#### Claudia Pauciulo^1^; Giorgia Matteucci^2^; Sara Canal^1^; Daniele Corlazzoli^1^

**Keyword:** Systemic mycosis, MRI, canine, Rasamsonia Piperina

##### ^1^ CNVET, Centro di Neurologia Veterinaria; ^2^ AbLab srls, Laboratorio di Analisi Veterinarie

Introduction: The study aimed to discuss a case of canine disseminated *Rasamsonia piperina* mycosis, characterized by an intracranial granulomatous‐like lesion, lumbosacral discospondylitis, and generalized lymphadenomegaly. To the author's knowledge, this is the first case of systemic mycosis due to *R. piperina* in a dog with intracranial dissemination in Europe.

Methods: A 2‐year‐old female German Shepherd Dog presented with a subacute history of progressive tetraparesis and depression. Neurological examination was consistent with a multifocal localization (intracranial and cauda equina involvement).

The intracranial MRI showed a large left prosencephalic mass lesion with moderate mass effect.

The total body CT scan revealed severe osteolysis of the caudal and cranial end‐plates in the lumbosacral spine at the L7‐S1 intervertebral disc (IVD), associated with a right intersomatic and endocanal abscess‐like/granulomatous inflammatory process.

CSF analysis was unremarkable, and cultures of CSF and L7‐S1 IVD samples were negative for bacterial growth.

Cytology of the prescapular and inguinal lymph nodes, as well as the lumbosacral IVD, confirmed the presence of fungal hyphae.

Molecular identification was consistent with *R. piperina*. Itraconazole and terbinafine were administered, resulting in clinical improvement over the following 4 months.

A brain MRI was repeated 5 months after diagnosis due to worsening of the intracranial signs. The study showed a marked lesion enlargement leading to a severe mass effect. Owners opted for compassionate euthanasia.

Discussion: Molecular identification is essential for obtaining unique fungal identification to avoid misidentifications. Rasamsonia appears to be responsible for severe disseminated mycotic forms with intracranial involvement, making the prognosis reserved.

## PO128

### Cavernous sinus syndrome associated with vestibular syndrome and facial paralysis secondary to lymphoma in a cat

#### Coline Drubay^1^; Aurélie Bourguet^2^; Julien Brune^3^; Julien Miclard^4^; Guillaume Dutil^1^

**Keyword:** Cavernous sinus syndrome, Facial paralysis, Vestibular syndrome, Lymphoma, Cat.

##### ^1^Neurology and Neurosurgery department, CHV Atlantia, Nantes; ^2^Ophtalmology department, CHV Atlantia, Nantes; ^3^Diagnostic imaging department, CHV Atlantia, Nantes; ^4^Institute histopathology, Nantes

A 11‐year‐old, female domestic shorthair cat was presented with a history of anisocoria, ataxia, and dysorexia. Neurological examination revealed an obtunded cat with compulsive behavior associated with a head tilt and head turn to the right. A vestibular ataxia, compulsive circling to the right and drifting to the left were also present. Cranial nerves examination revealed a right‐sided internal and external ophthalmoplegia with a right pupil fixed and dilated, and loss of facial and corneal sensation. Vision was present bilaterally. These findings were consistent with a cavernous sinus syndrome (CSS). Additionally, a facial paralysis was noticed due to absent spontaneous blinking. Neuroanatomical localization was multifocal and involvement of right diencephalon and brainstem was suspected.

Computed tomography examination of the head revealed a large extra axial tumor in the ventral part of the right temporal lobe, extending into the right retro‐orbital space through the oval foramen. Fine needle aspiration was diagnostic for a high‐grade lymphoma. Neurological status worsens over 2 weeks despite palliative therapy, and euthanasia was elicited. Necropsy and histopathology revealed a high‐grade lymphoma invading the calvarium base and the right temporal lobe, infiltrating adjacent meninges, underlying striated skeletal muscles and extending to the periphery of the tympanic bulla and the orbital muscles. Cranial nerves at the level of the temporal bone at the periphery of the middle ear were affected. Immunohistochemistry determined a B‐cell lymphoma.

To the authors' knowledge, this is the first report of a lymphoma causing CSS with vestibular and facial dysfunction in a cat.

## PO129

### Cavernous sinus syndrome associated with vestibular syndrome and facial paralysis secondary to lymphoma in a cat

#### Katarina Kuzek^1^; Andrea Fischer^1^; Kaspar Matiasek^2^; Francesca Cozzi^3^; Rocco Lombardo^3^; Lara A Matiasek^1^

**Keyword:** Cutibacterium acnes, nervous system, intervertebral disc, dog, cat.

##### ^1^Small Animal Clinic, Centre for Clinical Veterinary Medicine, LMU Munich, Germany; ^2^Section of Clinical and Comparative Neuropathology, Centre for Clinical Veterinary Medicine, LMU Munich, Germany; ^3^La Clinica Neurologica Veterinaria NVA, Milan, Italy

Introduction: Cutibacterium (C) acnes, a member of the skin microbiota and known as potential laboratory contaminant, is an increasingly recognized source of invasive infections in humans, including those of the nervous system and intervertebral discs.

Methods: Retrospective case series of four animals with neurologic signs and suspected associated C. acnes, confirmed through 16S rDNA PCR (surgical specimen) or bacterial culture (CSF).

Results: In an 8‐year‐old crossbreed dog with subacute tetraparesis (dog 1) and a 4‐year‐old Portuguese Water Dog with chronic lumbar pain (dog 2), both due to intervertebral disc extrusion, C. acnes could be isolated from surgically removed disc material. Dog 1 revealed pyogranulomatous inflammation of extruded material, whereas in dog 2 this was not the case. Dog 3 was a 3‐year old crossbreed with acute forebrain signs due to a space‐occupying lesion, where CSF revealed polymorphonuclear pleocytosis with intracellular bacteria and positive anaerobic culture for C. acnes. Treatment including antibiotics resulted in variable improvement in all dogs. Dog 3 was euthanized due to unrelated disease after 7 weeks. In a 16‐year old cat with chronic forelimb paresis and bilateral enlarged brachial plexus, nerve biopsy revealed pyogranulomatous neuritis and was positive for C. acnes. No follow‐up information could be obtained.

Discussion: C. acnes may be an underreported cause of infectious disease in small animal neurology, possibly due to its slow growth in cultures and the difficulty in determining its role as a pathogen or contaminant. This case series highlights the importance of considering C. acnes as a potential causative agent.

## PO130

### Intraocular malignant nerve sheath tumor in a brown eyed labrador retriever

#### Lauren Salter^1^; Emma Scurrell^2^; Thomas Mignan^1^

**Keyword:** Canine, Ophthalmology, Neurology, Veterinary, Neoplasia

##### ^1^Dovecote Veterinary Hospital; ^2^Cytopath Ltd

A 6‐year‐old, male, neutered, brown eyed Labrador Retriever was presented with an intraocular pale pink mass in the left eye. Enucleation of the globe was performed and the mass was submitted for histopathology. Histology and immunohistochemistry confirmed the mass as a malignant nerve sheath tumor. There was no evidence of mass recurrence and general and neurological examination remained normal at 9‐month follow‐up. While the majority of intraocular neoplasms are benign, a malignant neoplasm must be considered in all cases of intraocular mass. Differentiation between benign and malignant intraocular neoplasms is challenging and often requires enucleation with histopathology to confirm diagnosis. Tumor staging should be considered in the event of a malignant nerve sheath tumor. Finally, uveal malignant nerve sheath tumor should not only be considered in blue eyed dogs, but also in brown eyed dogs as in the present case.

## PO131

### Prevalence of neurological diseases in a population of 364 French bulldogs seen in two UK'S referral centres from 2019 to 2021

#### Michał Mól^1^; Roberto Rizzo^4^; Elisa Pompermaier^2^; Lisa Castellano^3^; Giuseppe Vitello^1^; Massimo Mariscoli^1^

**Keyword:** French Bulldogs, Intervertebral Disc Extrusion, Neurology, Myelopathy, Encephalopathy.

##### ^1^Paragon Referrals; ^2^Wear Referrals; ^3^Abbey House Vets; ^4^Scarsdale Vets

**Introduction:** French Bulldogs (FBs) have become increasingly popular in the United Kingdom. The objective of the study was to report the prevalence of neurological disorders in a large FBs population.

**Methods:** The aim of the study was to retrospectively evaluate the clinical records of FBs investigated for neurological diseases between January 2019 and December 2021 in two UK's Referral Hospitals. Definitive diagnosis was established on signalment, history, clinical presentation, advanced diagnostic imaging, and ancillary tests according to the initial findings.

**Results:** 364 FBs were presented for neurological conditions (45.95% of admitted FBs in the same period). Myelopathies were the most common conditions (*n* = 278.76.3%).

Intervertebral disc extrusion (IVDE) was the prevailing pathology (*n* = 175.48%). The most affected IVD were L3‐L4 (*n* = 37.21% of IVDEs) and C3‐C4 (*n* = 36.20.6% of IVDEs). IVD protrusion was diagnosed in 38 dogs (10.4%) and C2‐C3 was the most affected disc (*n* = 11.28.9% of IVDPs).

The median age for IVDE was 3.49 years (mean 3.6), and 3.9 for IVDP (mean 4.3) and 62.2% were males. Other common myelopathies were syringohydromyelia (*n* = 35,9.6%). subarachnoid diverticulum (*n* = 17.4.7%) and concurrent vertebral malformation (*n* = 126.34.6%).

Encephalopathy affected 45 dogs (12.3%). In this group idiopathic epilepsy was diagnosed in 13 dogs (3.6%), meningoencephalitis of unknown origin in 12 (3.3%) and intracranial neoplasia in 9 FBs (2.5%). Peripheral vestibular syndrome and otitis media‐interna was also a frequent diagnosis (*n* = 38.10.4%).

**Discussion/conclusion:** This evidence‐based study can improve awareness of the FBs health problems. FBs suffering from IVDE were overrepresented and younger than previously described.

## PO132

### Application of biomechanical 3D‐printed spine model for pre‐surgical planning in a feline vertebral neoplasm

#### Anna Tauro^1^; John Macri^1^; Christopher Mariani^1^; Tracy Gieger^1^; Satya Konala^1^; Ola Lars Harrysson^1^

**Keyword:** 3D‐printed spine model, biomechanical, hemilaminectomy, surgical planning, bone tumor, vertebral, neoplasm, cat, feline, chondrosarcoma, chondroblastic osteosarcoma, Stratasys.

##### ^1^North Carolina State University

**Introduction/Purpose:** The aim of this study is to report the usefulness of biomechanical 3D printing to enhance surgical precision and planning.

**Methods:** A 2‐year‐old, male neutered, domestic short‐hair cat presented with a 1‐month history of non‐specific pain, inappetence, left Horner syndrome, paraparesis with plantigrade gait. Advanced imaging revealed a heterogeneous contrast‐enhancing mass, centered dorsally to the T1‐T2 intervertebral disc space, causing severe left‐sided spinal cord compression. Materialize Mimics 3D medical image processing software was used to generate a highly accurate 3D spine model using magnetic resonance and computed tomography data. A Stratasys J850 Digital Anatomy printer was used to create a 3D replica of the spine, matching the patient's anatomy and tissue density. A T1‐T3 left‐sided hemilaminectomy and mass removal were simulated using this model.

**Results:** The model accurately simulated tissue density observed during surgery. During the simulation, the extradural mass was found to be contiguous with the T1 vertebral body and extending along the vertebral canal, reaching the T2‐T3 intervertebral disc space. The model facilitated the identification of landmarks during surgery, contributing to achieving optimal spinal cord decompression. Histopathology of the mass supported the diagnosis of a bone tumor (chondrosarcoma > > chondroblastic osteosarcoma). Alkaline phosphatase staining, useful to differentiate osteosarcoma from other mesenchymal tumors, could not be performed due to the unavailability of an unstained cytologic specimen. The patient is currently receiving radiation therapy and has been in remission for 3 months.

**Discussion/Conclusion:** Biomechanical 3D‐printed spine models appear to effectively simulate in‐vivo surgery, enhancing surgical planning and precision.

## PO133

### Resection of a multilobular osteosarcoma of the occipital bone and reconstruction of the skull with a customized 3D printed implant in an Australian Shepherd

#### Josephine Dietzel^1^; Theresa Kalliwoda^1^; Peter Böttcher^2^; Shenja Loderstedt^1^

**Keyword:** Multilobular osteosarcoma, 3D implant.

##### ^1^Department for Small Animals, Faculty of Veterinary Medicine, Leipzig University, Germany; ^2^Competence Centre for Small Animals Leipzig, Leipzig, Germany

**Introduction:** A nine‐year‐old male Australian Shepherd was presented due to minor coordination problems. The symptoms had been present for approximately 2 years and had been progressive over the last 2 weeks. The neurological examination revealed a mild left sided head tilt, cerebellar ataxia with generalized hypermetria, titubation and generalized hopping deficits, indicating a cerebellar neurolocalisation.

**Methods:** An MRI was carried out and revealed an extra‐axial mass, measuring approximately 5.3 × 6.1 cm, originating from the occipital bone. This mass caused severe cerebellar and moderate compression of the occipital lobe and diencephalon. Surgery was planned in collaboration with animalTECH© with regard to position and extent of the craniotomy. For the subsequent defect coverage, animalTECH© modeled an artificial occiput, printed in PLA (Greentec Pro, Extrudr FD3D GmbH, Austria).

**Results:** Histopathological examination of a tru‐cut‐needle biopsy confirmed the suspected diagnosis of multilobular osteosarcoma. The craniotomy was guided through a patient‐specific milling template applied to the occiput. A life‐size 3D print of the caudal skull calvaria was employed to fulfill the requirements of the 3D surgical planning. The neoplasm was resected using an ultrasonic aspirator and a hollow chisel pliers, which enabled macroscopically complete removal of the tumor without damaging the adjacent brain tissue. The defect was closed with the customized 3D implant (animalTECH©). The patient was discharged 4 days after surgery in good general health, ambulatory with moderate cerebellar ataxia.

**Conclusion:** We present a customized, safe method for the resection of an extensive skull neoplasia using 3D printed templates and implants.

## PO134

### Magnetic resonance and fundoscopy findings of presumptive bilateral optic nerve malformation or prenatal atrophy in a kitten

#### Alfredo Recio^1^; Angels Ribas^2^; Ines Carrera^2^; Tamara Heredia^2^

**Keyword:** Optic nerve malformation atrophy.

##### ^1^ Clinica Veterinaria Levante; ^2^ ARS Veterinaria

Congenital optic nerve malformations (CONM), hypoplasia or aplasia, are uncommon embryonic development diseases and have been barely described in cats. In dogs, CONM have a low presentation rate and have been mainly reported in Miniature poodle breed. Prenatal or early postnatal atrophy have been also rarely described in cats and may show similar clinical and diagnostic presentation, being necessary a final histopathological confirmation. We describe a presumptive case of CONM/atrophy in a kitten based in clinical, ophthalmologic and MRI findings.

An 8‐months‐old Short haired cat was referred for a non‐progressive disorientation from birth. Physical examination showed pelvic limbs malformations and bilateral mydriasis. Neurological examination revealed bilateral oscillating nystagmus, absence of menace response, pupillary and dazzle reflexes, consistent with an optic or prechiasmatic blindness.

Ophthalmological examination showed bilateral abnormal optic nerve papilla, compatible with both optic nerve atrophy and coloboma. Tapetal vascularization were marked attenuated and revealed diffuse hyporeflectivity, while non‐tapetal area showed depigmentation areas. Magnetic resonace (MRI) was performed, showing a severe bilateral optic nerve atrophy/hypoplasia from the optic disc to a size reduced optic chiasm. Postchiasmatic visual pathways and structures didn't show any abnormality.

To the author's knowledge, this is the first case describing MRI findings associated to clinical and ophthalmological signs of a suspected CONM or prenatal atrophy in a cat. Neurological and ophthalmological examination and MRI allow in vivo presumptive diagnosis of these diseases. Etiology or pathogeny was not possible to establish in our case, being necessary a histopathological study for confirmation.

## PO135

### Interobserver agreement of proprioception examination in a cohort of dogs after spinal surgery

#### Vivian Weiss^1^; Shenja Loderstedt^2^

**Keyword:** interobserver agreement, proprioception.

##### ^1^Vivian Weiss; ^2^Shenja Loderstedt

**Introduction:** Thoracolumbar intervertebral disc extrusion (IVDE) is the most common spinal cord disease in dogs. Proprioceptive tests are used to defined the neurological status of myelopathic dogs. This cohort study investigates the interobserver agreement (IOA) of proprioception examination in dogs following T3‐L3 spinal surgery.

**Methods:** Client‐owned dogs undergoing thoracolumbar hemilaminectomie for Hansen Type I IVDE between August 2022 to January 2024 were included. Two investigators (VW, SL) conducted follow‐up examinations 2 days, 30 days and 4‐6 months post‐surgery. For statistics Cohens Kappa and paired t‐ test was used.

**Results:** Of the 44 dogs included 75% were Dachshunds or French Bulldogs with an average age of 5 years. The overall IOA of the neurological examination (proprioception and segmental reflexes) was 0.81 (almost perfect). For all four limbs the agreement was 0.73 (substantial) for hopping and 0.73 (substantial) for paw placement. The IOA for just the pelvic limbs (PL) was 0.49 (moderate) for hopping and 0.61 (substantial) for paw placement. The proprioceptive examination showed an agreement of 0.52 (moderate) for hopping reaction of the left PL and 0.47 (moderate) for the right PL. The agreement in paw placement was 0.60 (moderate) for the left PL and 0.63 (substantial) for the right PL. All dogs improved significantly regarding modified frankel score.

Discussion/ **Conclusion:** The study describes a moderate to almost perfect IOA of proprioception examination for dogs after spinal surgery.

## PO136

### Home‐management complication rate of indwelling urinary catheters in patients with impaired mobility

#### David Dauden^1^; Emili Alcoverro^3^; Simone Spinillo^3^; Marco Ruggeri^3^; Rocio Orlandi^2,3^

**Keyword:** Indwelling urinary catheters, Urinary tract infections, Impaired mobility, Myelopathy

##### ^1^Pride Veterinary Referrals; ^2^ ChesterGates; ^3^ ChesterGates Veterinary Specialists

**Introduction:** Urinary catheters are frequently used in veterinary medicine to monitor urine output and manage bladder dysfunction, particularly in dogs suffering from any type of myelopathy. This study aimed to evaluate the prevalence of complications in dogs discharged with an indwelling urinary catheter.

**Methods:** 79 dogs with impaired mobility were included. These patients presented to a referral centre in the UK, from October 2022 to October 2023.

**Results:** Urinary tract infection (UTI) prevalence was 16.46%, aligning with the lower end of previously reported ranges. We found that a longer duration (mean of 9.7 days) and an increased number of required replacement times (mean of 1.8 times) were associated with a higher risk of UTI. The primary complication was catheter dislodgement (30.38%), impacting owner compliance and costs, but not being linked to UTIs. One case was diagnosed with a multidrug‐resistant E. coli. Two patients were diagnosed with pyelonephritis which progressed to septicemia, leading to a fatal outcome in one of them. Other complications included pigmenturia with no associated UTI (7.59%), urethral prolapse, urethral rupture and preputial infection (1.27% each).

**Discussion/Conclusion:** Most patients had impaired mobility and bladder dysfunction, making it challenging to differentiate between catheter‐associated and condition‐associated UTIs. Although further research is required to optimize catheter management and minimize complications, bladder management with indwelling urinary catheters at home appeared to be well‐tolerated by owners. Nevertheless, each patient and case should be assessed individually as fatal consequences could occur.

## PO137

### Empty sella syndrome in a puppy dog secondary to traumatic brain injury

#### Carmen Aires Serrano^1^; Alba Farré Mariné^1^; Alejandro Luján Feliu‐Pascual^1^

**Keyword:** Empty Sella Syndrome, Traumatic brain injury, Puppy Dog.

##### ^1^Aúna Especialidades Veterinarias IVC Evidensia

The empty sella syndrome, rare in dogs and cats, is characterized by fluid‐filled sella turcica, due to intrasellar herniation of the subarachnoid space. Primary or secondary causes such as surgery, radiation, or pituitary diseases can be associated with endocrine disturbances, and involve treating any underlying hormonal imbalances and addressing clinical signs.

A 6‐month‐old female entire Ratonero dog suffered a severe traumatic brain injury (TBI) 1 month earlier and recovered. As a result, the dog developed bilateral blindness and polyuria/polydipsia (6 L/day).

The neurological examination showed absent menace response and pupillary reflexes in both eyes, locating the lesion at the level of the middle cranial fossa (optic chiasm ± hypothalamus) and considering as main differential diagnoses empty sella secondary to TBI, or a congenital malformation (e.g., Ratke's cyst).

In a brain CT, the sellar region was filled mainly with fluid, the hypophysis appeared absent and a solution of continuity in the right region of the pre‐sphenoid bone and in the right parietal bone compatible with fractures were evident.

Since there was little displacement of the bone fractures, conservative treatment with synthetic antidiuretic hormone (desmopressin) to reduce the polyuria was established.

Three months after diagnosis, the dog remains clinically stable with bilateral blindness and water intake has reduced to 250 mL/day.

To the authors' knowledge, this is the first reported case of empty sella secondary to TBI in dogs. This case highlights the importance of considering empty sella syndrome in the differential diagnosis of neurological abnormalities following TBI in veterinary medicine.

## PO138

### The relationship between electroencephalographic brainwave morphology and body weight in healthy canines and dogs with different CNS disorders

#### Laura Brewińska^1,2^, Karolina‐Owsińska‐Schimdt^2^, Aleksandra Banasik^1,2^, Paulina Drobot^2^, Dorota Durczok^2^, Marcin Wrzosek^1,2,3^

##### ^1^Department of Internal Diseases with a Clinic for Horses, Dogs, and Cats, Faculty of Veterinary Medicine, Wrocław University of Environmental and Life Sciences, Wrocław, Poland; ^2^ NeuroTeam Veterinary Specialist Clinic, Wrocław, Poland; ^3^Vetimaging, Veterinary Computed tomography, Poland

**Introduction:** Electroencephalographic (EEG) examination is a non‐invasive diagnostic tool useful in epilepsy diagnosis. However, universal protocols to perform and analyze EEG examinations are still lacking. One of the difficulties in developing such algorithms is a huge diversity in body types of veterinary patients. Background activity (BGA) is an important feature providing information about brain condition. In humans it was shown that larger heads tend to produce slower alpha rhythms. Previous studies in dogs shown that high voltage, low‐frequency BGA was more frequent in dogs with metabolic encephalopathy and intracranial pathology The aim of this study was to determine how animal's body weight influence BGA in healthy dogs and those with different brain conditions.

**Methods:** Study group consisted of 121 dogs. Examinations were performed under medetomidine sedation. The EEG rhythm was measured in 5th, 10th and 20th minute of the examination in rostral and caudal derivatives (F3, F4, O1, and O2). The patients were divided into four groups: healthy dogs and dogs with idiopathic epilepsy, intracranial pathology and metabolic conditions. The correlation between body weight, frequency and amplitude of the brainwaves was performed.

**Results:** The amplitude was significantly larger in smaller dogs. The study also shown a significant difference in amplitude in dogs with intracranial pathology compared to the ones with idiopathic epilepsy and between rostral and caudal derivatives.

**Discussion:** EEG BGA might provide important information about brain condition but could differ between derivatives and animals of a different sizes.

